# Simple synthesis of new imidazopyridinone, pyridopyrimidinone, and thiazolopyridinone derivatives and optimization of reaction parameters using response surface methodology†

**DOI:** 10.1039/c9ra06054e

**Published:** 2019-09-25

**Authors:** Fahimeh Sadat Hosseini, Mohammad Bayat

**Affiliations:** Department of Chemistry, Faculty of Science, Imam Khomeini International University Qazvin Iran bayat_mo@yahoo.com m.bayat@sci.ikiu.ac.ir +98 28 33780040

## Abstract

The reaction between ketene animal/ketene *N*,*S*-acetals (derived from diamines/cysteamine hydrochloride and 1,1-bis(methylthio)-2-nitroethene) with aromatic aldehydes, and Meldrum's acid led to the title compounds. The reaction conditions were optimized using response surface methodology (RSM). The two independent variables (temperature and water content of aqueous ethanol), and the responses (yield of product and reaction time) were studied. The range of each parameter selected was: *T* = 25–100 °C and water = 0–100%. The optimal values were: *T* = 72 °C and water = 33%. This work offers significant advantages including use of a green solvent, experimental simplicity, absence of catalyst, a simple work-up and purification process, moderate to good yields, and preparation of potentially bioactive compounds.

## Abstract 2

The one-pot reaction of various diamines with cysteamine hydrochloride or 1,1-bis(methylthio)-2-nitroethene, aromatic aldehydes, and Meldrum's acid led to pyridone compounds in good yields. The reaction conditions were optimized using response surface method.

## Graphical abstract

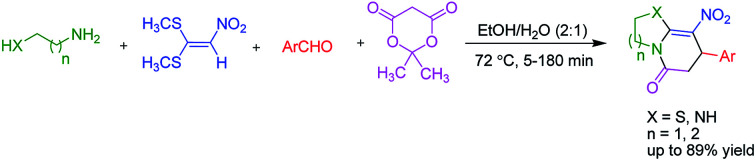

## Introduction

Response surface methodology (RSM) is the most popular optimization method used in recent years. RSM is a powerful mathematical and statistical technique which has been widely used for designing experiments and empirical building models with minimal experimental number. This method is exclusively used to examine the surface, or the relationship between the response and the factors affecting the response. The application of RSM to design optimization is aimed at reducing the cost of expensive analysis methods and their associated numerical noise.^1–6^

Nitrogen and sulfur containing heterocyclic compounds broadly exist in various natural and synthetic products, and play vital roles in medicinal chemistry.^7–10^ Among these heterocycles, pyridine derivatives, such as imidazopyridine, pyridopyrimidine, and thiazolopyridine derivatives have received considerable attention over the past years due to their diverse biological activities and clinical applications.^11,12^ Imidazopyridine derivatives have shown a broad range of interesting biological activities, such as antifungal, antitumor, antiviral, antibacterial, anti-HIV,^13^ antipyretic, analgesic, hypnoselective, anxioselective, anti-inflammatory, anticonvulsant, antiulcer, immunomodulatory^14^ activities; also pyridopyrimidine derivatives^15^ are found to exhibit a broad spectrum of potent antibacterial, antiallergic, antimicrobial,^12^ anti-inflammatory, analgesic,^16,17^ tyrosine kinase, calcium channel antagonists,^8,18^ tuberculostatic, antileishmanial, antitumor,^19^ antifolate, antihypertensive, hepatoprotective,^20^ anticancer^21^ properties; and thiazolopyridine derivatives are associated with a wide range of biological activities such as analgesic, antioxidant,^11^ anticancer,^22^ anti-inflammatory,^23^ antihypertensive,^24^ antibacterial, and antifungal^25,26^ properties.

**Scheme 1:**
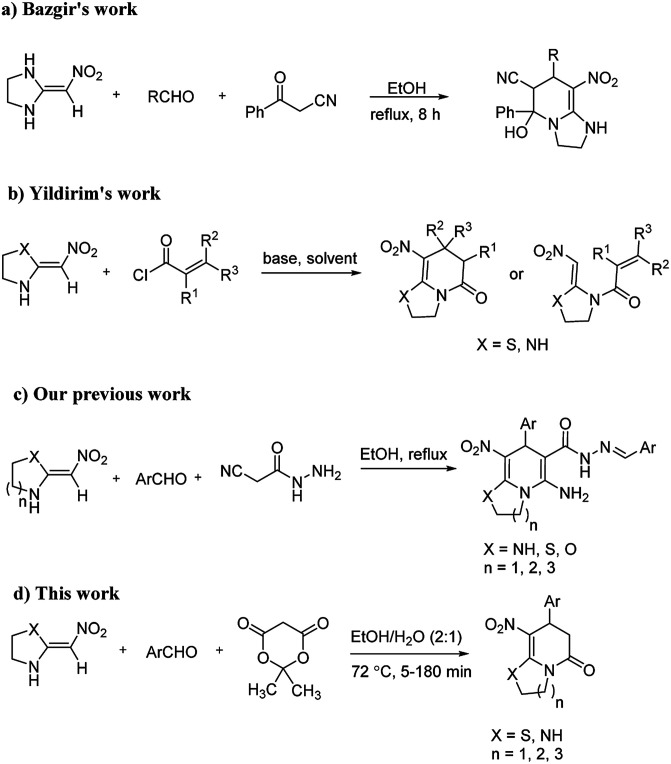
Use of enamines derived from 1,1-bis(methylthio)-2-nitroethene in organic synthesis.

The numerous attractive features of enamines or dienamines derived from the 1,1-bis(methylthio)-2-nitroethene have made them important intermediates for the construction of a wide variety of S- and N-heterocyclic systems.^27–33^ Hence during recent years, they have been used for the synthesis of various fused heterocycles and drug-like compounds. For examples in 2013 Bazgir *et al.* described a one-pot, four-component synthesis of a novel class of functionalized imidazo[1,2-*a*]pyridine derivatives starting from readily available inputs including diverse diamines, 1,1-bis(methylthio)-2-nitroethene, aldehydes and activated methylene compounds in EtOH under reflux conditions (Scheme 1, Entry a).^34^ In 2014 Yildirim *et al.* reported a base-catalyzed one-pot cyclocondensation reaction of acryloyl and cinnamoyl chlorides with β-nitroenamine derivatives under mild basic conditions, which affords target thiazolopyridinone or imidazopyridinone derivatives (Scheme 1, Entry b).^35^ In 2017 and 2018 we developed an efficient, one-pot, multi-component synthesis of imidazopyridine, pyridopyrimidine, thiazolopyridine, and oxazolopyridine fused heterocyclic systems *via* reaction of various diamines, cysteamine hydrochloride, ethanolamine, 1,1-bis(methylthio)-2-nitroethene, cyanoacetohydrazide and aromatic aldehydes in ethanol at reflux (Scheme 1, Entry c).^36,37^ Herein we report synthesis of a new class of imidazopyridinone, pyridopyrimidinone, and thiazolopyridinone derivatives through a simple one-pot, multi-component reaction of diverse diamines or cysteamine hydrochloride, 1,1-bis(methylthio)-2-nitroethene, aromatic aldehydes, and 2,2-dimethyl-1,3-dioxane-4,6-dione (Meldrum's acid) in ethanol/water (2 : 1) at 72 °C (Scheme 1, Entry d). RSM in conjunction with a central composite design was used for modeling and optimizing the synthetic process.

## Result and discussion

**Scheme 2:**
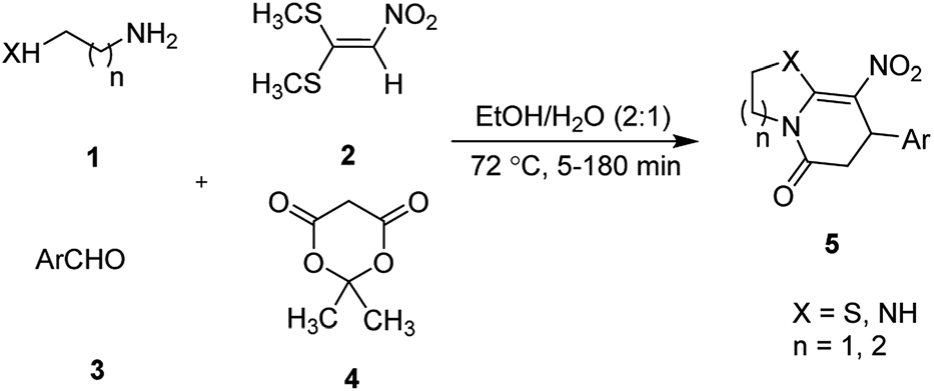
Synthetic scheme for the products 5a–o.

The one-pot, multi-component reaction of various diamines or cysteamine hydrochloride 1 with 1,1-bis(methylthio)-2-nitroethene 2, aromatic aldehydes 3, and 2,2-dimethyl-1,3-dioxane-4,6-dione (Meldrum's acid) 4, in EtOH/H_2_O (2 : 1) at 72 °C, gave imidazopyridinone, pyridopyrimidinone, and thiazolopyridinone derivatives 5 in moderate to good yields (Scheme 2).

A reaction condition is optimized using response surface methodology (RSM). A central composite design with five replicates at the center point requiring 13 treatments was used. The two independent variables (temperature (*A*) and water content of aqueous ethanol (*B*)), levels (Table 1), and the results obtained after running the experiments are represented in Table 2. The following responses were analyzed: yield of product (*R*_1_) and reaction time (*R*_2_).

**Table 1:** Selected variables and levels used in central composite design

Variables	Code	Units	Levels		
			−1	0	+1
Reaction temperature	*A*	°C	25	62.5	100
Water content of aqueous ethanol	*B*	%	0	50	100

**Table 2:** Process variables and experimental data for two factors, three levels response surface design^a^

Run	*A* (°C)	*B* (%)	*R*_1_ (%)	*R*_2_ (min)
1	62.50	50.00	80	7
2	35.98	85.36	50	5
3	89.02	14.64	77	5
4	62.50	50.00	83	6
5	25.00	50.00	45	11
6	62.50	50.00	84	5
7	100.00	50.00	77	5
8	62.50	0.00	90	5
9	62.50	50.00	84	5
10	89.02	85.36	62	6
11	62.50	100.00	37	11
12	62.50	50.00	82	5
13	35.98	14.64	64	5

a *A* = temperature (°C), *B* = water content of aqueous ethanol (%), *R*_1_ = yield of reaction, *R*_2_ = reaction time.

Optimum conditions with respect to yield, purity of product and reaction time were as follow: temperature 72 °C, water content of aqueous ethanol 33%. Verification experiments, carried out at the predicted conditions showed values reasonably close to those predicted and further confirmed the adequacy of predicted models.

On the basis of the central composite design, the cubic model relationship between the experimental yield (*R*_1_) and the process variables (temperature (*A*) and water content of aqueous ethanol (*B*)) in coded units is obtained from eqn (1).

1 *R*_1_ = 82.60 + 11.31*A* − 18.74*B* − 0.25*AB* − 10.55*A*^2^ − 9.30*B*^2^ + 11.49*A*^2^*B* − 5.06*AB*^2^

where *R*_1_ represents the experimental yield of the reaction, then *A* (temperature) and *B* (water content of aqueous ethanol) are the coded variables in the reaction.

**Fig. 1:**
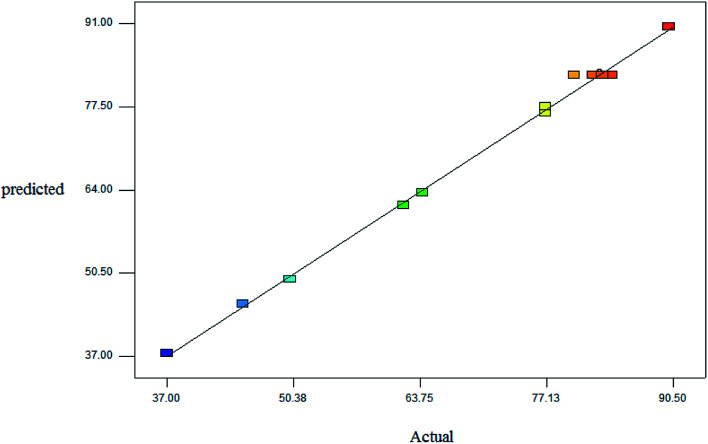
Linear correlation between the actual and predicted yield.

Fig. 1 illustrates the good linear correlation between the actual and predicted yield and sufficient accuracy of the forecast values. Therefore, the models can be used to predict the yield of product successfully.

The ANOVA for response surface cubic models are reported in Table 3. The model *F*-value of 189.50 implies the model is significant. The “Lack of Fit *F*-value” is 0.71 then it is not significant relative to the pure error. Non-significant lack of fit is good and we want the model to fit. The “Pred *R*-Squared” of 0.9586 is in reasonable agreement with the “Adj *R*-Squared” of 0.9910; *i.e.*, the deviation is about 0.3.

**Table 3:** ANOVA for response surface cubic model

Source	Sum of square	Degree of freedom	Mean square	*F* value	*p*-Value, prob > *F*
Model	3501.88	7	500.27	189.50	<0.0001 significant
*A*-temperature	512.00	1	512.00	193.94	<0.0001
*B*-percentage of water in ethanol	1404.50	1	1404.50	532.01	<0.0001
*AB*	0.25	1	0.25	0.095	0.7707
*A* ^2^	774.28	1	772.28	293.29	<0.0001
*B* ^2^	601.67	1	601.67	227.91	<0.0001
*A* ^2^ *B*	263.96	1	263.96	99.99	0.0002
*AB* ^2^	51.28	1	51.28	19.43	0.0070
*A* ^3^	0.000	0			
*B* ^3^	0.000	0			
Residual	13.20	5	2.64		
Lack of fit	2.0	1	2.00	0.71	
Pure error	11.20	4	2.80		0.4456 not significant
Correlation total	3515.08	12			

**Fig. 2:**
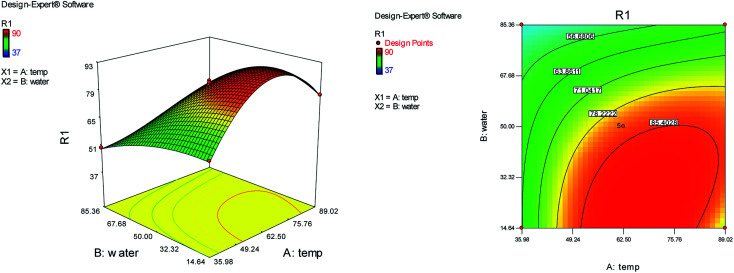
The effects of temperature and water content of aqueous ethanol on the total reaction yield.

The effects of temperature and water content of aqueous ethanol on the total reaction yield are shown in Fig. 2. The total yield varied from 37% to 90%. As temperature increased, the yield increased. The changes in yield *versus* the water content of aqueous ethanol is minor compared to that of temperature. The highest yield (90%) was obtained at 62.5 °C and at 0% water. The lowest yield (37%) was at 62.5 °C and at 100% water.

**Fig. 3:**
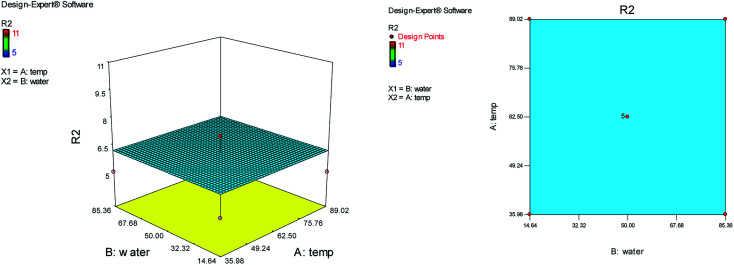
The effects of temperature and the water content of aqueous ethanol on the total reaction time.

The effects of temperature and the water content of aqueous ethanol on the total reaction time are shown in Fig. 3. The analysis of total reaction time (*R*_2_) showed the significant Lack of fit and it is not good. So the temperature and water content of aqueous ethanol have no effect on the reaction time and the total reaction time was almost identical and short in any case.

The scope of the reaction substrate was explored under optimized conditions. Several kind of aromatic aldehydes, and various diamines were tolerated. The reaction was completed after 5–180 min to afford corresponding S- and N-heterocyclic systems 5a–p, in moderate to good yields (50–89%). As shown in Table 4, reactions with diamines, usually resulted in good yields and short reaction times. Reactions with cysteamine hydrochloride, resulted in low yields and long reaction times. Also the structures of the aldehydes had obvious influence on the yield of product and reaction time. Reactions involving aromatic aldehydes usually went smoothly and resulted in good yields and short reaction times. Reaction involving heteroaromatic aldehyde (such as furfural) and aliphatic aldehyde (such as butyraldehyde), on the other hand, were normally slow and resulted in low and trace yields, respectively. When the 4-pyridinecarboxaldehyde was used the reaction did not work. The substitutions of the aromatic aldehydes had some influence on the yields and reaction times. Reaction with aromatic aldehydes carrying an electron-withdrawing group, such as chloro and nitro groups, usually resulted in good yields and short reaction times; those carrying electron-donating groups such as methoxy group, resulted in relatively poorer yields. The reaction did not work, when the reaction was performed using ethanolamine, cyclohexane-1,2-diamine and benzene-1,2-diamine.

**Table 4:** One-pot, multi-component synthesis of imidazopyridinone, pyridopyrimidinone, and thiazolopyridinone derivatives 5a–p

Entry	Aldehyde^a^	Amine^a^	Product	Time (min)	Yield (%)
1	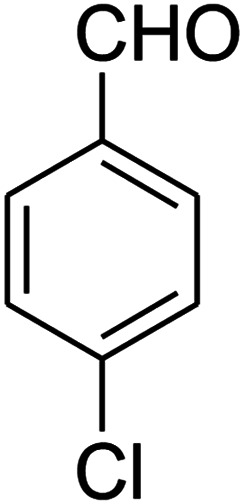	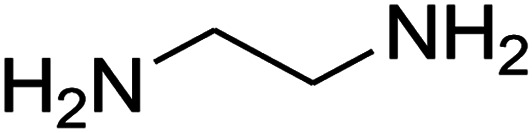	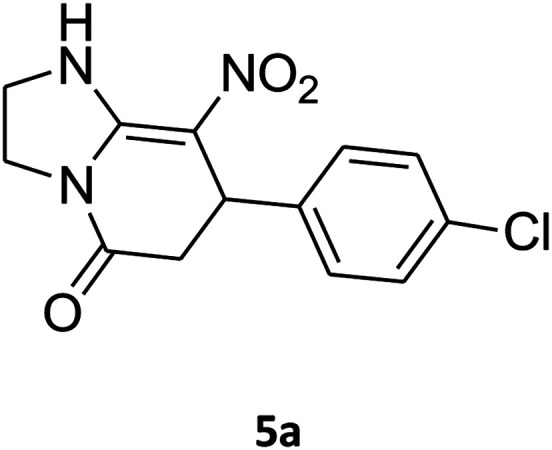	5	88
2	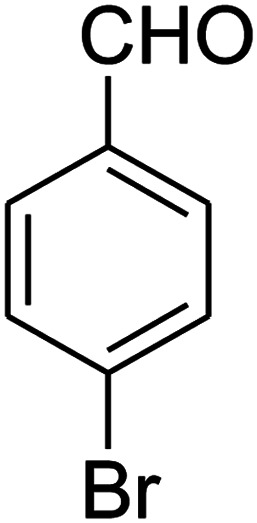	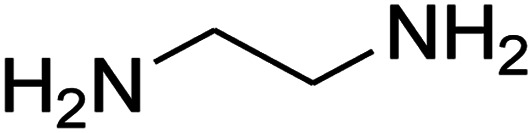	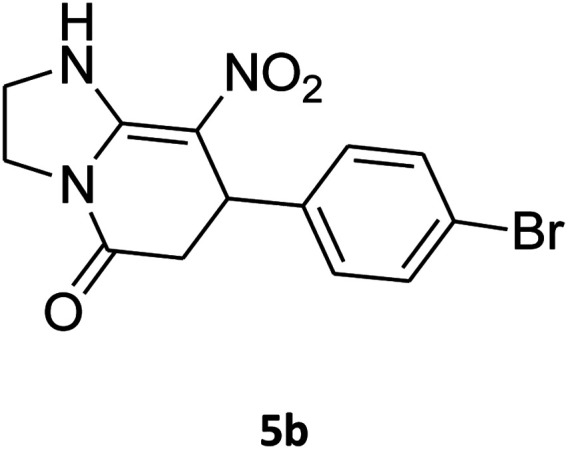	5	87
3	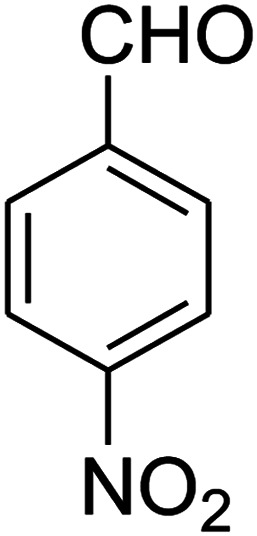	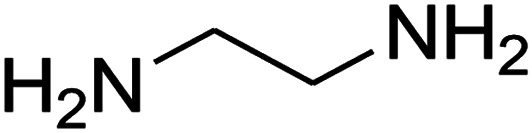	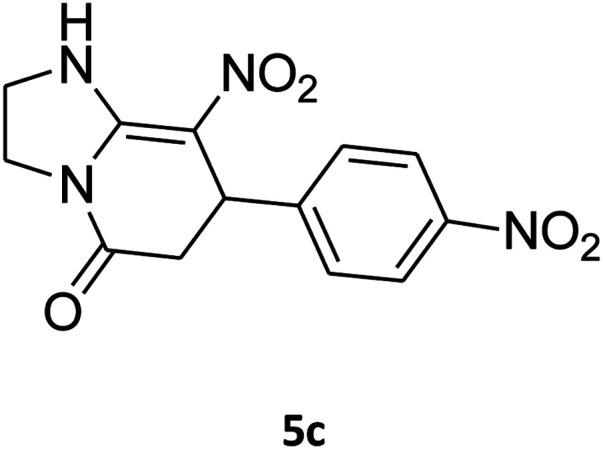	10	89
4	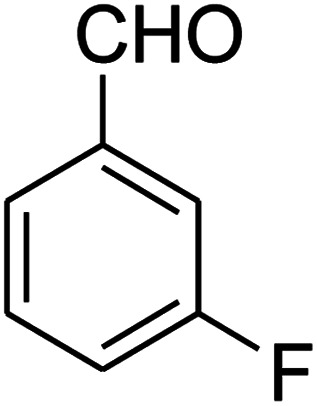	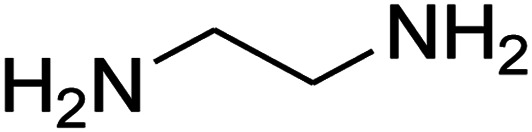	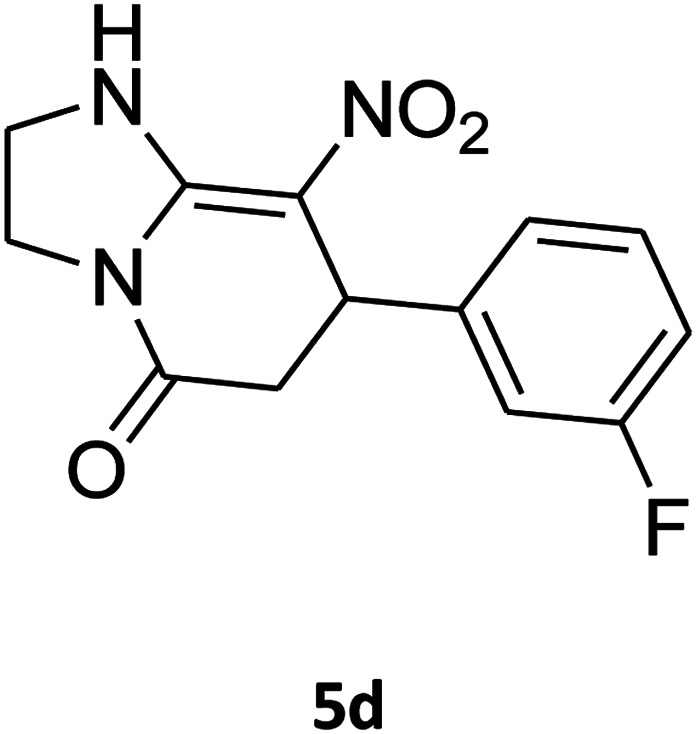	20	75
5	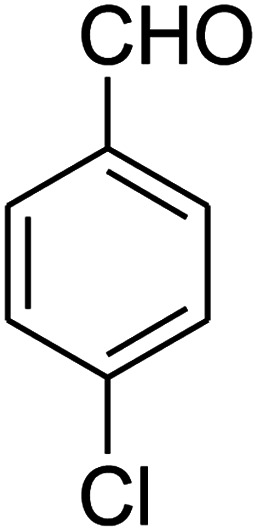	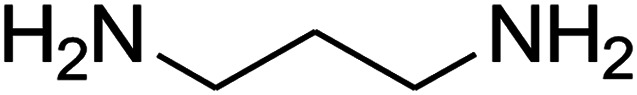	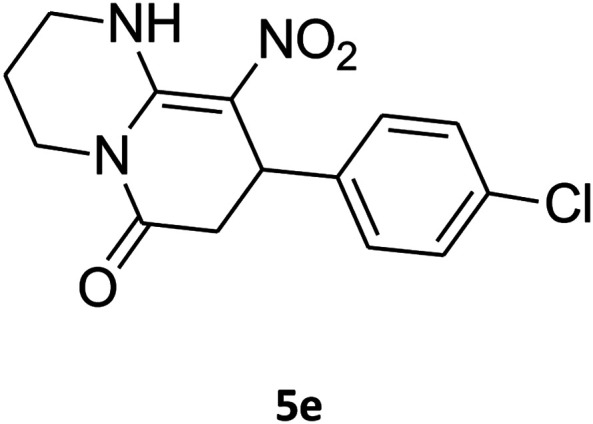	5	85
6	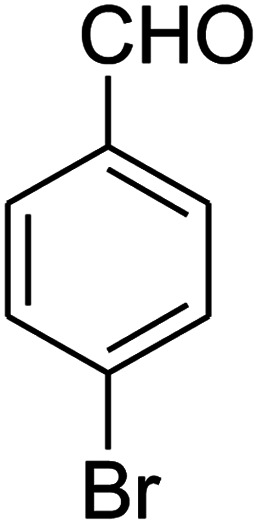	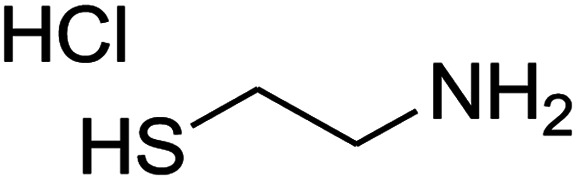	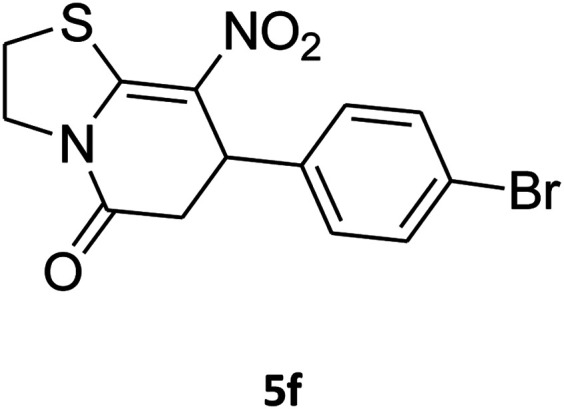	120	65
7	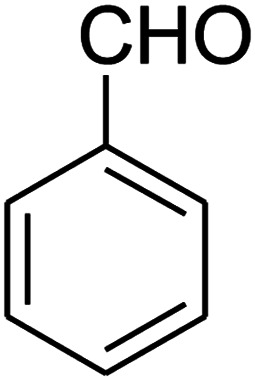	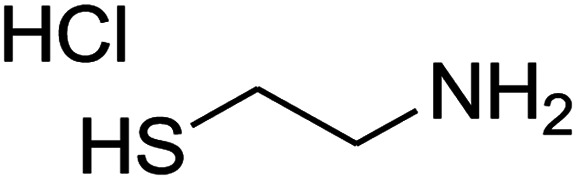	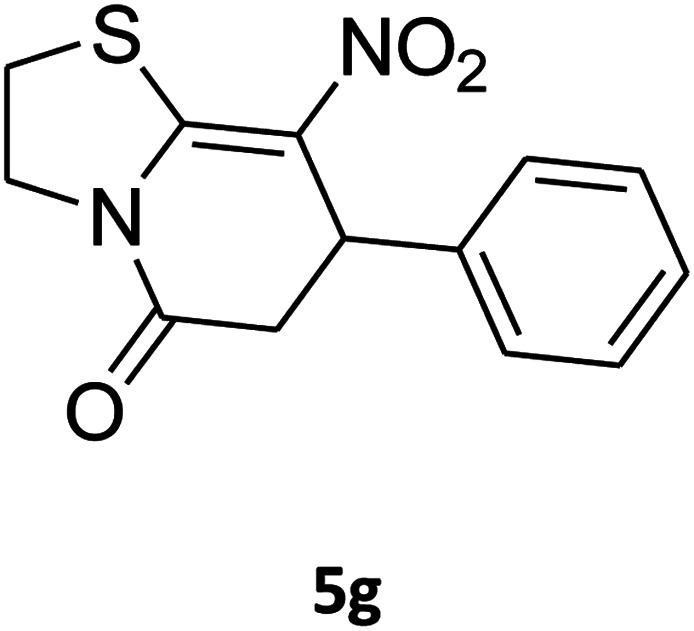	180	53
8	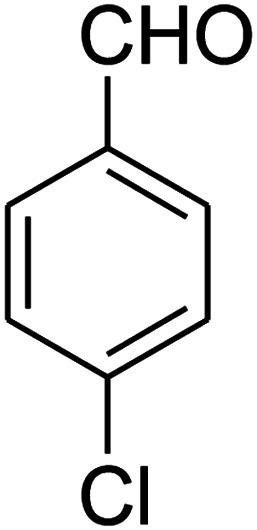	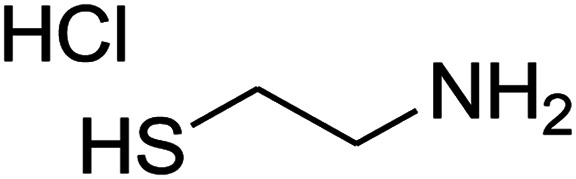	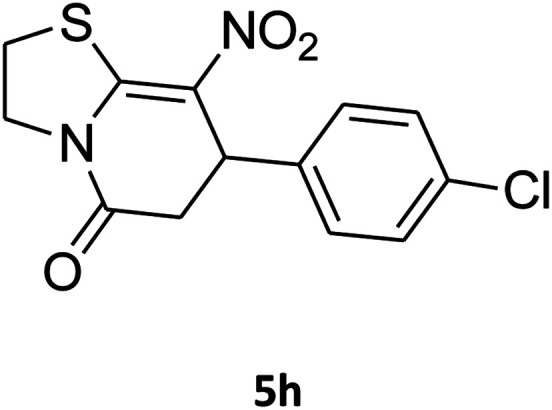	120	60
9	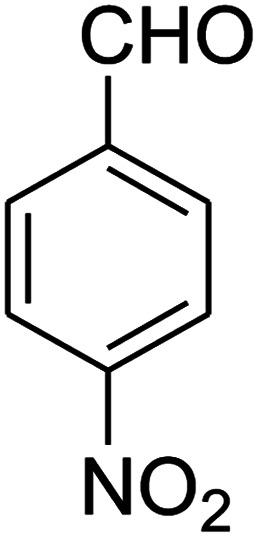	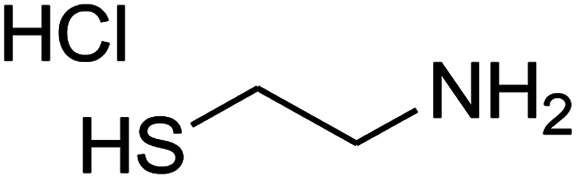	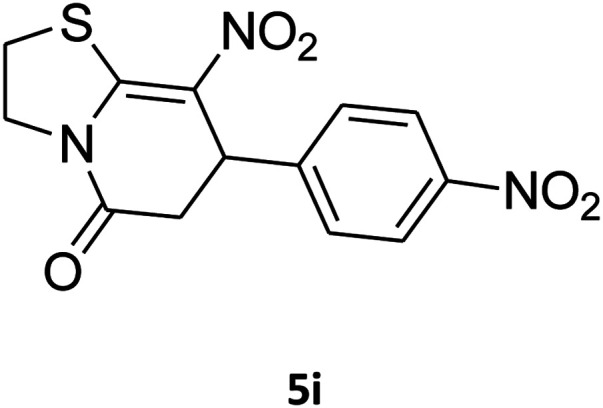	150	57
10	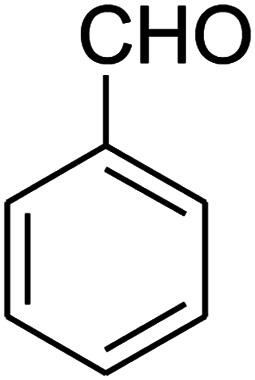	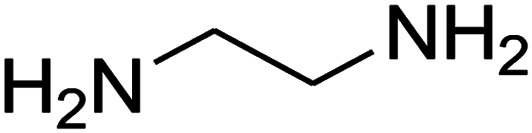	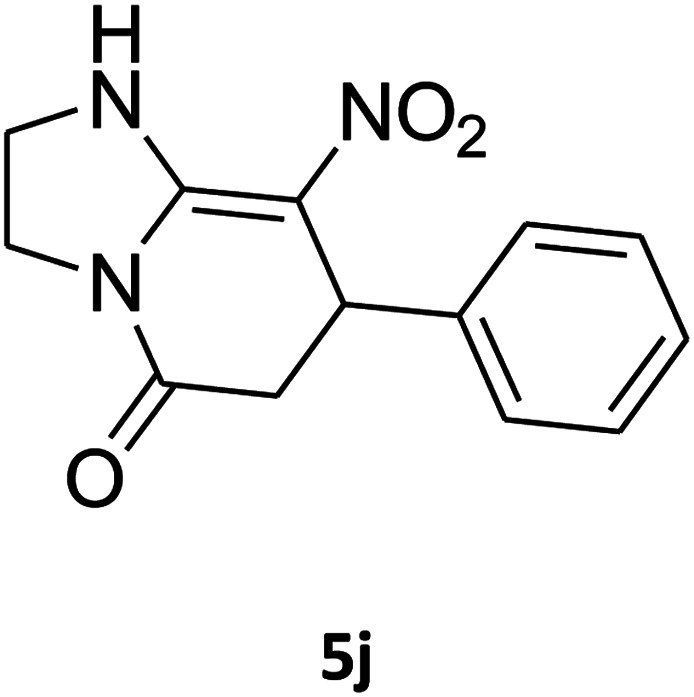	25	76
11	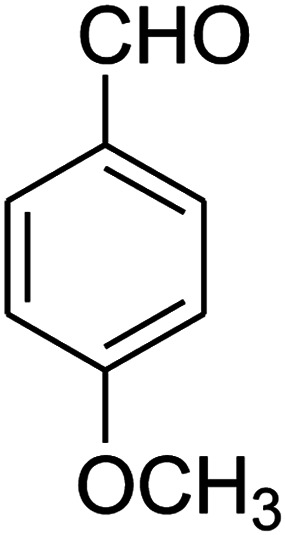	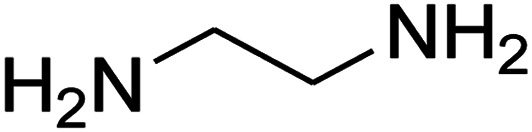	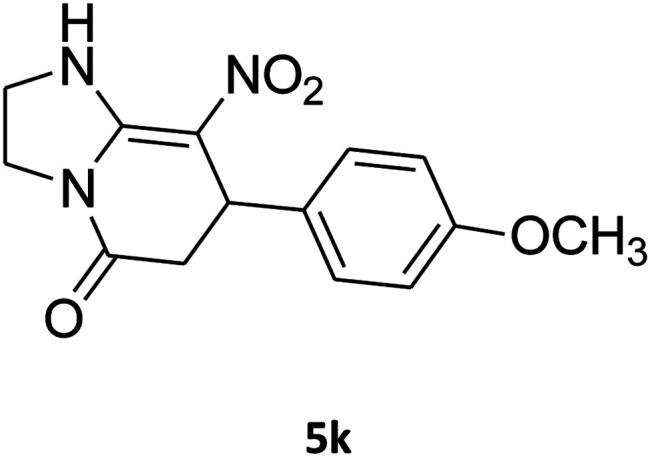	15	70
12	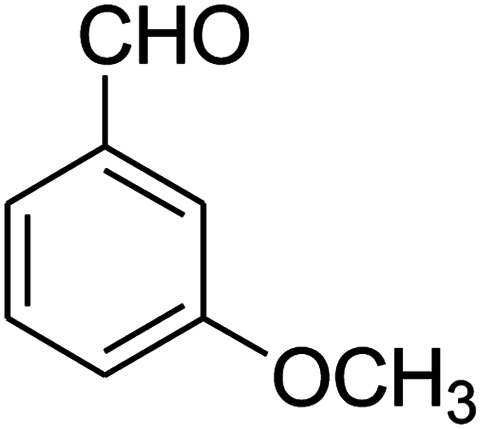	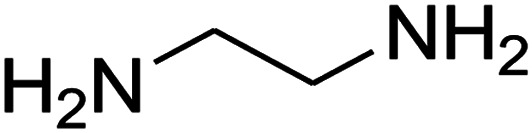	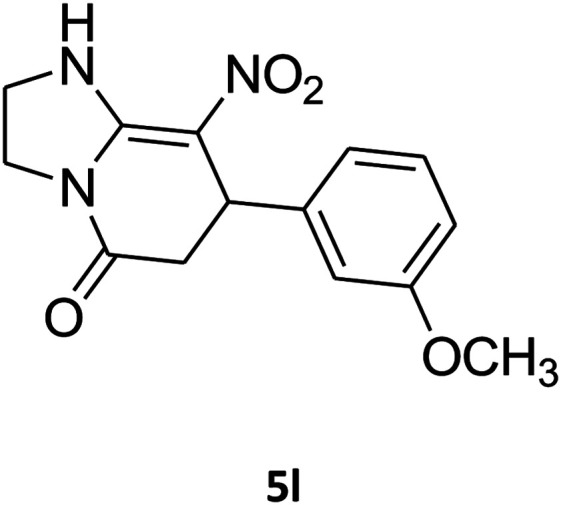	25	74
13	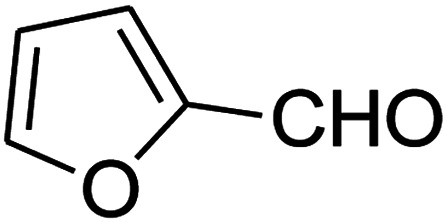	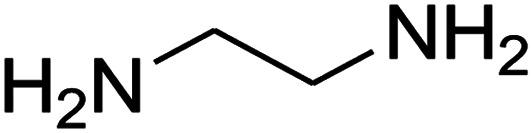	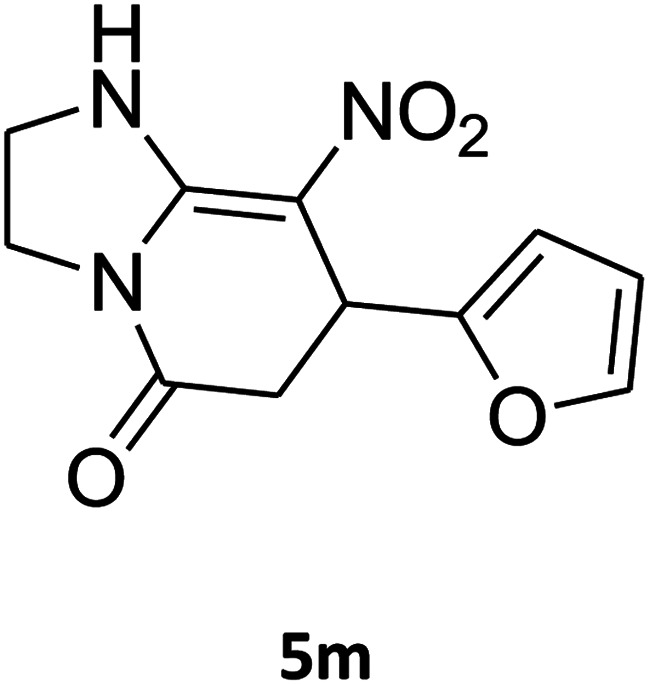	150	57
14	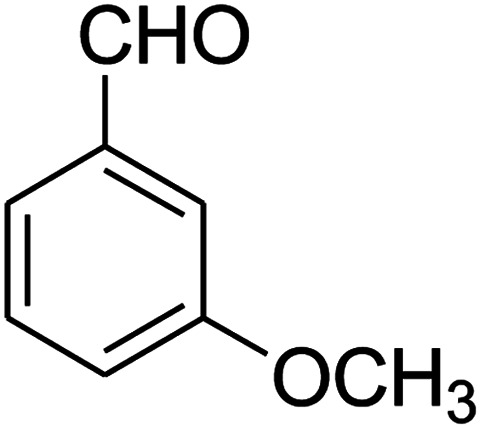	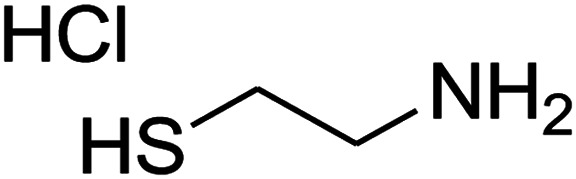	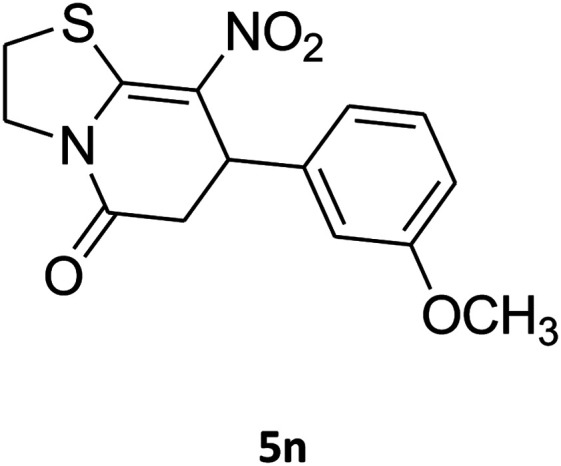	180	50
15	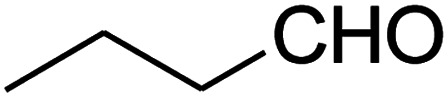	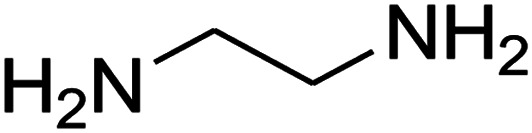	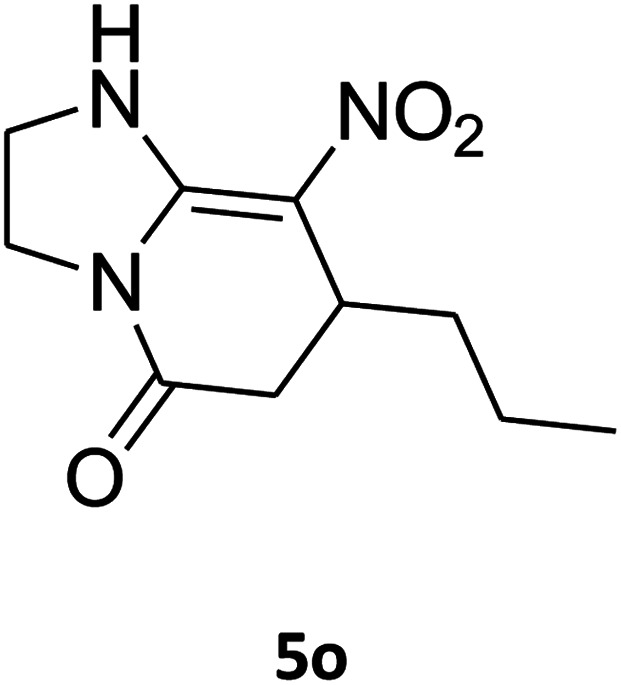	180	Trace
16^b^	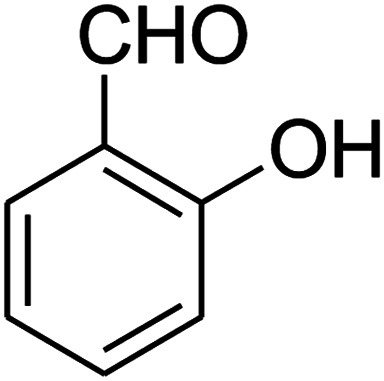	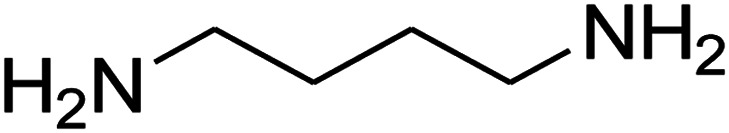	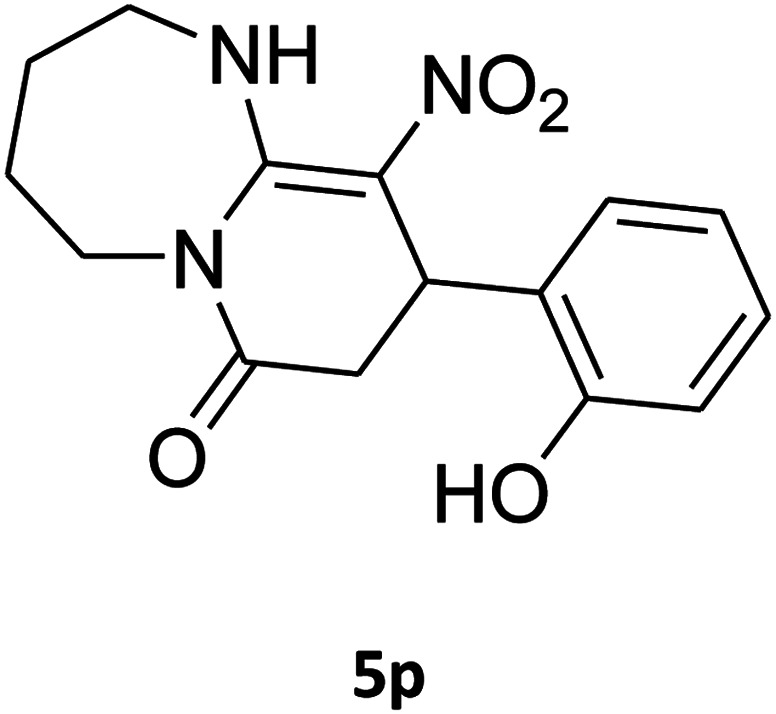	180	67
17	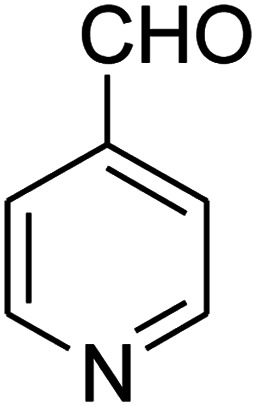	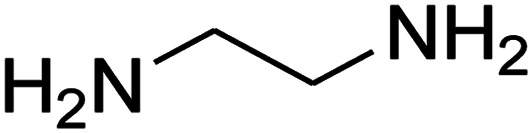	—	—	—
18	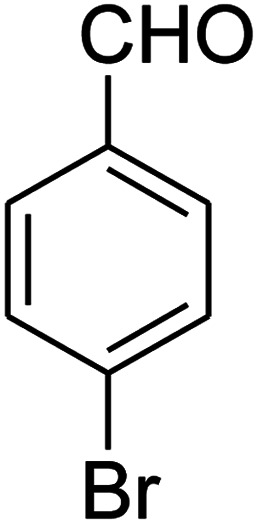	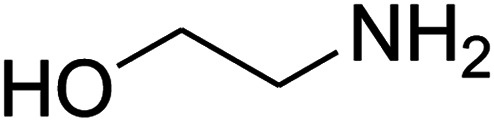	—	—	—
19	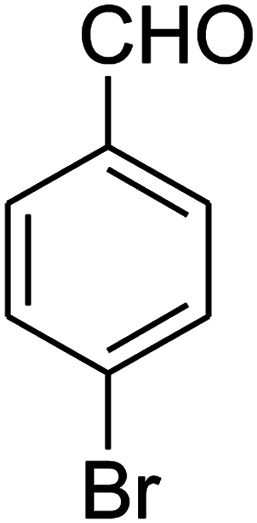	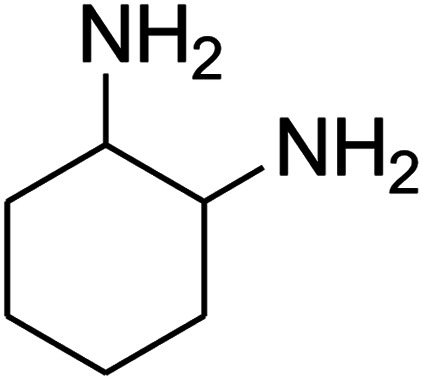	—	—	—
20	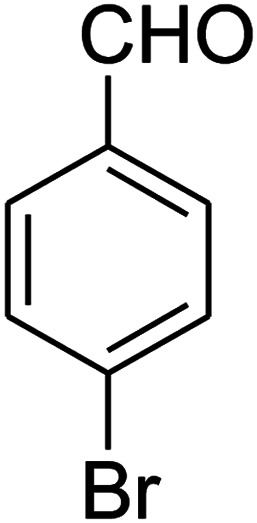	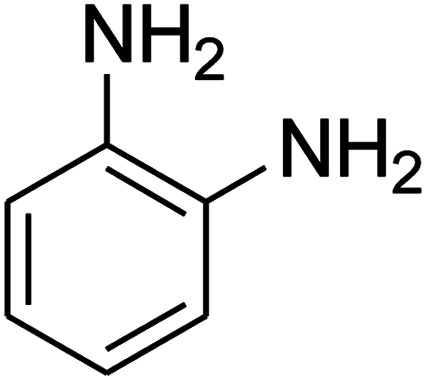	—	—	—

a Amine (1 mmol), 1,1-bis(methylthio)-2-nitroethene (1 mmol), aromatic aldehyde (1 mmol) and 2,2-dimethyl-1,3-dioxane-4,6-dione (Meldrum's acid) (1 mmol) were used.

b Ref. 38.

The structures of products were fully characterized using their infrared (IR), mass spectrometry, proton hydrogen-1 nuclear magnetic resonance (^1^H NMR), carbon-13 nuclear magnetic resonance (^13^C NMR) spectra. For example, the mass spectrum of 5a displayed the molecular ion peak at *m*/*z* 293 with frequency of 53% which was in accordance with the proposed structure. The IR spectrum of this structure indicated absorption bands due to the NH stretching (3231 cm^−1^) as well as bands at 2922, 1704, 1623, 1486 and 1337 cm^−1^ due to the CH, C=O, C=C and NO_2_ groups. The ^1^H NMR spectrum of 5a showed doublet of doublet for the CH_2_ group (*δ* 2.55, 3.21 ppm, ^2^*J*_HH_ = 15.6 Hz, ^3^*J*_HH_ = 7.8 Hz), multiplets for CH_2_NH and CH_2_N groups (*δ* 3.69–3.97 ppm), doublet for CH group (*δ* 4.52 ppm, ^3^*J*_HH_ = 7.5 Hz), two doublets for the aromatic region (*δ* 7.16, 7.32 ppm, ^3^*J*_HH_ = 8.4 Hz) and one singlet for NH group (*δ* 9.74 ppm). The ^1^H-decoupled ^13^C NMR spectrum of 5a showed 11 distinct resonances. There are three signals for CH and CH_2_ groups (*δ* 37.0, 43.0, 43.9 ppm), and signals at 106.3 and 167.6 ppm, which were assigned C–NO_2_ and C=O groups, respectively. The ^1^H and ^13^C NMR spectra of 5b–p are similar to those of 5a except for the aryl and diamine moieties, which exhibited characteristic signals with appropriate chemical shifts (see the ESI†).

**Scheme 3:**
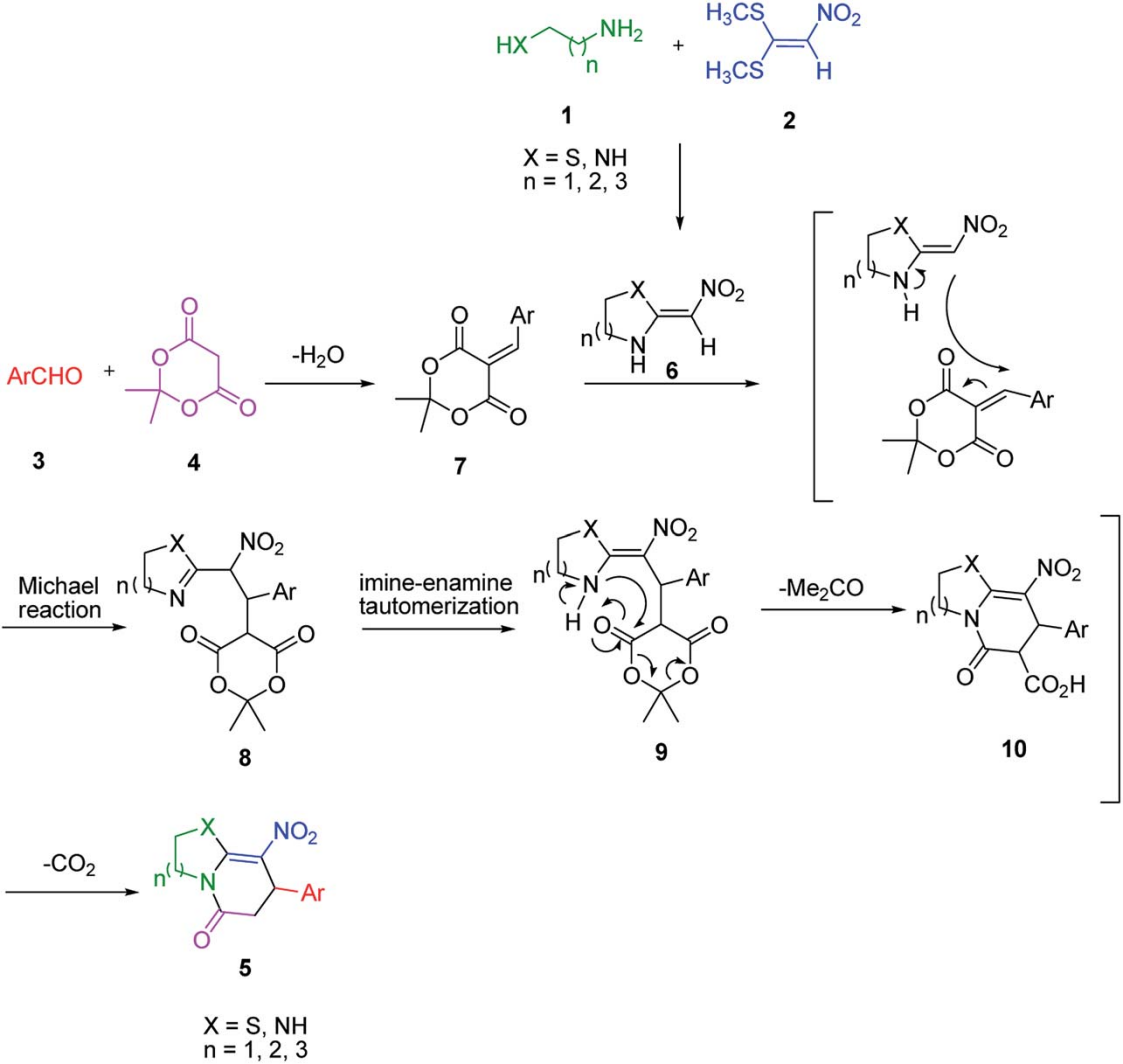
Plausible mechanism for the formation of product 5.

An acceptable reaction mechanism for this one-pot, multi-component reaction is designated in Scheme 3. The one-pot protocol started with the reaction of various diamines or cysteamine hydrochloride 1 and 1,1-bis(methylthio)-2-nitroethene 2 to afford enamine 6. Also condensation reaction between 2,2-dimethyl-1,3-dioxane-4,6-dione (Meldrum's acid) and the aromatic aldehydes gave adduct 7, which then reacted with enamine 6 to form intermediate 8. Compound 10 was formed from 8 through imine–enamine tautomerism and cyclocondensation. Finally, decarboxylation of 10 yielded the product 5.

## Experimental

### General

The various diamines, cysteamine hydrochloride, 1,1-bis(methylthio)-2-nitroethene, aldehydes, 2,2-dimethyl-1,3-dioxane-4,6-dione (Meldrum's acid) and solvents were purchased from Sigma-Aldrich chemical company and were used as received without further purification. Melting points were determined with an electrothermal 9100 apparatus. Infrared (IR) spectra were recorded on a Bruker Tensor 27 spectrometer. Nuclear magnetic resonance (NMR) spectra were obtained on a Bruker DRX-300 Avance instrument (300 MHz for ^1^H and 75.4 MHz for ^13^C) with DMSO as solvent. Chemical shifts are expressed in parts per million (ppm), and coupling constant (*J*) are reported in hertz (Hz). Elemental analyses for C, H and N were performed using a PerkinElmer 2004 series [II] CHN elemental analyzer. Mass spectra were recorded with an Agilent 5975C VL MSD with Triple-Axis Detector operating at an ionization potential of 70 eV.

### General procedure for the synthesis of product 5

#### Synthesis of imidazopyridinone and pyridopyrimidinone derivatives

The mixtures of various diamines (1 mmol), 1,1-bis(methylthio)-2-nitroethene (0.165 g, 1 mmol) and 10 mL EtOH/H_2_O (2 : 1) at 72 °C in a 50 mL flask was stirred for 6 h. After completion of the reaction (monitored by thin-layer chromatography, hexane/ethyl acetate 1 : 1), aromatic aldehyde (1 mmol), 2,2-dimethyl-1,3-dioxane-4,6-dione (Meldrum's acid) (0.144 g, 1 mmol) were added to the reaction mixture, and it was stirred under optimized condition for the time given in Table 4. Then, the reaction mixture was cooled to room temperature and filtered to give the crude product. The solid was washed with ethanol to give product in good yields.

#### Synthesis of thiazolopyridinone derivatives

The mixtures of cysteamine hydrochloride (0.113 g, 1 mmol), 1,1-bis(methylthio)-2-nitroethene (0.165 g, 1 mmol), 10 mL EtOH/H_2_O (2 : 1), and Et_3_N (140 μL, 1 mmol) at 72 °C in a 50 mL flask was stirred for 5 h. After completion of the reaction (monitored by thin-layer chromatography, hexane/ethyl acetate 1 : 1), aromatic aldehyde (1 mmol), 2,2-dimethyl-1,3-dioxane-4,6-dione (Meldrum's acid) (0.144 g, 1 mmol) were added to the reaction mixture, and it was stirred under optimized condition for the time given in Table 4. The reaction mixture was monitored by thin layer chromatography (hexane/ethyl acetate 1 : 1); after completion of the reaction, the reaction mixture was cooled to room temperature and the product (as colorless crystals) was filtered. The solid was washed with ethanol to give product in moderate yields.

##### 7-(4-Chlorophenyl)-2,3,6,7-tetrahydro-8-nitroimidazo[1,2-*a*]pyridin-5(1*H*)-one (5a)

White solid; yield: 0.257 g (88%); mp 299–301 °C. ^1^H NMR (300 MHz, DMSO-*d*_6_): *δ* 2.55 (d, ^2^*J*_HH_ = 15.6 Hz, 1H, CH_2_), 3.21 (dd, ^2^*J*_HH_ = 15.6 Hz, ^3^*J*_HH_ = 7.8 Hz, 1H, CH_2_), 3.69–3.97 (m, 4H, CH_2_NH and CH_2_N), 4.52 (d, ^3^*J*_HH_ = 7.5 Hz, 1H, CH), 7.16 (d, ^3^*J*_HH_ = 8.4 Hz, 2H, Ar), 7.32 (d, ^3^*J*_HH_ = 8.4 Hz, 2H, Ar), 9.74 (s, 1H, NH). ^13^C NMR (75.4 MHz, DMSO-*d*_6_): *δ* 37.0, 43.0, 43.9, 106.3, 128.8, 129.0, 131.9, 141.9, 153.0, 167.6. IR (KBr) (*ν*_max_/cm^−1^): 3231 (NH), 2922 (CH), 1704 (C=O), 1623 (C=C), 1486 and 1337 (NO_2_), 1214 (C–N). MS (EI, 70 eV): *m*/*z* (%) = 293 (M^+^, 53), 276 (42), 240 (100), 218 (23), 182 (59), 151 (35), 136 (45), 70 (18), 42 (14). Anal. calc. for C_13_H_12_ClN_3_O_3_ (293.71): C, 53.16; H, 4.12; N, 14.31. Found: C, 53.01; H, 4.51; N, 14.03.

##### 7-(4-Bromophenyl)-2,3,6,7-tetrahydro-8-nitroimidazo[1,2-*a*]pyridin-5(1*H*)-one (5b)

White solid; yield: 0.294 g (87%); mp 305–307 °C. ^1^H NMR (300 MHz, DMSO-*d*_6_): *δ* 2.55 (d, ^2^*J*_HH_ = 16.6 Hz, 1H, CH_2_), 3.21 (dd, ^2^*J*_HH_ = 16.6 Hz, ^3^*J*_HH_ = 7.8 Hz, 1H, CH_2_), 3.69–3.97 (m, 4H, CH_2_NH and CH_2_N), 4.51 (d, ^3^*J*_HH_ = 7.8 Hz, 1H, CH), 7.10 (d, ^3^*J*_HH_ = 7.2 Hz, 2H, Ar), 7.45 (d, ^3^*J*_HH_ = 7.2 Hz, 2H, Ar), 9.73 (s, 1H, NH). ^13^C NMR (75.4 MHz, DMSO-*d*_6_): *δ* 37.1, 43.1, 44.0, 106.3, 120.3, 129.1, 131.9, 142.3, 153.0, 167.6. IR (KBr) (*ν*_max_/cm^−1^): 3224 (NH), 2921 (CH), 1699 (C=O), 1623 (C=C), 1486 and 1337 (NO_2_), 1215 (C–N). MS (EI, 70 eV): *m*/*z* (%) = 337 (M^+^-1, 25), 320 (22), 291 (58), 240 (100), 212 (12), 182 (51), 136 (29), 116 (33), 70 (49), 42 (22). Anal. calc. for C_13_H_12_BrN_3_O_3_ (338.16): C, 46.17; H, 3.58; N, 12.43. Found: C, 46.52; H, 3.21; N, 12.12.

##### 2,3,6,7-Tetrahydro-8-nitro-7-(4-nitrophenyl)imidazo[1,2-*a*]pyridin-5(1*H*)-one (5c)

Yellow solid; yield: 0.270 g (89%); mp 264–266 °C. ^1^H NMR (300 MHz, DMSO-*d*_6_): *δ* 2.59 (d, ^2^*J*_HH_ = 15.6 Hz, 1H, CH_2_), 3.26 (dd, ^2^*J*_HH_ = 15.6 Hz, ^3^*J*_HH_ = 8.1 Hz, 1H, CH_2_), 3.71–3.99 (m, 4H, CH_2_NH and CH_2_N), 4.68 (d, ^3^*J*_HH_ = 7.5 Hz, 1H, CH), 7.44 (d, ^3^*J*_HH_ = 8.7 Hz, 2H, Ar), 8.12 (d, ^3^*J*_HH_ = 8.7 Hz, 2H, Ar), 9.79 (s, 1H, NH). ^13^C NMR (75.4 MHz, DMSO-*d*_6_): *δ* 37.6, 43.1, 44.0, 105.8, 124.3, 128.3, 146.9, 150.8, 153.0, 167.2. IR (KBr) (*ν*_max_/cm^−1^): 3227 (NH), 2923 (CH), 1712 (C=O), 1628 (C=C), 1520 and 1345 (NO_2_), 1219 (C–N). Anal. calc. for C_13_H_12_N_4_O_5_ (304.26): C, 51.32; H, 3.98; N, 18.41. Found: C, 51.56; H, 3.64; N, 18.16.

##### 7-(3-Fluorophenyl)-2,3,6,7-tetrahydro-8-nitroimidazo[1,2-*a*]pyridin-5(1*H*)-one (5d)

White solid; yield: 0.207 g (75%); mp 253–255 °C. ^1^H NMR (300 MHz, DMSO-*d*_6_): *δ* 2.59 (d, ^2^*J*_HH_ = 16.6 Hz, 1H, CH_2_), 3.20 (m, ^2^*J*_HH_ = 16.6 Hz, ^3^*J*_HH_ = 8.1 Hz, 1H, CH_2_), 3.70–3.94 (m, 4H, CH_2_NH and CH_2_N), 4.55 (d, ^3^*J*_HH_ = 7.5 Hz, 1H, CH), 6.96–7.06 (m, 3H, Ar), 7.28–7.35 (m, 1H, Ar), 9.75 (s, 1H, NH). ^13^C NMR (75.4 MHz, DMSO-*d*_6_): *δ* 37.4, 43.1, 44.0, 106.2, 113.8 (d, ^2^*J*_CF_ = 21 Hz), 114.2 (d, ^2^*J*_CF_ = 21 Hz), 122.8, 131.1, 145.9, 153.1, 162.8 (d, ^1^*J*_CF_ = 244 Hz), 167.6. Anal. calc. for C_13_H_12_FN_3_O_3_ (277.25): C, 56.32; H, 4.36; N, 15.16. Found: C, 56.70; H, 4.06, N, 15.38.

##### 8-(4-Chlorophenyl)-1,2,3,4,7,8-hexahydro-9-nitropyrido[1,2-*a*]pyrimidin-6-one (5e)

White solid; yield: 0.260 g (85%); mp 282–284 °C. ^1^H NMR (300 MHz, DMSO-*d*_6_): *δ* 1.89–1.98 (m, 2H, CH_2_), 2.65 (d, ^2^*J*_HH_ = 15.0 Hz, 1H, CH_2_), 3.21 (dd, ^2^*J*_HH_ = 15.0 Hz, ^3^*J*_HH_ = 7.5 Hz, 1H, CH_2_), 3.41–3.89 (m, 4H, CH_2_NH and CH_2_N), 4.59 (d, ^3^*J*_HH_ = 6.6 Hz, 1H, CH), 7.15 (d, ^3^*J*_HH_ = 8.4 Hz, 2H, Ar), 7.31 (d, ^3^*J*_HH_ = 7.2 Hz, 2H, Ar), 11.55 (s, 1H, NH). ^13^C NMR (75.4 MHz, DMSO-*d*_6_): *δ* 19.5, 36.0, 38.6, 108.6, 128.8, 129.0, 131.9, 140.9, 152.9, 168.6. IR (KBr) (*ν*_max_/cm^−1^): 3089 (NH), 2927 (CH), 1710 (C=O), 1618 (C=C), 1502 and 1330 (NO_2_), 1232 (C–N). MS (EI, 70 eV): *m*/*z* (%) = 307 (M^+^, 23), 290 (17), 261 (100), 196 (20), 178 (13), 151 (37), 115 (19), 84 (31), 56 (29). Anal. calc. for C_14_H_14_ClN_3_O_3_ (307.73): C, 54.64; H, 4.59; N, 13.65. Found: C, 54.26; H, 4.90; N, 13.49.

##### 7-(4-Bromophenyl)-2,3,6,7-tetrahydro-8-nitrothiazolo[3,2-*a*]pyridin-5-one (5f)

Yellow solid; yield: 0.230 g (65%); mp 204–206 °C. ^1^H NMR (300 MHz, DMSO-*d*_6_): *δ* 2.60 (d, ^2^*J*_HH_ = 16.5 Hz, 1H, CH_2_), 3.17–3.39 (m, 3H, CH_2_ and CH_2_S), 3.96–4.06 (m, 1H, CH_2_N), 4.32–4.39 (m, 1H, CH_2_N), 4.64 (d, ^3^*J*_HH_ = 8.4 Hz, 1H, CH), 7.14 (d, ^3^*J*_HH_ = 8.4 Hz, 2H, Ar), 7.36 (d, ^3^*J*_HH_ = 8.4 Hz, 2H, Ar). ^13^C NMR (75.4 MHz, DMSO-*d*_6_): *δ* 29.1, 37.7, 49.4, 120.9, 124.8, 129.0, 132.3, 140.3, 159.9, 166.6. IR (KBr) (*ν*_max_/cm^−1^): 2918 (CH), 1699 (C=O), 1573 (C=C), 1420 and 1350 (NO_2_), 1223 (C–N). MS (EI, 70 eV): *m*/*z* (%) = 355 (M^+^, 58), 339 (100), 309 (40), 281 (17), 213 (16), 182 (22), 140 (18), 102 (21), 60 (15). Anal. calc. for C_13_H_11_BrN_2_O_3_S (355.21): C, 43.96; H, 3.12; N, 7.89. Found: C, 43.56; H, 3.33; N, 8.26.

##### 2,3,6,7-Tetrahydro-8-nitro-7-phenylthiazolo[3,2-*a*]pyridin-5-one (5g)

Yellow solid; yield: 0.146 g (53%); mp 175–177 °C. ^1^H NMR (300 MHz, DMSO-*d*_6_): *δ* 2.66 (d, ^2^*J*_HH_ = 16.2 Hz, 1H, CH_2_), 3.25–3.40 (m, 3H, CH_2_ and CH_2_S), 3.95–4.05 (m, 1H, CH_2_N), 4.33–4.41 (m, 1H, CH_2_N), 4.63 (d, ^3^*J*_HH_ = 7.5 Hz, 1H, CH), 7.09–7.32 (m, 5H, Ar). ^13^C NMR (75.4 MHz, DMSO-*d*_6_): *δ* 29.0, 38.3, 49.4, 125.3, 126.7, 127.8, 129.5, 140.9, 159.6, 166.8.

##### 7-(4-Chlorophenyl)-2,3,6,7-tetrahydro-8-nitrothiazolo[3,2-*a*]pyridin-5-one (5h)

Yellow solid; yield: 0.186 g (60%); mp 128–130 °C. ^1^H NMR (300 MHz, DMSO-*d*_6_): *δ* 2.65 (d, ^2^*J*_HH_ = 16.2 Hz, 1H, CH_2_), 3.18–3.40 (m, 3H, CH_2_ and CH_2_S), 3.96–4.06 (m, 1H, CH_2_N), 4.32–4.40 (m, 1H, CH_2_N), 4.65 (d, ^3^*J*_HH_ = 7.5 Hz, 1H, CH), 7.14 (d, ^3^*J*_HH_ = 8.7 Hz, 2H, Ar), 7.35 (d, ^3^*J*_HH_ = 8.4 Hz, 2H, Ar). ^13^C NMR (75.4 MHz, DMSO-*d*_6_): *δ* 29.1, 37.7, 49.5, 124.9, 128.7, 129.4, 132.4, 139.9, 159.9, 166.6. IR (KBr) (*ν*_max_/cm^−1^): 2918 (CH), 1703 (C=O), 1573 (C=C), 1507 and 1350 (NO_2_), 1246 (C–N).

##### 2,3,6,7-Tetrahydro-8-nitro-7-(4-nitrophenyl)thiazolo[3,2-*a*]pyridin-5-one (5i)

Yellow solid; yield: 0.182 g (57%); mp 145–147 °C. ^1^H NMR (300 MHz, DMSO-*d*_6_): *δ* 2.70 (d, ^2^*J*_HH_ = 16.8 Hz, 2H, CH_2_), 3.07–3.46 (m, 3H, CH_2_ and CH_2_S), 3.99–4.09 (m, 1H, CH_2_N), 4.33–4.41 (m, 1H, CH_2_N), 4.81 (d, ^3^*J*_HH_ = 8.1 Hz, 1H, CH), 7.42 (d, ^3^*J*_HH_ = 8.7 Hz, 2H, Ar), 8.15 (d, ^3^*J*_HH_ = 8.7 Hz, 2H, Ar). ^13^C NMR (75.4 MHz, DMSO-*d*_6_): *δ* 29.1, 38.2, 49.5, 124.2, 124.6, 128.3, 147.2, 148.7, 160.4, 166.3. IR (KBr) (*ν*_max_/cm^−1^): 2919 (CH), 1709 (C=O), 1573 (C=C), 1522 and 1357 (NO_2_), 1226 (C–N). MS (EI, 70 eV): *m*/*z* (%) = 321 (M^+^, 36), 304 (100), 274 (45), 246 (12), 199 (8), 140 (6), 60 (11). Anal. calc. for C_13_H_11_N_3_O_5_S (321.04): C, 48.59; H, 3.45; N, 13.08. Found: C, 48.79; H, 3.82; N, 13.50.

##### 2,3,6,7-Tetrahydro-8-nitro-7-phenylimidazo[1,2-*a*]pyridin-5(1*H*)-one (5j)

White solid; yield: 0.196 g (76%); mp 266–268 °C. ^1^H NMR (300 MHz, DMSO-*d*_6_): *δ* 2.55 (d, ^2^*J*_HH_ = 16.2 Hz, 1H, CH_2_), 3.22 (dd, ^2^*J*_HH_ = 16.2 Hz, ^3^*J*_HH_ = 7.5 Hz, 1H, CH_2_), 3.73–3.96 (m, 4H, CH_2_NH and CH_2_N), 4.51 (d, ^3^*J*_HH_ = 7.2 Hz, 1H, CH), 7.02–7.29 (m, 5H, Ar), 9.73 (s, 1H, NH). ^13^C NMR (75.4 MHz, DMSO-*d*_6_): *δ* 37.6, 43.0, 43.9, 106.6, 126.8, 127.3, 129.1, 142.9, 153.1, 167.8.

##### 2,3,6,7-Tetrahydro-7-(4-methoxyphenyl)-8-nitroimidazo[1,2-*a*]pyridin-5(1*H*)-one (5k)

White solid; yield: 0.202 g (70%); mp 307–309 °C. ^1^H NMR (300 MHz, DMSO-*d*_6_): *δ* 2.55 (d, ^2^*J*_HH_ = 16.5 Hz, 1H, CH_2_), 3.14 (dd, ^2^*J*_HH_ = 16.5 Hz, ^3^*J*_HH_ = 7.8 Hz, 1H, CH_2_), 3.68 (s, 1H, OCH_3_), 3.72–3.97 (m, 4H, CH_2_NH and CH_2_N), 4.32 (d, ^3^*J*_HH_ = 7.5 Hz, 1H, CH), 6.81 (d, ^3^*J*_HH_ = 7.5 Hz, 2H, Ar), 7.03 (d, ^3^*J*_HH_ = 7.8 Hz, 2H, Ar), 9.69 (s, 1H, NH). ^13^C NMR (75.4 MHz, DMSO-*d*_6_): *δ* 36.7, 43.0, 43.9, 55.4, 107.0, 114.4, 127.8, 134.7, 153.0, 158.6, 167.9.

##### 2,3,6,7-Tetrahydro-7-(3-methoxyphenyl)-8-nitroimidazo[1,2-*a*]pyridin-5(1*H*)-one (5l)

White solid; yield: 0.213 g (74%); mp 213–215 °C. ^1^H NMR (300 MHz, DMSO-*d*_6_): *δ* 2.56 (d, ^2^*J*_HH_ = 16.5 Hz, 1H, CH_2_), 3.14 (dd, ^2^*J*_HH_ = 16.5 Hz, ^3^*J*_HH_ = 8.1 Hz, 1H, CH_2_), 3.69 (s, 1H, OCH_3_), 3.79–3.97 (m, 4H, CH_2_NH and CH_2_N), 4.49 (d, ^3^*J*_HH_ = 7.5 Hz, 1H, CH), 6.66 (s, 2H, Ar), 6.77 (d, ^3^*J*_HH_ = 7.2 Hz, 1H, Ar), 7.18 (t, ^3^*J*_HH_ = 8.1 Hz, 1H, Ar), 9.72 (s, 1H, NH). ^13^C NMR (75.4 MHz, DMSO-*d*_6_): *δ* 37.5, 43.0, 43.9, 55.3, 106.5, 112.2, 113.1, 118.6, 130.2, 144.5, 153.1, 159.9, 167.8.

##### 7-(Furan-2-yl)-2,3,6,7-tetrahydro-8-nitroimidazo[1,2-*a*]pyridin-5(1*H*)-one (5m)

White solid; yield: 0.141 g (57%); mp 241–243 °C. ^1^H NMR (300 MHz, DMSO-*d*_6_): *δ* 2.67 (d, ^2^*J*_HH_ = 16.5 Hz, 1H, CH_2_), 3.10 (dd, ^2^*J*_HH_ = 16.5 Hz, ^3^*J*_HH_ = 7.5 Hz, 1H, CH_2_), 3.70–3.97 (m, 4H, CH_2_NH and CH_2_N), 4.61 (d, ^3^*J*_HH_ = 6.9 Hz, 1H, CH), 6.04 (s, 1H, Ar), 6.31 (s, 1H, Ar), 7.50 (s, 1H, Ar), 9.63 (s, 1H, NH). ^13^C NMR (75.4 MHz, DMSO-*d*_6_): *δ* 32.0, 36.6, 43.0, 43.8, 104.9, 105.7, 110.8, 142.6, 152.7, 154.7, 167.7.

##### 2,3,6,7-Tetrahydro-7-(3-methoxyphenyl)-8-nitrothiazolo[3,2-*a*]pyridin-5-one (5n)

Yellow solid; yield: 0.153 g (50%); mp 130–132 °C. ^1^H NMR (300 MHz, DMSO-*d*_6_): *δ* 2.70 (d, ^2^*J*_HH_ = 16.5 Hz, 1H, CH_2_), 3.27–3.40 (m, 3H, CH_2_ and CH_2_S), 3.70 (s, 1H, OCH_3_), 3.93–4.03 (m, 1H, CH_2_N), 4.33–4.41 (m, 1H, CH_2_N), 4.61 (d, ^3^*J*_HH_ = 7.2 Hz, 1H, CH), 6.61–6.83 (m, 3H, Ar), 7.21 (t, ^3^*J*_HH_ = 7.8 Hz, 1H, Ar). ^13^C NMR (75.4 MHz, DMSO-*d*_6_): *δ* 29.0, 38.2, 49.4, 55.4, 112.8, 112.9, 118.3, 125.1, 130.6, 142.5, 159.6, 160.1, 166.8.

##### 2,3,6,7-Tetrahydro-8-nitro-7-propylimidazo[1,2-*a*]pyridin-5(1*H*)-one (5o)

White solid; mp 174–176 °C. ^1^H NMR (300 MHz, DMSO-*d*_6_): *δ* 0.83 (t, 3H, CH_3_), 1.15–1.45 (m, 4H, 2CH_2_), 2.42 (d, ^2^*J*_HH_ = 16.8 Hz, 1H, CH_2_), 2.77 (dd, ^2^*J*_HH_ = 16.8 Hz, ^3^*J*_HH_ = 7.2 Hz, 1H, CH_2_), 3.62–3.93 (m, 5H, CH and CH_2_NH and CH_2_N), 9.54 (s, 1H, NH).

## Conclusion

We have reported a simple and clean synthesis of imidazopyridinone, pyridopyrimidinone, and thiazolopyridinone derivatives. We were able to optimize the reaction conditions using RSM in conjunction with a central composite design. Further experimentation revealed the optimal values of the water content of aqueous ethanol (33%), and reaction temperature (72 °C). Also good yield of product in mild conditions show that the experimental results are in good agreement with the predicted values, and the model successfully can be used to predict the synthesis of such heterocyclic compounds.

## Conflicts of interest

The authors declare no competing financial interest.

## Supplementary Material

RA-009-C9RA06054E-s001
