# Supplementary material for: Simple synthesis of new imidazopyridinone, pyridopyrimidinone, and thiazolopyridinone derivatives and optimization of reaction parameters using response surface methodology
Source: RSC Adv. 2019 Sep 25;9(52):30479–88. doi: 10.1039/c9ra06054e (PMC9072147; doi:10.1039/c9ra06054e)
Supplement: RA-009-C9RA06054E-s001 [file RA-009-C9RA06054E-s001.pdf]

Electronic Supplementary Material (ESI) for RSC Advances.
This journal is © The Royal Society of Chemistry 2019

Supporting Information

Simple synthesis of new imidazopyridinone, pyridopyrimidinone, and thiazolopyridinone derivatives and optimization of reaction parameters using response surface methodology

Fahimeh Sadat Hosseini[a], Mohammad Bayat*[a]

[a] *Chemistry Department, Imam Khomeini International University, Qazvin, Iran*

Tel: +98 (28)33780040; Fax: +98 (28)33780040; E-mail: bayat_mo@yahoo.com; m.bayat@sci.ikiu.ac.ir

The Table of Contents

Title	Page
Title, author's name, address and table of contents	1
Experimental Section	1-2
Figure 1. Structure of all products **5**	3
^{1}H and ^{13}C NMR and IR and Mass spectrums of **5a**	4-7
^{1}H and ^{13}C NMR and IR and Mass spectrums of **5b**	8-11
^{1}H and ^{13}C NMR and IR spectrums of **5c**	12-14
^{1}H and ^{13}C NMR spectrums of **5d**	15-16
^{1}H and ^{13}C NMR and IR and Mass spectrums of **5e**	17-20
^{1}H and ^{13}C NMR and IR and Mass spectrums of **5f**	21-24
^{1}H and ^{13}C NMR spectrums of **5g**	25-26
^{1}H and ^{13}C NMR and IR spectrums of **5h**	27-29
^{1}H and ^{13}C NMR and IR and Mass spectrums of **5i**	30-33
^{1}H and ^{13}C NMR spectrums of **5j**	34-35
^{1}H and ^{13}C NMR spectrums of **5k**	36-37
^{1}H and ^{13}C NMR spectrums of **5l**	38-39
^{1}H and ^{13}C NMR spectrums of **5m**	40-41
^{1}H and ^{13}C NMR spectrums of **5n**	42
^{1}H NMR spectrum of **5o**	43-44

Experimental Section

General

The various diamines, cysteamine hydrochloride, 1,1-bis(methylthio)-2-nitroethene, various aldehydes, 2,2-dimethyl-1,3-dioxane-4,6-dione (Meldrum′s acid) and solvents were purchased from Sigma-Aldrich chemical company and were used as received without further purification. Melting points were determined with an electrothermal 9100 apparatus. Infrared (IR) spectra were recorded on a Bruker Tensor 27 spectrometer. Nuclear magnetic resonance (NMR) spectra were obtained on a Bruker DRX-300 Avance instrument (300 MHz for ^{1}H and 75.4 MHz for ^{13}C) with DMSO as solvent. Chemical shifts are expressed in parts per million (ppm), and coupling constant (*J*) are reported in hertz (Hz). Elemental analyses for C, H and N were performed using a PerkinElmer 2004 series [II] CHN elemental analyzer. Mass spectra were recorded with an Agilent 5975C VL MSD with Triple-Axis Detector operating at an ionization potential of 70 eV.

General procedure for the synthesis of product 5.

Synthesis of imidazopyridinone and pyridopyrimidinone derivatives: The mixtures of various diamines (1 mmol), 1,1-bis(methylthio)-2-nitroethene (0.165 g, 1 mmol) and 10 mL EtOH/H_2O (2:1) at 72 °C in a 50 mL flask was stirred for 6 h. After completion of the reaction (monitored by thin-layer chromatography, hexane/ethyl acetate 1:1), aromatic aldehyde (1 mmol), 2,2-dimethyl-1,3-dioxane-4,6-dione (Meldrum′s acid) (0.144 g, 1 mmol) were added to the reaction mixture, and it was stirred under optimized condition for the time given in Table 4. Then, the reaction mixture was cooled to room temperature and filtered to give the crude product. The solid was washed with ethanol to give product in good yields.

Synthesis of thiazolopyridinone derivatives: The mixtures of cysteamine hydrochloride (0.113 g, 1 mmol), 1,1-bis(methylthio)-2-nitroethene (0.165 g, 1 mmol), 10 mL EtOH/H_2O (2:1), and Et_3N (140 µL, 1 mmol) at 72 °C in a 50 mL flask was stirred for 5 h. After completion of the reaction (monitored by thin-layer chromatography, hexane/ethyl acetate 1:1), aromatic aldehyde (1 mmol), 2,2-dimethyl-1,3-dioxane-4,6-dione (Meldrum′s acid) (0.144 g, 1 mmol) were added to the reaction mixture, and it was stirred under optimized condition for the time given in Table 4. The reaction mixture was monitored by thin layer chromatography (hexane/ethyl acetate 1:1); after completion of the reaction, the reaction mixture was cooled to room temperature and the product (as colorless crystal) was filtered. The solid was washed with ethanol to give product in moderate yields.

The structures all of the products **5a-o** (figure 1) were deduced from their IR, Mass, ^{1}H NMR, and ^{13}C NMR spectra.

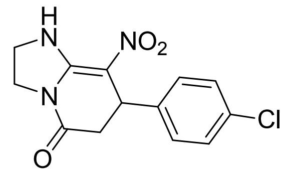

5a

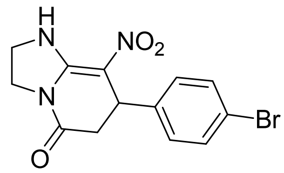

5b

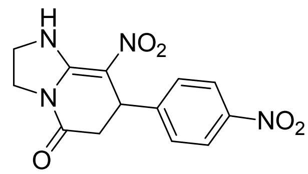

5c

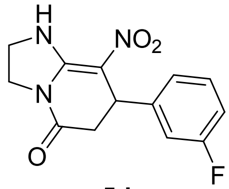

5d

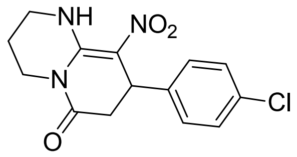

5e

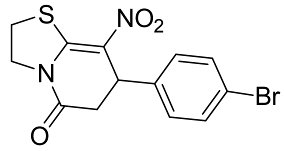

5f

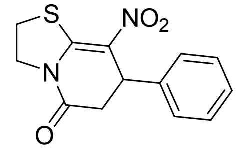

5g

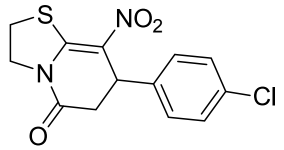

5h

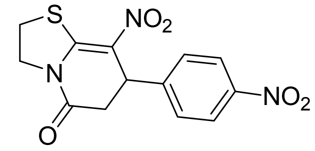

5i

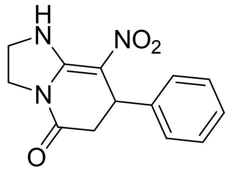

5j

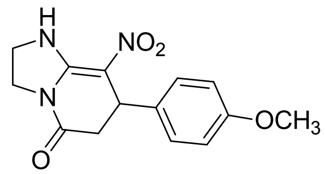

5k

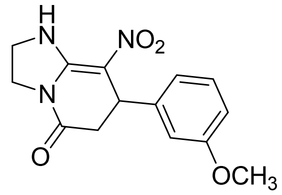

5l

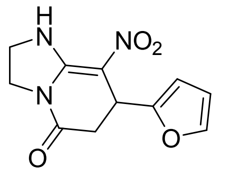

5m

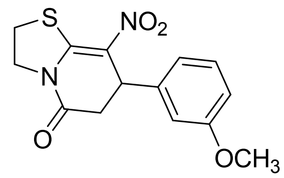

5n

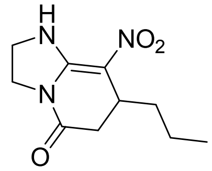

5o

Figure 1. Structure of all products **5.**

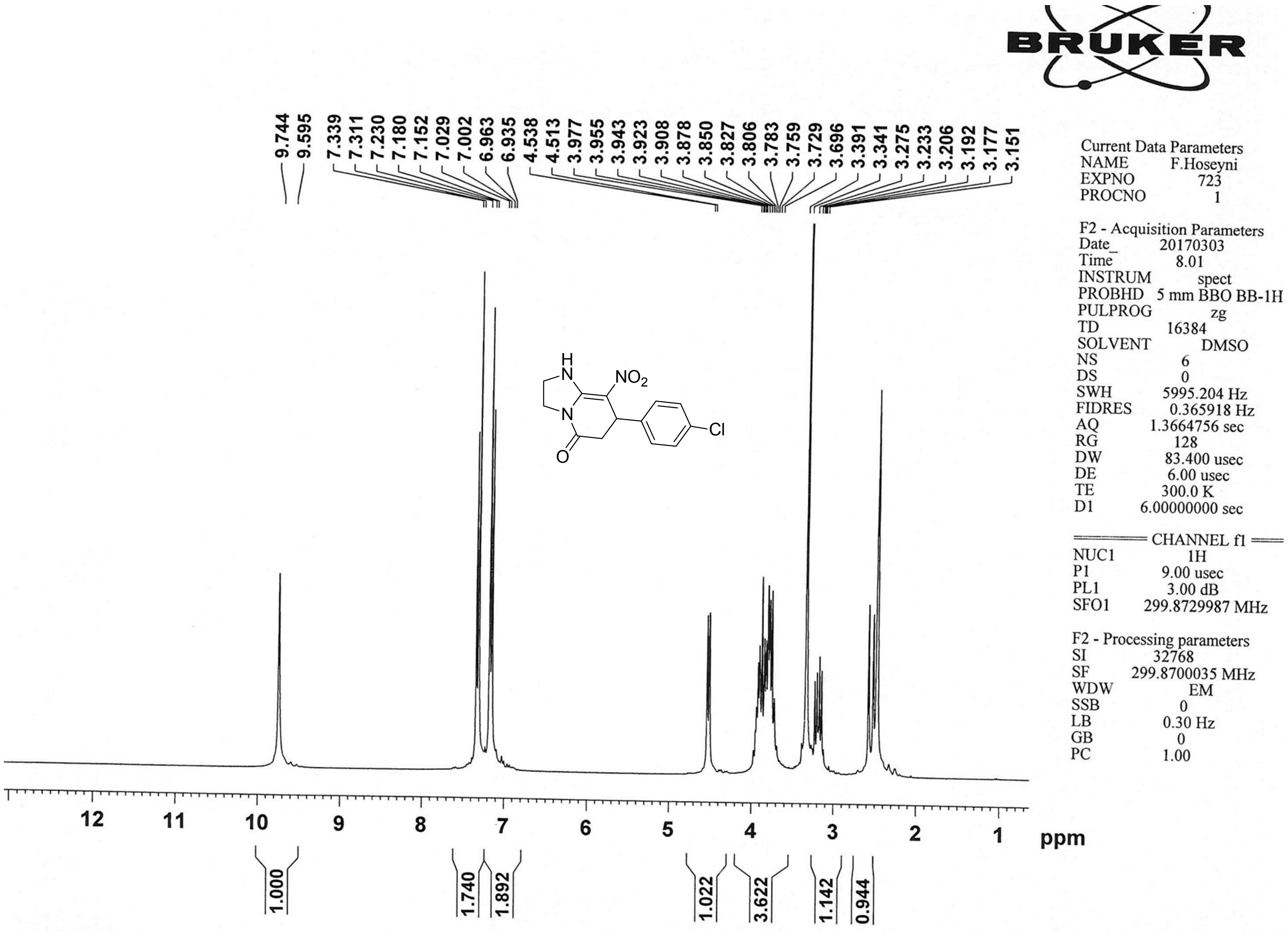
BRUKER
9.744
9.595
7.339
7.311
7.230
7.180
7.152
7.029
7.002
6.963
6.935
4.538
4.513
3.977
3.955
3.943
3.923
3.908
3.878
3.850
3.827
3.806
3.783
3.759
3.729
3.696
3.391
3.341
3.275
3.233
3.206
3.192
3.177
3.151
Current Data Parameters
NAME F.Hoseyni
EXPNO 723
PROCNO 1
F2 - Acquisition Parameters
Date_ 20170303
Time 8.01
INSTRUM spect
PROBHD 5 mm BBO BB-1H
PULPROG zg
TD 16384
SOLVENT DMSO
NS 6
DS 0
SWH 5995.204 Hz
FIDRES 0.365918 Hz
AQ 1.3664756 sec
RG 128
DW 83.400 usec
DE 6.00 usec
TE 300.0 K
D1 6.00000000 sec
CHANNEL f1
NUC1 1H
P1 9.00 usec
PL1 3.00 dB
SFO1 299.8729987 MHz
F2 - Processing parameters
SI 32768
SF 299.8700035 MHz
WDW EM
SSB 0
LB 0.30 Hz
GB 0
PC 1.00
H
N
NO2
N
Cl
O
12
11
10
9
8
7
6
5
4
3
2
1
ppm
1.000
1.740
1.892
1.022
3.622
1.142
0.944

^{1}H NMR of 5a

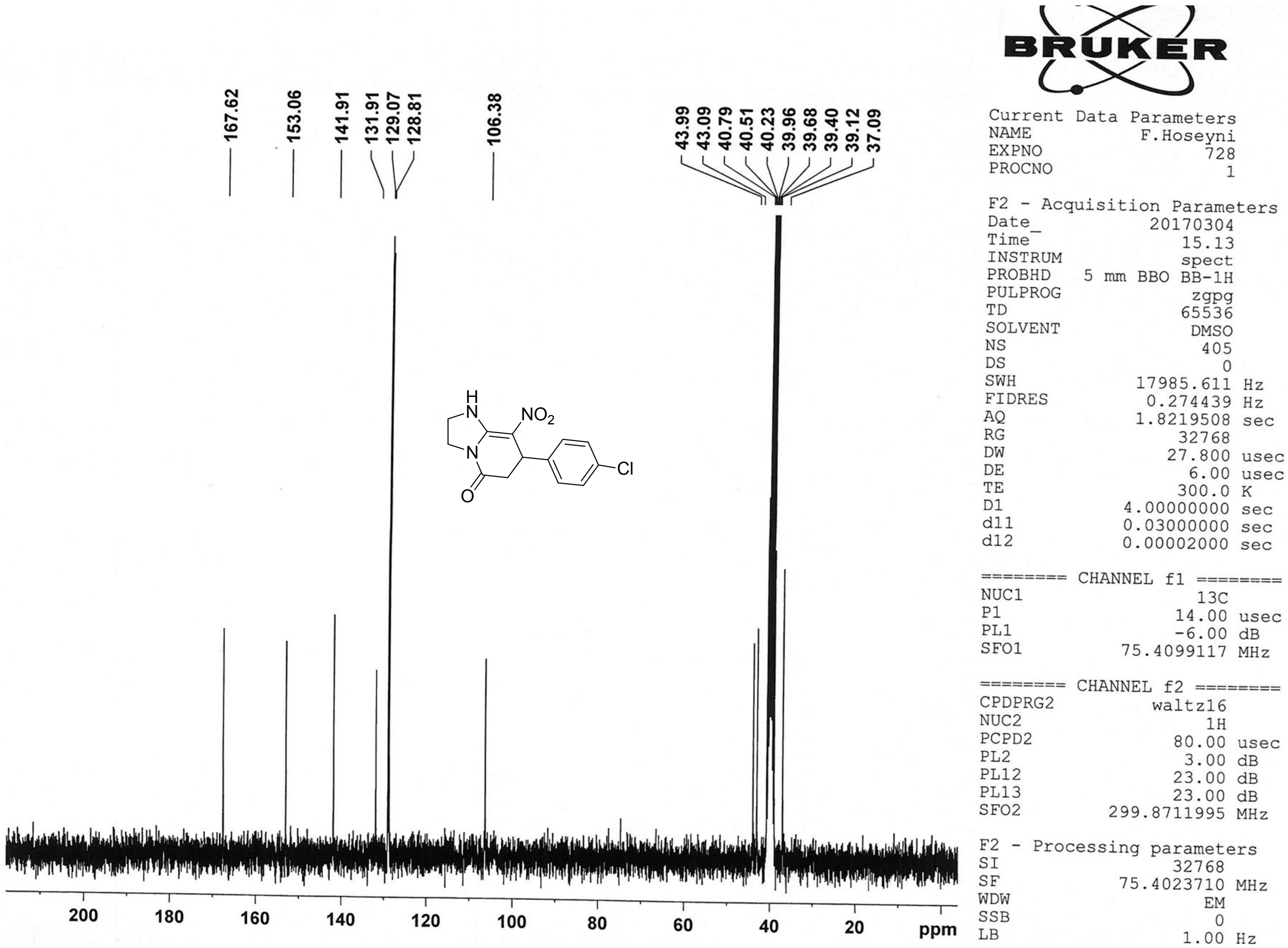
167.62
153.06
141.91
131.91
129.07
128.81
106.38
43.99
43.09
40.79
40.51
40.23
39.96
39.68
39.40
39.12
37.09
H
N
NO2
N
Cl
O
200
180
160
140
120
100
80
60
40
20
ppm
BRUKER
Current Data Parameters
NAME F.Hoseyni
EXPNO 728
PROCNO 1
F2 - Acquisition Parameters
Date_ 20170304
Time 15.13
INSTRUM spect
PROBHD 5 mm BBO BB-1H
PULPROG zgpg
TD 65536
SOLVENT DMSO
NS 405
DS 0
SWH 17985.611 Hz
FIDRES 0.274439 Hz
AQ 1.8219508 sec
RG 32768
DW 27.800 usec
DE 6.00 usec
TE 300.0 K
D1 4.00000000 sec
d11 0.03000000 sec
d12 0.00002000 sec
======== CHANNEL f1 ========
NUC1 13C
P1 14.00 usec
PL1 -6.00 dB
SFO1 75.4099117 MHz
======== CHANNEL f2 ========
CPDPRG2 waltz16
NUC2 1H
PCPD2 80.00 usec
PL2 3.00 dB
PL12 23.00 dB
PL13 23.00 dB
SFO2 299.8711995 MHz
F2 - Processing parameters
SI 32768
SF 75.4023710 MHz
WDW EM
SSB 0
LB 1.00 Hz

^{13}C NMR of 5a

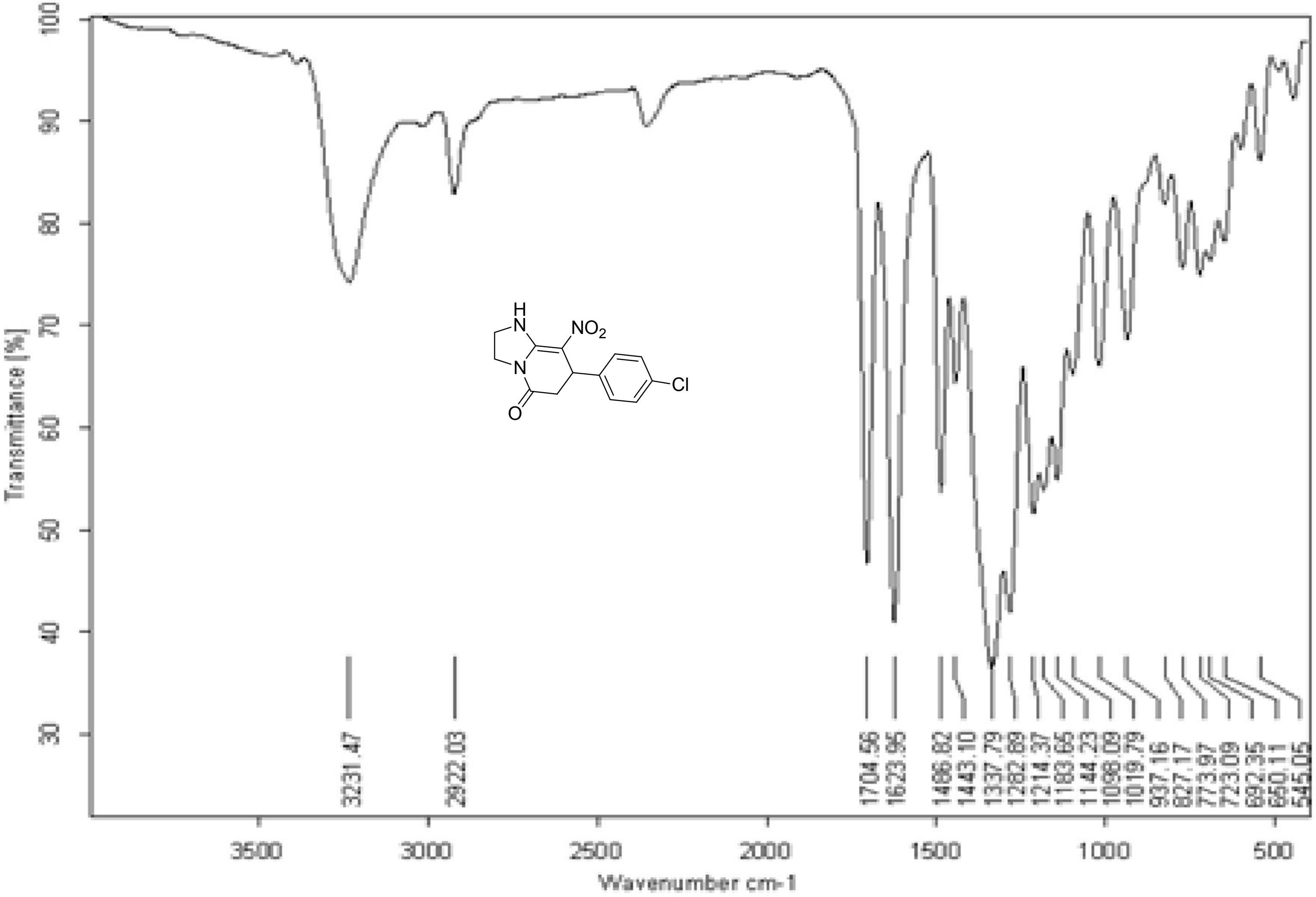
Transmittance [%]
Wavenumber cm-1
100
90
80
70
60
50
40
30
3500
3000
2500
2000
1500
1000
500
3231.47
2922.03
1704.56
1623.95
1486.82
1443.10
1337.79
1282.89
1214.37
1183.66
1144.23
1098.09
1019.79
937.16
827.17
773.97
723.09
692.35
650.11
545.05
H
N
NO2
N
O
Cl

IR of 5a

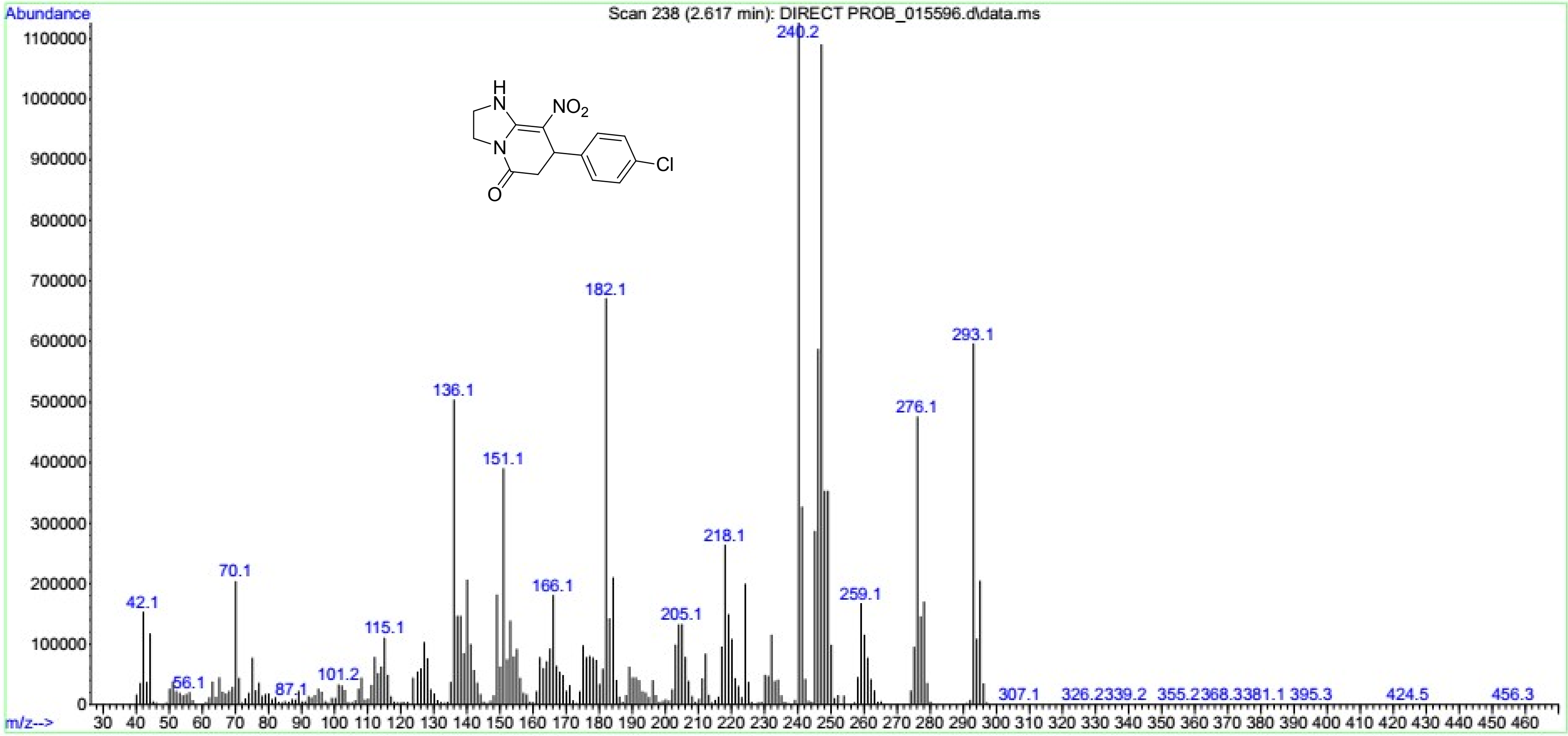
Abundance
Scan 238 (2.617 min): DIRECT PROB_015596.d\data.ms
240.2
293.1
276.1
182.1
136.1
151.1
218.1
259.1
166.1
205.1
115.1
101.2
87.1
70.1
56.1
42.1
307.1
326.2339.2
355.2368.3381.1
395.3
424.5
456.3
m/z-->

Mass of 5a

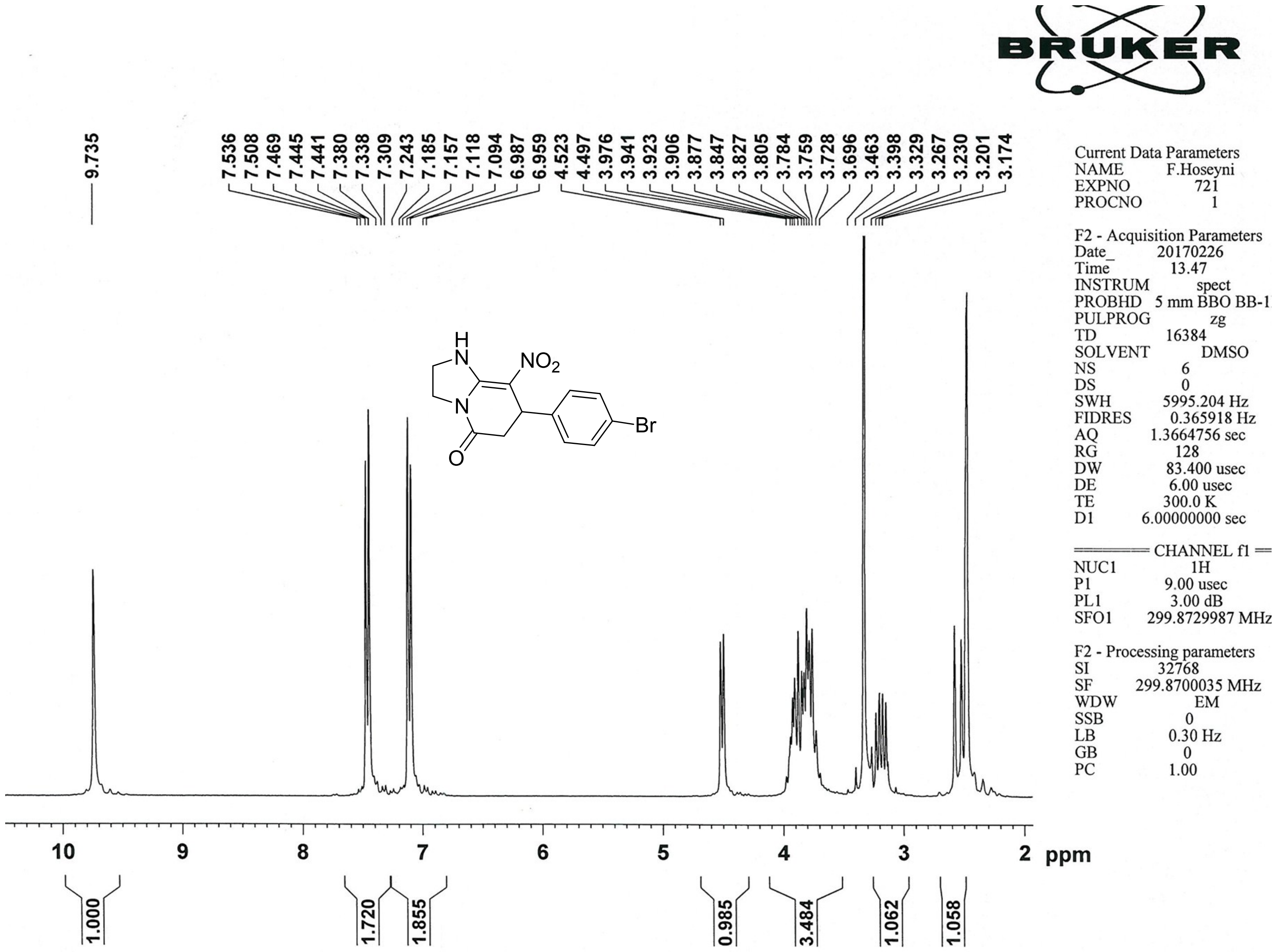
BRUKER
9.735
7.536
7.508
7.469
7.445
7.441
7.380
7.338
7.309
7.243
7.185
7.157
7.118
7.094
6.987
6.959
4.523
4.497
3.976
3.941
3.923
3.906
3.877
3.847
3.827
3.805
3.784
3.759
3.728
3.696
3.463
3.398
3.329
3.267
3.230
3.201
3.174
H
N
NO2
N
O
Br
Current Data Parameters
NAME F.Hoseyni
EXPNO 721
PROCNO 1
F2 - Acquisition Parameters
Date_ 20170226
Time 13.47
INSTRUM spect
PROBHD 5 mm BBO BB-1
PULPROG zg
TD 16384
SOLVENT DMSO
NS 6
DS 0
SWH 5995.204 Hz
FIDRES 0.365918 Hz
AQ 1.3664756 sec
RG 128
DW 83.400 usec
DE 6.00 usec
TE 300.0 K
D1 6.00000000 sec
CHANNEL f1
NUC1 1H
P1 9.00 usec
PL1 3.00 dB
SFO1 299.8729987 MHz
F2 - Processing parameters
SI 32768
SF 299.8700035 MHz
WDW EM
SSB 0
LB 0.30 Hz
GB 0
PC 1.00
10
9
8
7
6
5
4
3
2
ppm
1.000
1.720
1.855
0.985
3.484
1.062
1.058

^{1}H NMR of 5b

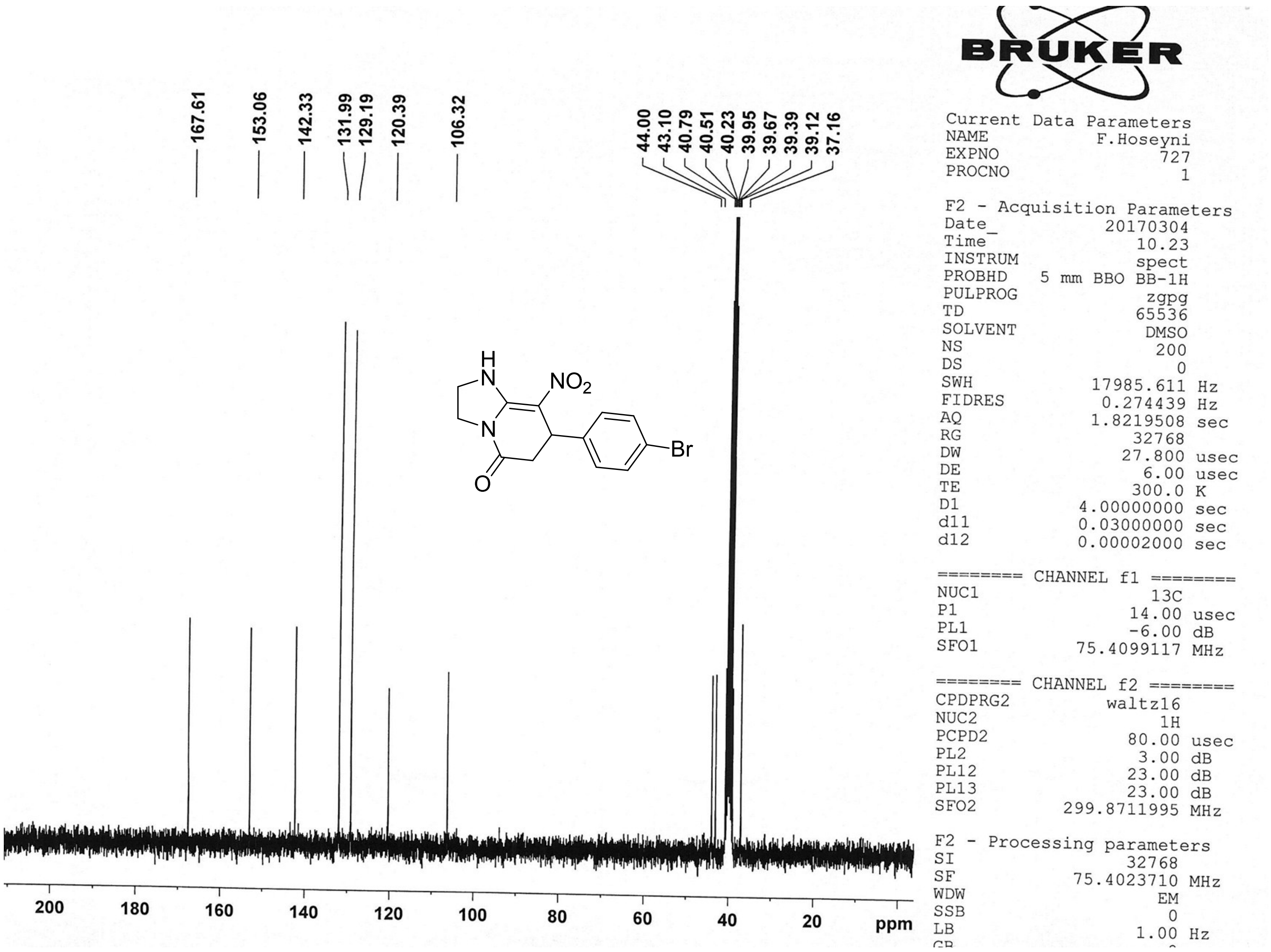
167.61
153.06
142.33
131.99
129.19
120.39
106.32
44.00
43.10
40.79
40.51
40.23
39.95
39.67
39.39
39.12
37.16
H
N
NO2
N
Br
O
200
180
160
140
120
100
80
60
40
20
ppm
BRUKER
Current Data Parameters
NAME F.Hoseyni
EXPNO 727
PROCNO 1
F2 - Acquisition Parameters
Date_ 20170304
Time 10.23
INSTRUM spect
PROBHD 5 mm BBO BB-1H
PULPROG zgpg
TD 65536
SOLVENT DMSO
NS 200
DS 0
SWH 17985.611 Hz
FIDRES 0.274439 Hz
AQ 1.8219508 sec
RG 32768
DW 27.800 usec
DE 6.00 usec
TE 300.0 K
D1 4.00000000 sec
d11 0.03000000 sec
d12 0.00002000 sec
======== CHANNEL f1 ========
NUC1 13C
P1 14.00 usec
PL1 -6.00 dB
SFO1 75.4099117 MHz
======== CHANNEL f2 ========
CPDPRG2 waltz16
NUC2 1H
PCPD2 80.00 usec
PL2 3.00 dB
PL12 23.00 dB
PL13 23.00 dB
SFO2 299.8711995 MHz
F2 - Processing parameters
SI 32768
SF 75.4023710 MHz
WDW EM
SSB 0
LB 1.00 Hz

^{13}C NMR of 5b

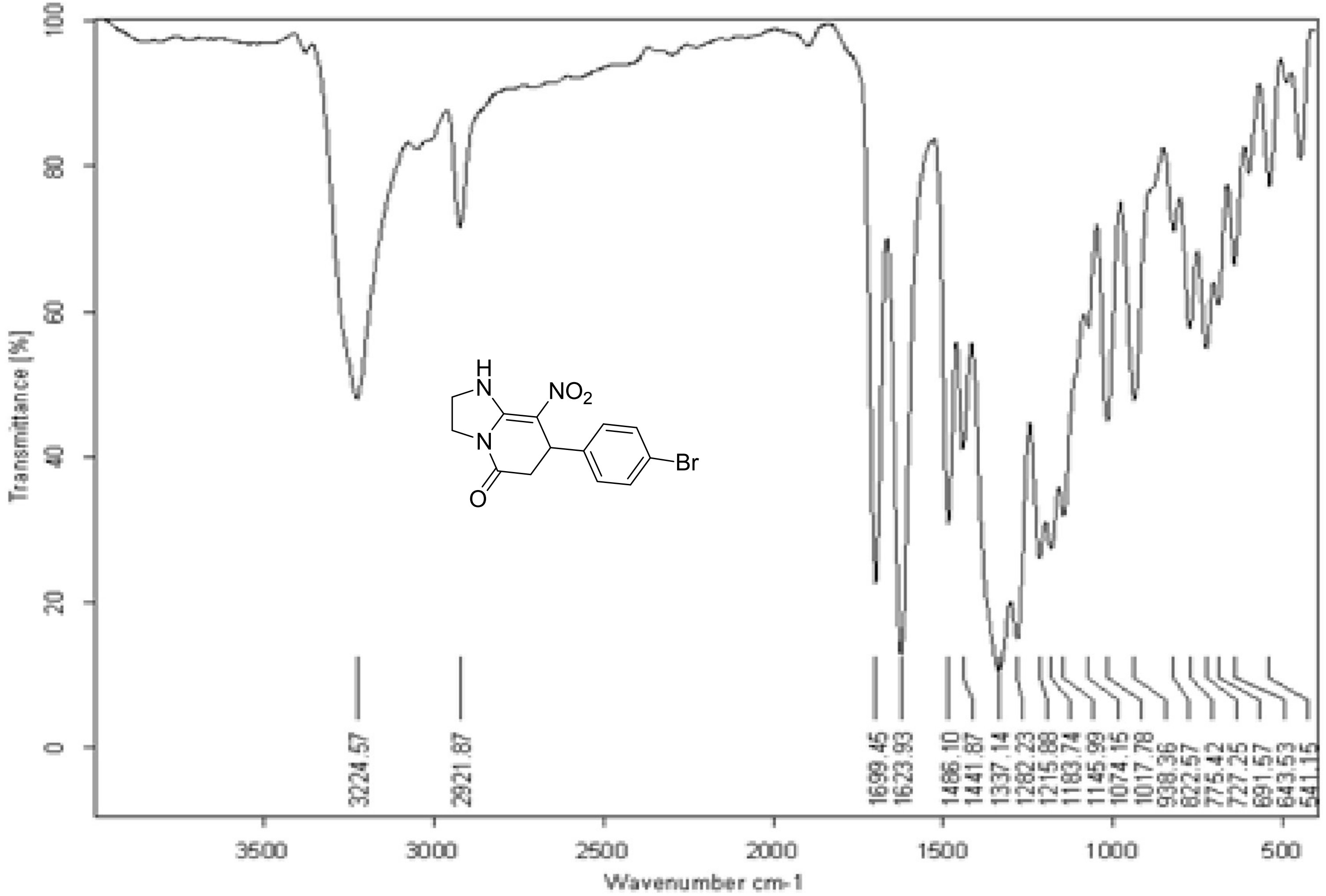

Transmittance [%]
Wavenumber cm-1
NO2
Br
3224.57
2921.87
1699.45
1623.93
1486.10
1441.87
1337.14
1282.23
1215.88
1183.74
1145.99
1074.15
1017.78
938.36
822.57
775.42
727.25
691.57
643.53
541.15

IR of 5b

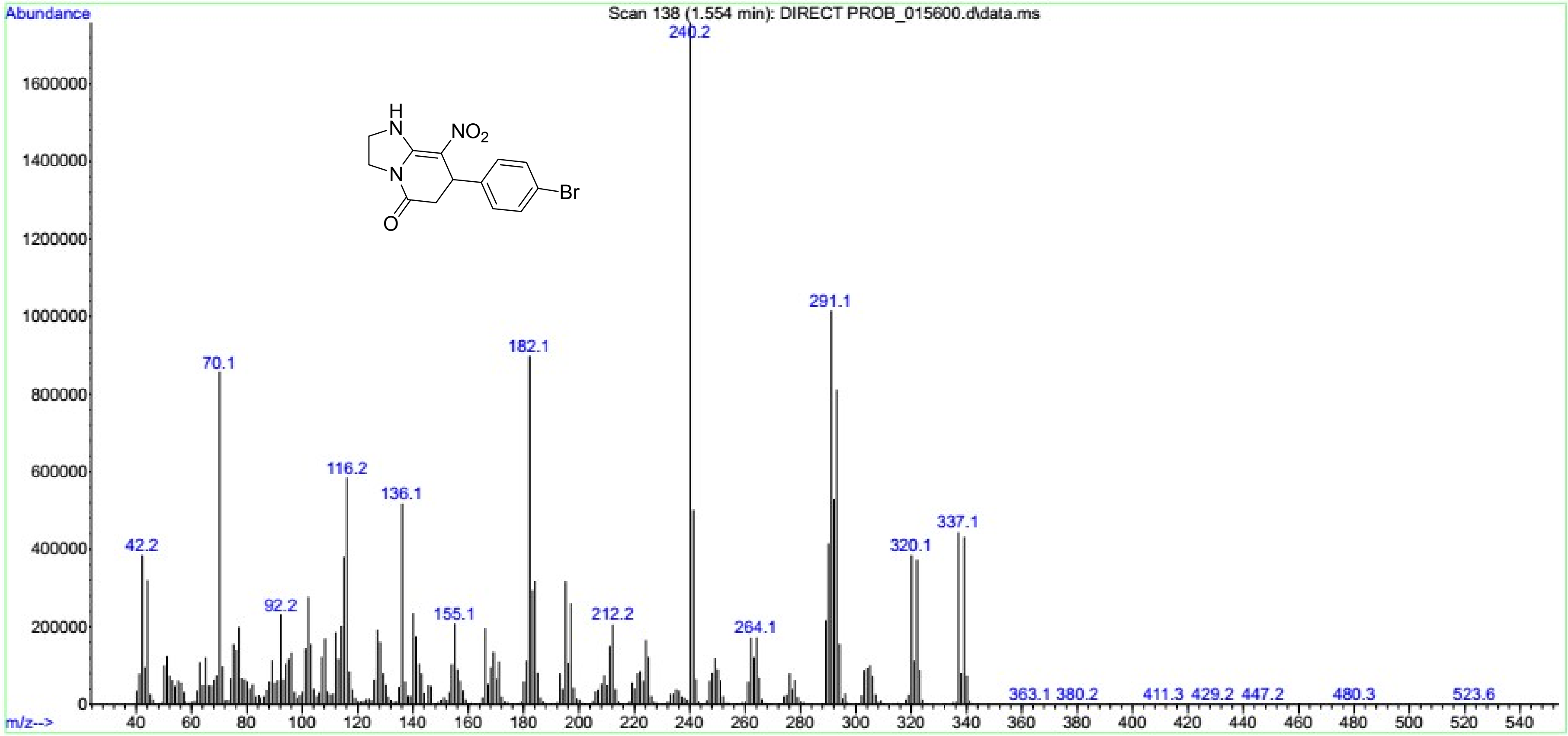
Abundance
Scan 138 (1.554 min): DIRECT PROB_015600.d\data.ms
240.2
291.1
182.1
70.1
116.2
136.1
42.2
337.1
320.1
92.2
155.1
212.2
264.1
363.1 380.2 411.3 429.2 447.2 480.3 523.6
m/z-->
NO2
Br

Mass of 5b

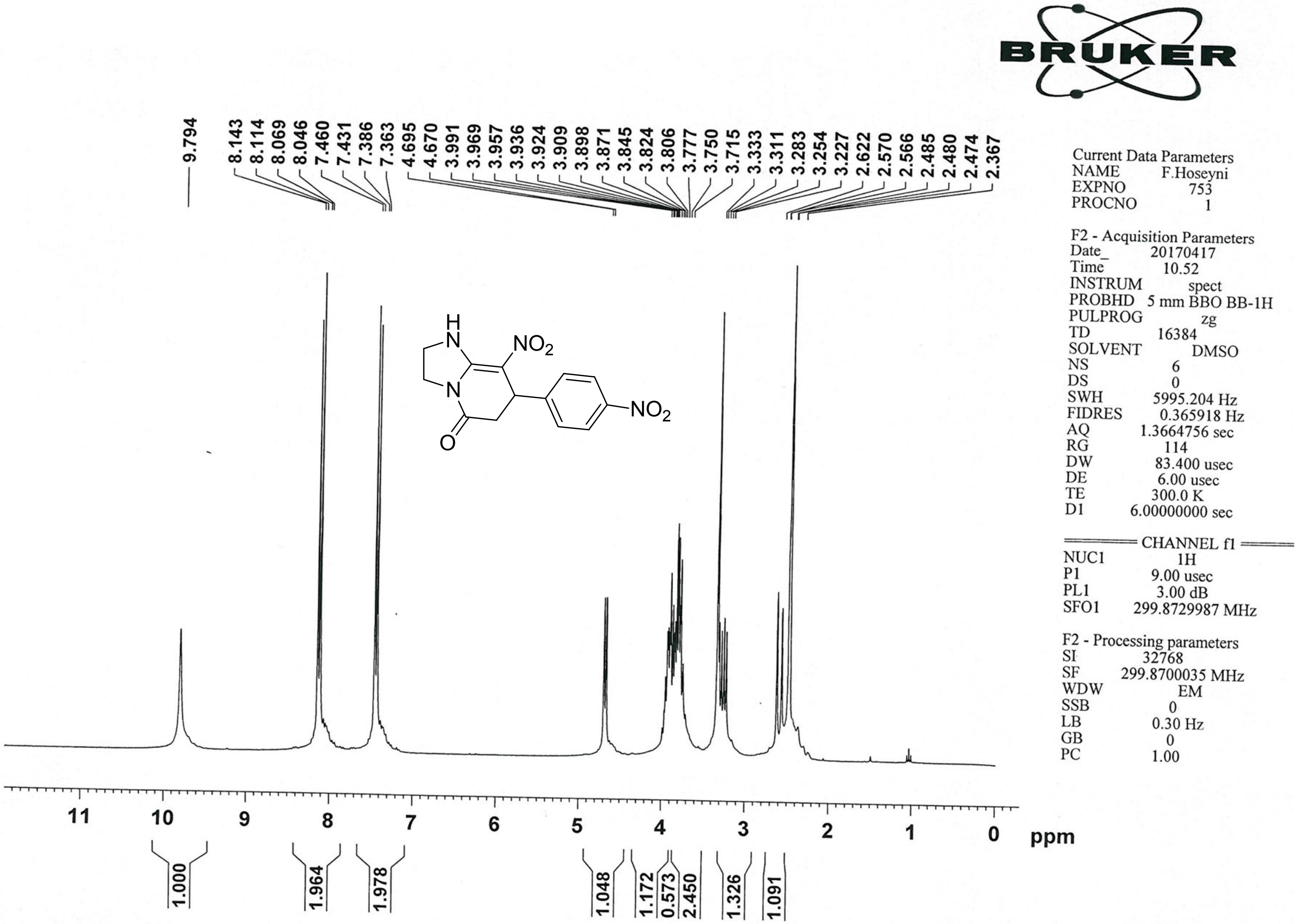
9.794
8.143
8.114
8.069
8.046
7.460
7.431
7.386
7.363
4.695
4.670
3.991
3.969
3.957
3.936
3.924
3.909
3.898
3.871
3.845
3.824
3.806
3.777
3.750
3.715
3.333
3.311
3.283
3.254
3.227
2.622
2.570
2.566
2.485
2.480
2.474
2.367
H
N
NO2
N
NO2
O
Current Data Parameters
NAME F.Hoseyni
EXPNO 753
PROCNO 1
F2 - Acquisition Parameters
Date_ 20170417
Time 10.52
INSTRUM spect
PROBHD 5 mm BBO BB-1H
PULPROG zg
TD 16384
SOLVENT DMSO
NS 6
DS 0
SWH 5995.204 Hz
FIDRES 0.365918 Hz
AQ 1.3664756 sec
RG 114
DW 83.400 usec
DE 6.00 usec
TE 300.0 K
D1 6.00000000 sec
CHANNEL f1
NUC1 1H
P1 9.00 usec
PL1 3.00 dB
SFO1 299.8729987 MHz
F2 - Processing parameters
SI 32768
SF 299.8700035 MHz
WDW EM
SSB 0
LB 0.30 Hz
GB 0
PC 1.00
11 10 9 8 7 6 5 4 3 2 1 0 ppm
1.000
1.964
1.978
1.048
1.172
0.573
2.450
1.326
1.091

^{1}H NMR of 5c

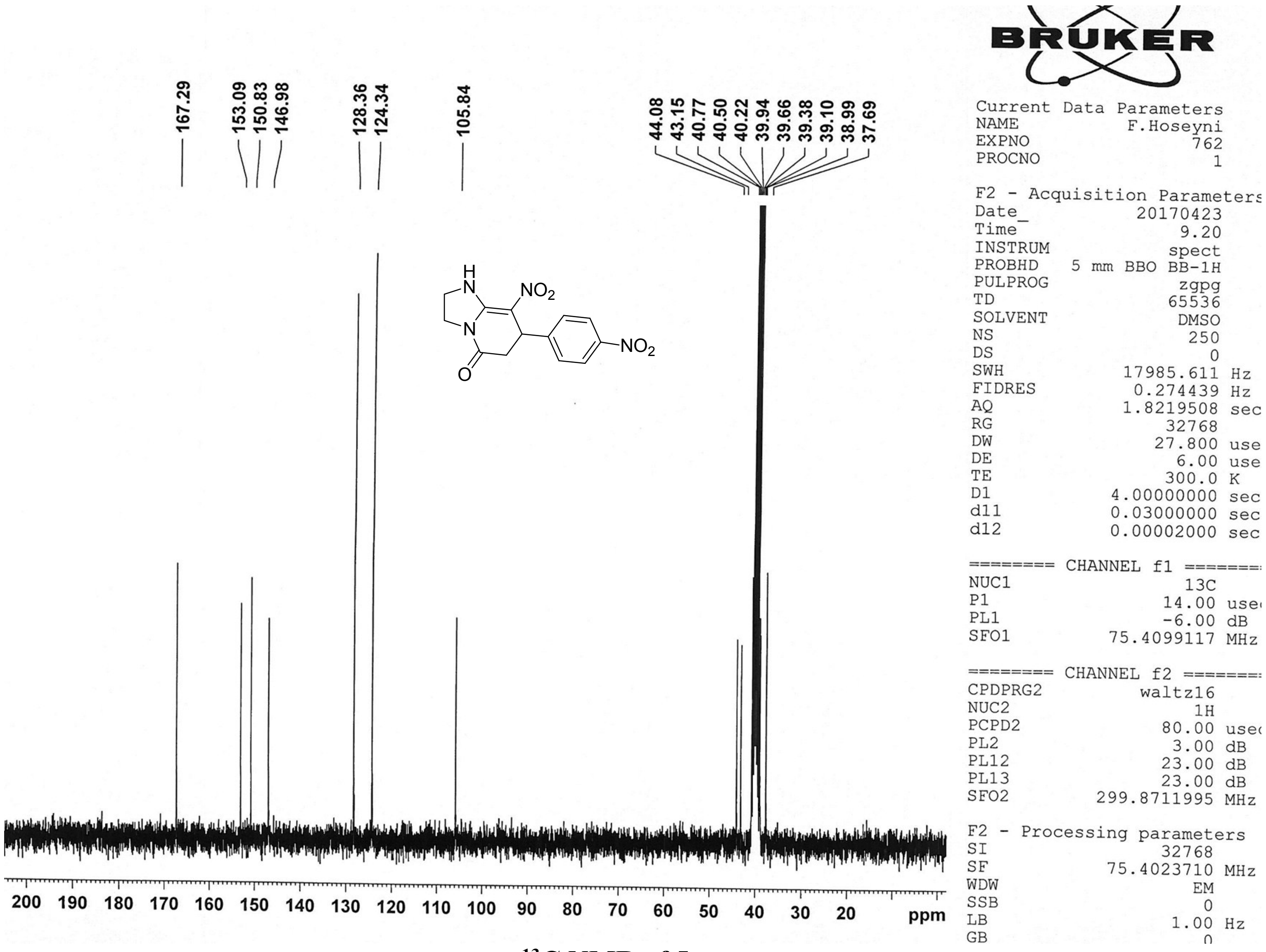
167.29
153.09
150.83
146.98
128.36
124.34
105.84
44.08
43.15
40.77
40.50
40.22
39.94
39.66
39.38
39.10
38.99
37.69
H
N
NO2
N
NO2
O
BRUKER
Current Data Parameters
NAME F.Hoseyni
EXPNO 762
PROCNO 1
F2 - Acquisition Parameters
Date_ 20170423
Time 9.20
INSTRUM spect
PROBHD 5 mm BBO BB-1H
PULPROG zgpg
TD 65536
SOLVENT DMSO
NS 250
DS 0
SWH 17985.611 Hz
FIDRES 0.274439 Hz
AQ 1.8219508 sec
RG 32768
DW 27.800 use
DE 6.00 use
TE 300.0 K
D1 4.00000000 sec
d11 0.03000000 sec
d12 0.00002000 sec
======== CHANNEL f1 ========
NUC1 13C
P1 14.00 use
PL1 -6.00 dB
SFO1 75.4099117 MHz
======== CHANNEL f2 ========
CPDPRG2 waltz16
NUC2 1H
PCPD2 80.00 use
PL2 3.00 dB
PL12 23.00 dB
PL13 23.00 dB
SFO2 299.8711995 MHz
F2 - Processing parameters
SI 32768
SF 75.4023710 MHz
WDW EM
SSB 0
LB 1.00 Hz
GB 0
200 190 180 170 160 150 140 130 120 110 100 90 80 70 60 50 40 30 20 ppm

^{13}C NMR of 5c

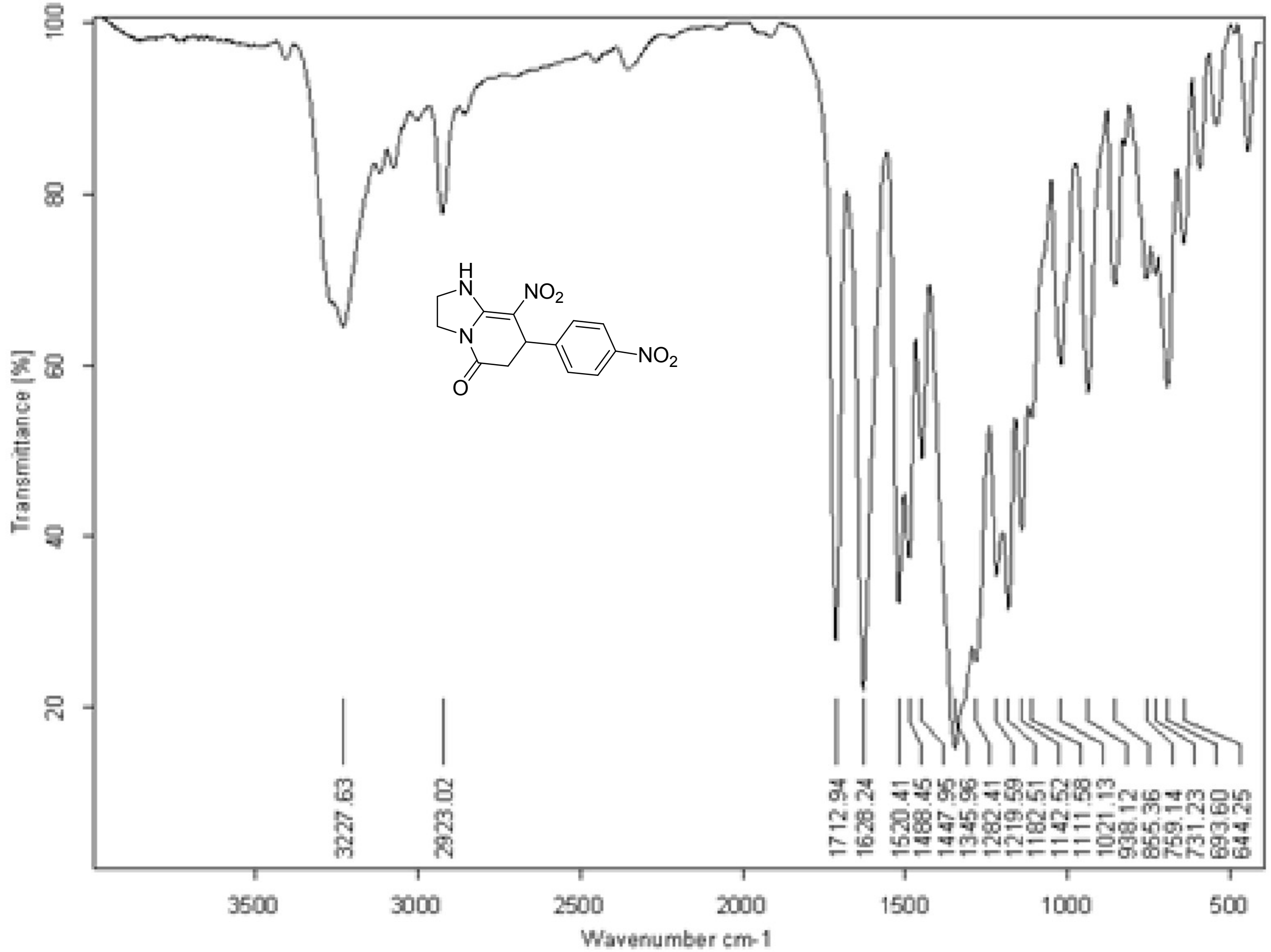
Transmittance [%]
Wavenumber cm-1
3227.63
2923.02
1712.94
1628.24
1520.41
1488.45
1447.95
1345.96
1282.41
1219.59
1182.51
1142.52
1111.58
1021.13
938.12
865.36
759.14
731.23
693.60
644.25
NO2
NO2
H
N
N
O

IR of 5c

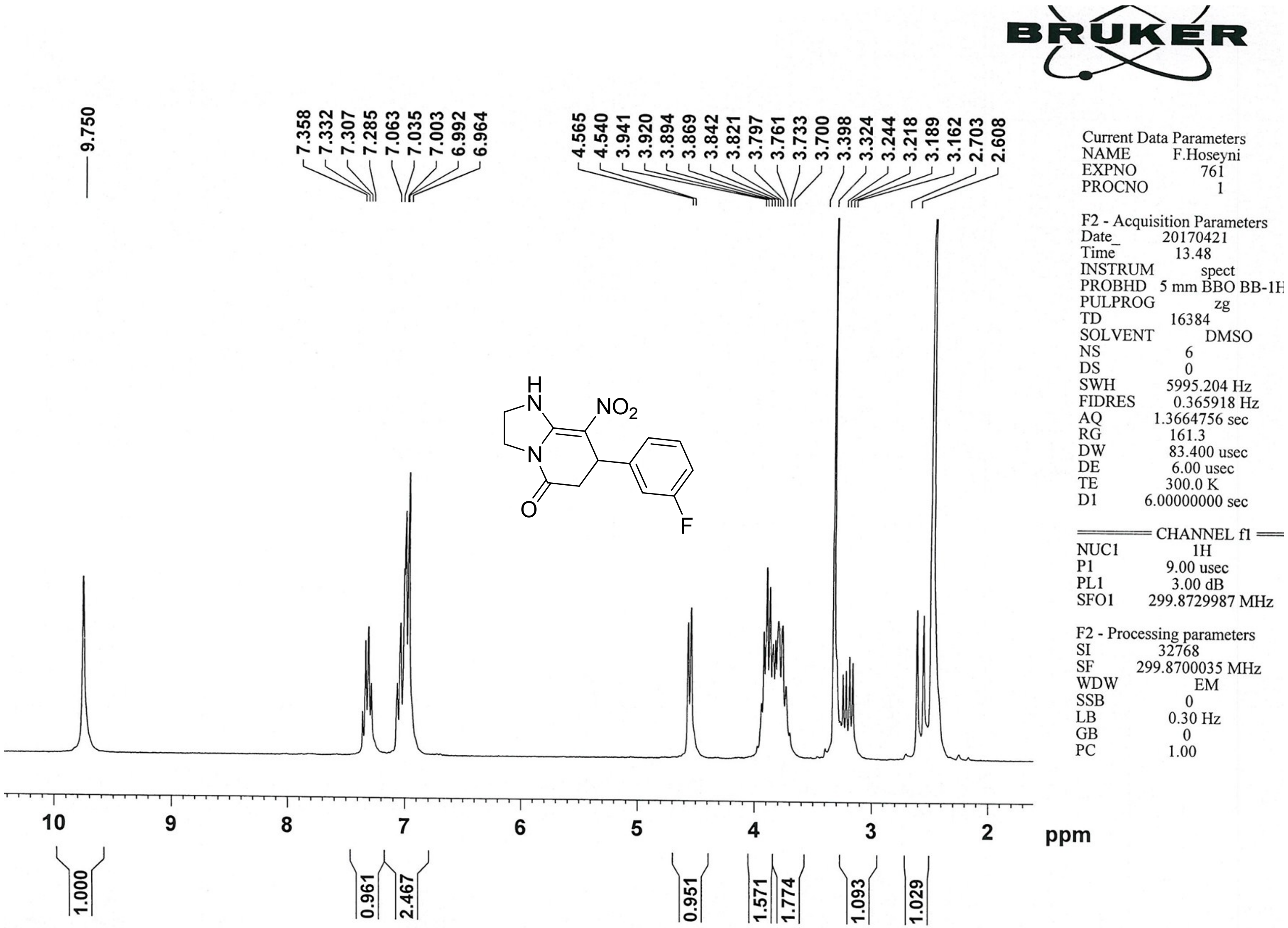
BRUKER
9.750
7.358
7.332
7.307
7.285
7.063
7.035
7.003
6.992
6.964
4.565
4.540
3.941
3.920
3.894
3.869
3.842
3.821
3.797
3.761
3.733
3.700
3.398
3.324
3.244
3.218
3.189
3.162
2.703
2.608
H
N
NO2
N
O
F
10 9 8 7 6 5 4 3 2 ppm
1.000
0.961
2.467
0.951
1.571
1.774
1.093
1.029
Current Data Parameters
NAME F.Hoseyni
EXPNO 761
PROCNO 1
F2 - Acquisition Parameters
Date_ 20170421
Time 13.48
INSTRUM spect
PROBHD 5 mm BBO BB-1H
PULPROG zg
TD 16384
SOLVENT DMSO
NS 6
DS 0
SWH 5995.204 Hz
FIDRES 0.365918 Hz
AQ 1.3664756 sec
RG 161.3
DW 83.400 usec
DE 6.00 usec
TE 300.0 K
D1 6.00000000 sec
CHANNEL f1
NUC1 1H
P1 9.00 usec
PL1 3.00 dB
SFO1 299.8729987 MHz
F2 - Processing parameters
SI 32768
SF 299.8700035 MHz
WDW EM
SSB 0
LB 0.30 Hz
GB 0
PC 1.00

^{1}H NMR of 5d

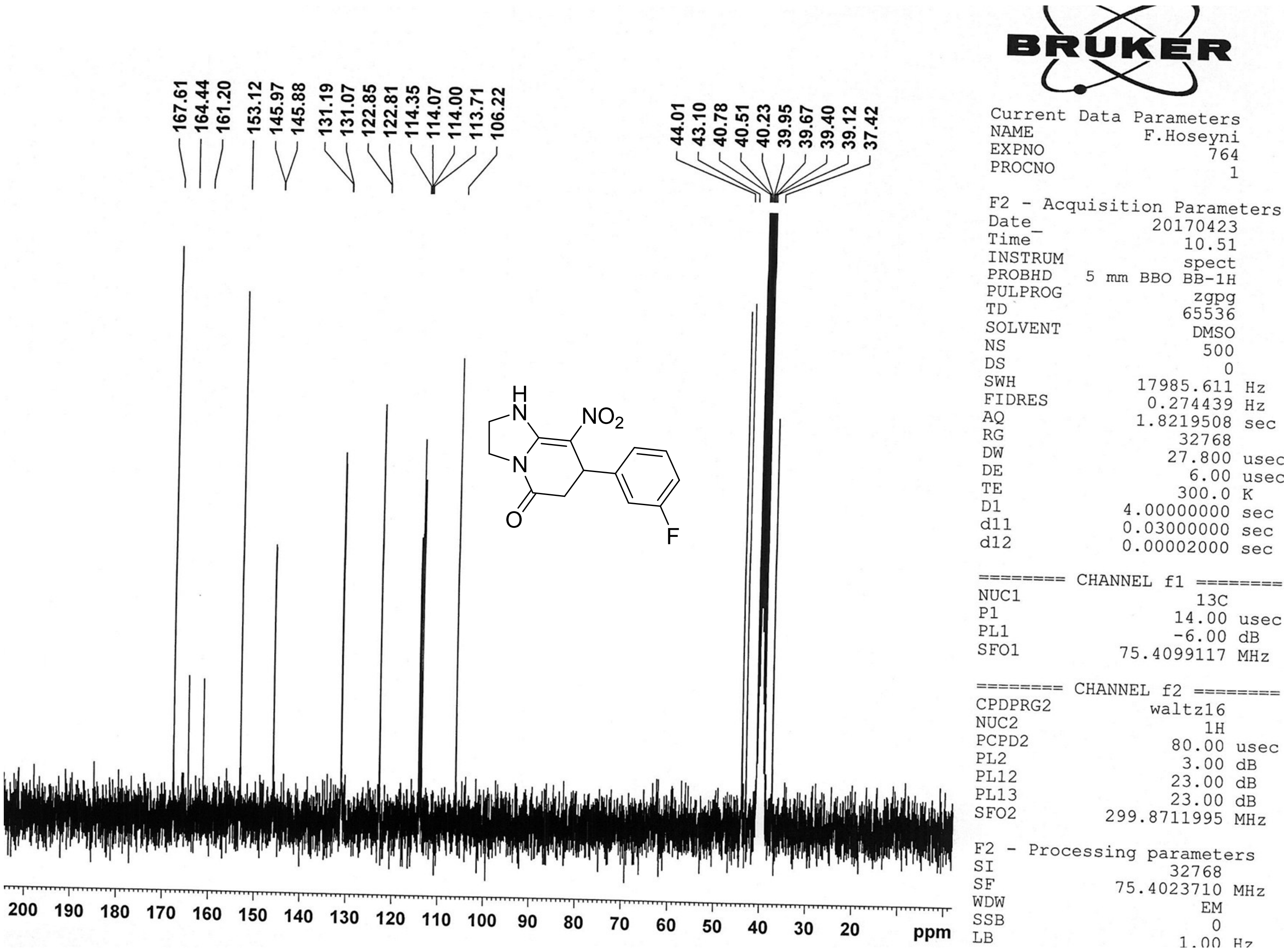
167.61
164.44
161.20
153.12
145.97
145.88
131.19
131.07
122.85
122.81
114.35
114.07
114.00
113.71
106.22
44.01
43.10
40.78
40.51
40.23
39.95
39.67
39.40
39.12
37.42
H
N
NO2
N
O
F
200 190 180 170 160 150 140 130 120 110 100 90 80 70 60 50 40 30 20 ppm
BRUKER
Current Data Parameters
NAME F.Hoseyni
EXPNO 764
PROCNO 1
F2 - Acquisition Parameters
Date_ 20170423
Time 10.51
INSTRUM spect
PROBHD 5 mm BBO BB-1H
PULPROG zgpg
TD 65536
SOLVENT DMSO
NS 500
DS 0
SWH 17985.611 Hz
FIDRES 0.274439 Hz
AQ 1.8219508 sec
RG 32768
DW 27.800 usec
DE 6.00 usec
TE 300.0 K
D1 4.00000000 sec
d11 0.03000000 sec
d12 0.00002000 sec
======== CHANNEL f1 ========
NUC1 13C
P1 14.00 usec
PL1 -6.00 dB
SFO1 75.4099117 MHz
======== CHANNEL f2 ========
CPDPRG2 waltz16
NUC2 1H
PCPD2 80.00 usec
PL2 3.00 dB
PL12 23.00 dB
PL13 23.00 dB
SFO2 299.8711995 MHz
F2 - Processing parameters
SI 32768
SF 75.4023710 MHz
WDW EM
SSB 0
LB 1.00 Hz

^{13}C NMR of 5d

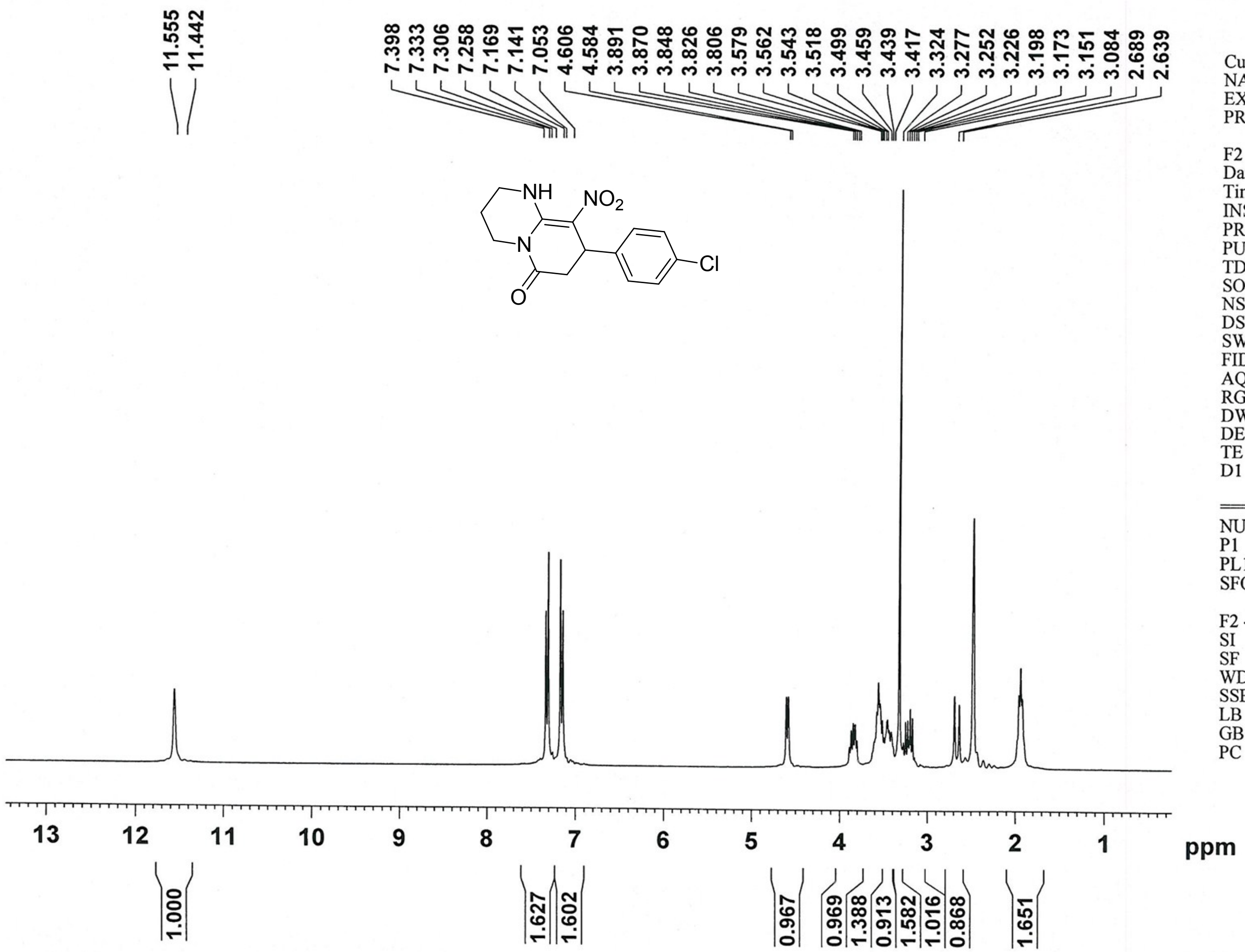

Current Data Parameters
NAME F.Hoseyni
EXPNO 731
PROCNO 1

F2 - Acquisition Parameters
Date_ 20170305
Time 15.53
INSTRUM spect
PROBHD 5 mm BBO BB-1H
PULPROG zg
TD 16384
SOLVENT DMSO
NS 6
DS 0
SWH 5995.204 Hz
FIDRES 0.365918 Hz
AQ 1.3664756 sec
RG 181
DW 83.400 usec
DE 6.00 usec
TE 300.0 K
D1 6.00000000 sec

======== CHANNEL f1 ========
NUC1 1H
P1 9.00 usec
PL1 3.00 dB
SFO1 299.8729987 MHz

F2 - Processing parameters
SI 32768
SF 299.8700035 MHz
WDW EM
SSB 0
LB 0.30 Hz
GB 0
PC 1.00

^{1}H NMR of 5e

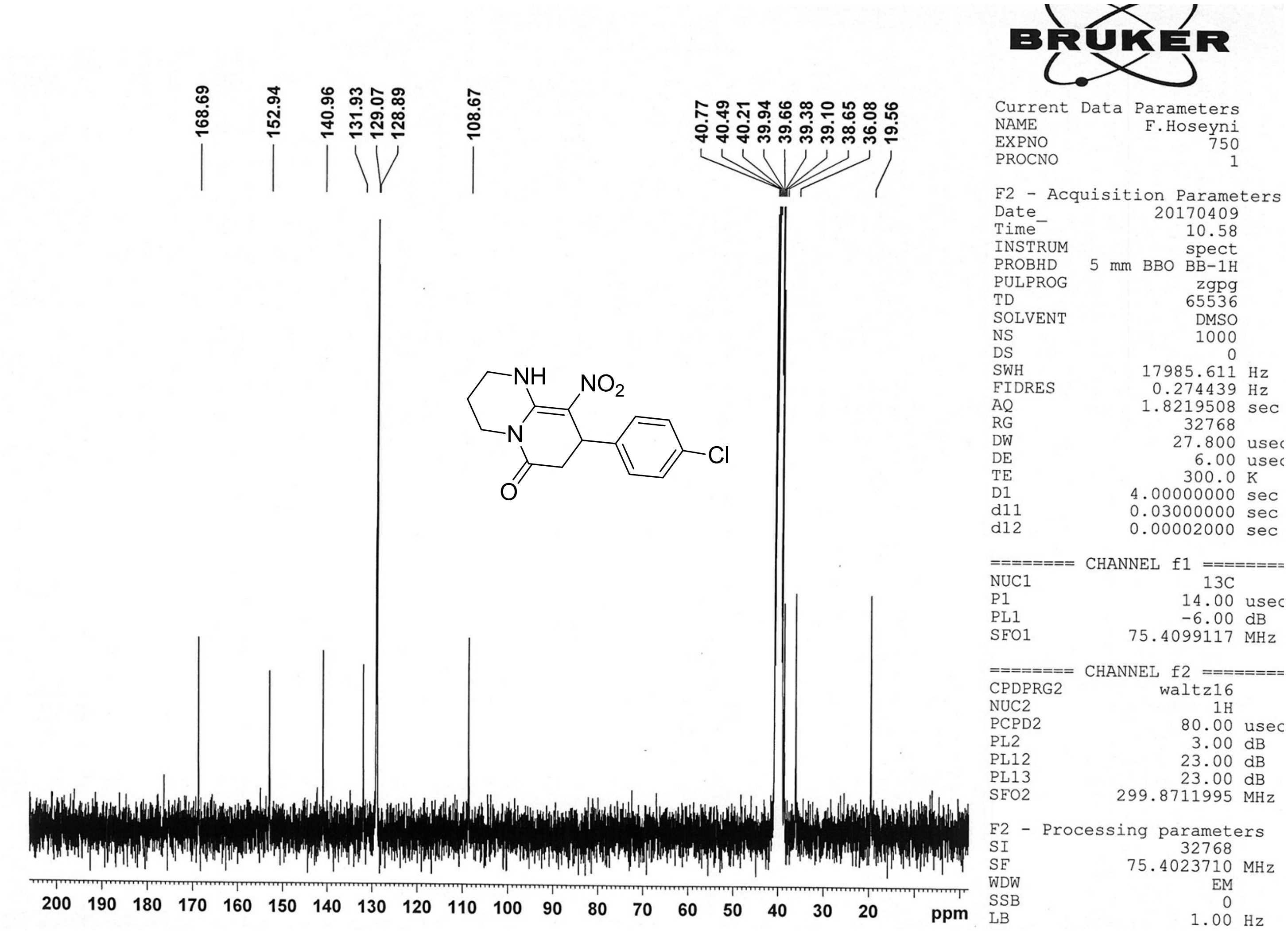
168.69
152.94
140.96
131.93
129.07
128.89
108.67
40.77
40.49
40.21
39.94
39.66
39.38
39.10
38.65
36.08
19.56
NH
NO2
N
Cl
O
200 190 180 170 160 150 140 130 120 110 100 90 80 70 60 50 40 30 20 ppm
BRUKER
Current Data Parameters
NAME F.Hoseyni
EXPNO 750
PROCNO 1
F2 - Acquisition Parameters
Date_ 20170409
Time 10.58
INSTRUM spect
PROBHD 5 mm BBO BB-1H
PULPROG zgpg
TD 65536
SOLVENT DMSO
NS 1000
DS 0
SWH 17985.611 Hz
FIDRES 0.274439 Hz
AQ 1.8219508 sec
RG 32768
DW 27.800 usec
DE 6.00 usec
TE 300.0 K
D1 4.00000000 sec
d11 0.03000000 sec
d12 0.00002000 sec
======== CHANNEL f1 ========
NUC1 13C
P1 14.00 usec
PL1 -6.00 dB
SFO1 75.4099117 MHz
======== CHANNEL f2 ========
CPDPRG2 waltz16
NUC2 1H
PCPD2 80.00 usec
PL2 3.00 dB
PL12 23.00 dB
PL13 23.00 dB
SFO2 299.8711995 MHz
F2 - Processing parameters
SI 32768
SF 75.4023710 MHz
WDW EM
SSB 0
LB 1.00 Hz

^{13}C NMR of 5e

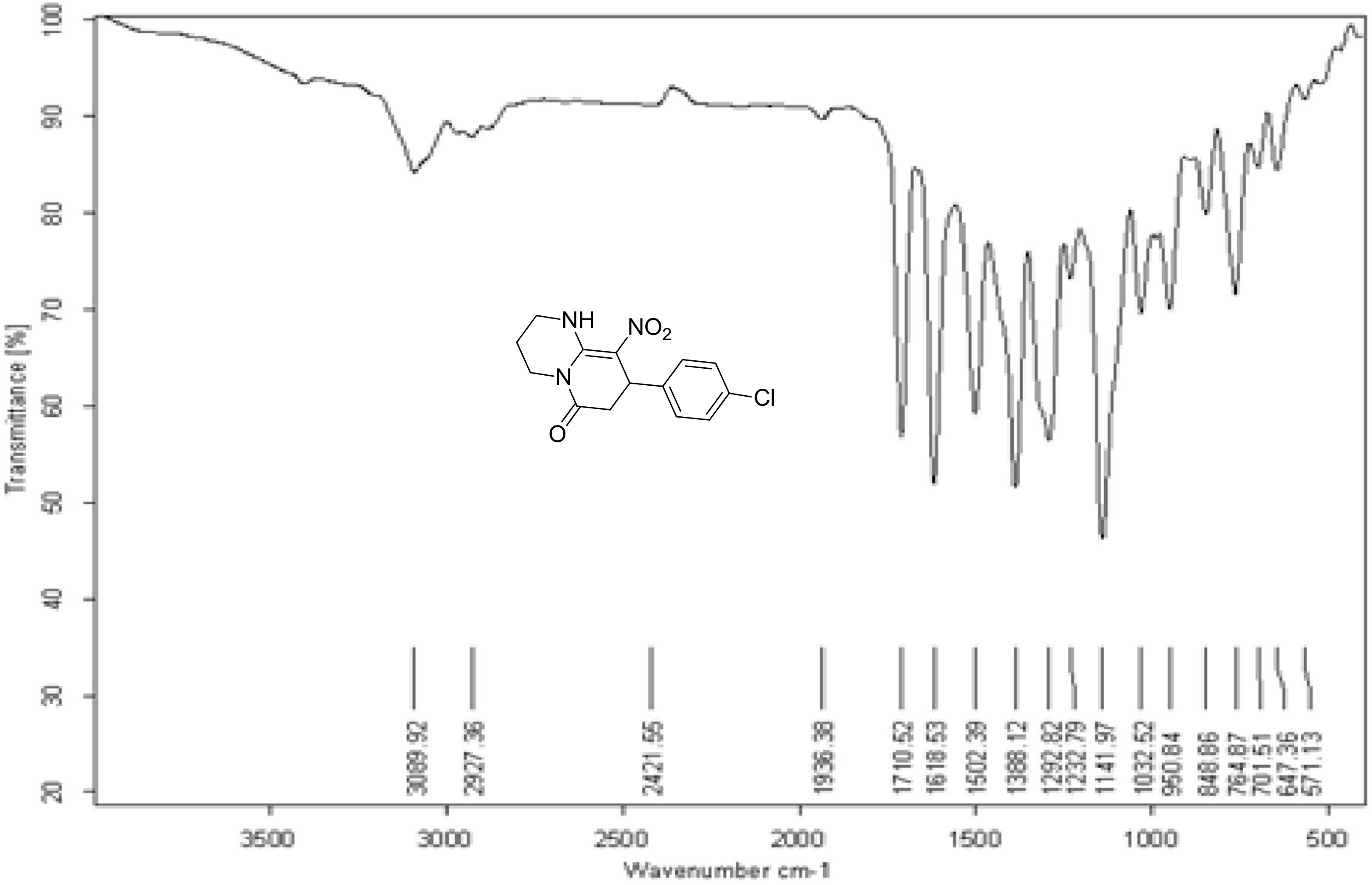
Transmittance [%]
100
90
80
70
60
50
40
30
20
NH
NO2
N
O
Cl
3089.92
2927.36
2421.65
1936.38
1710.62
1618.53
1502.39
1388.12
1292.82
1232.79
1141.97
1032.52
950.84
848.86
764.87
701.51
647.36
571.13
3500
3000
2500
2000
1500
1000
500
Wavenumber cm-1

IR of 5e

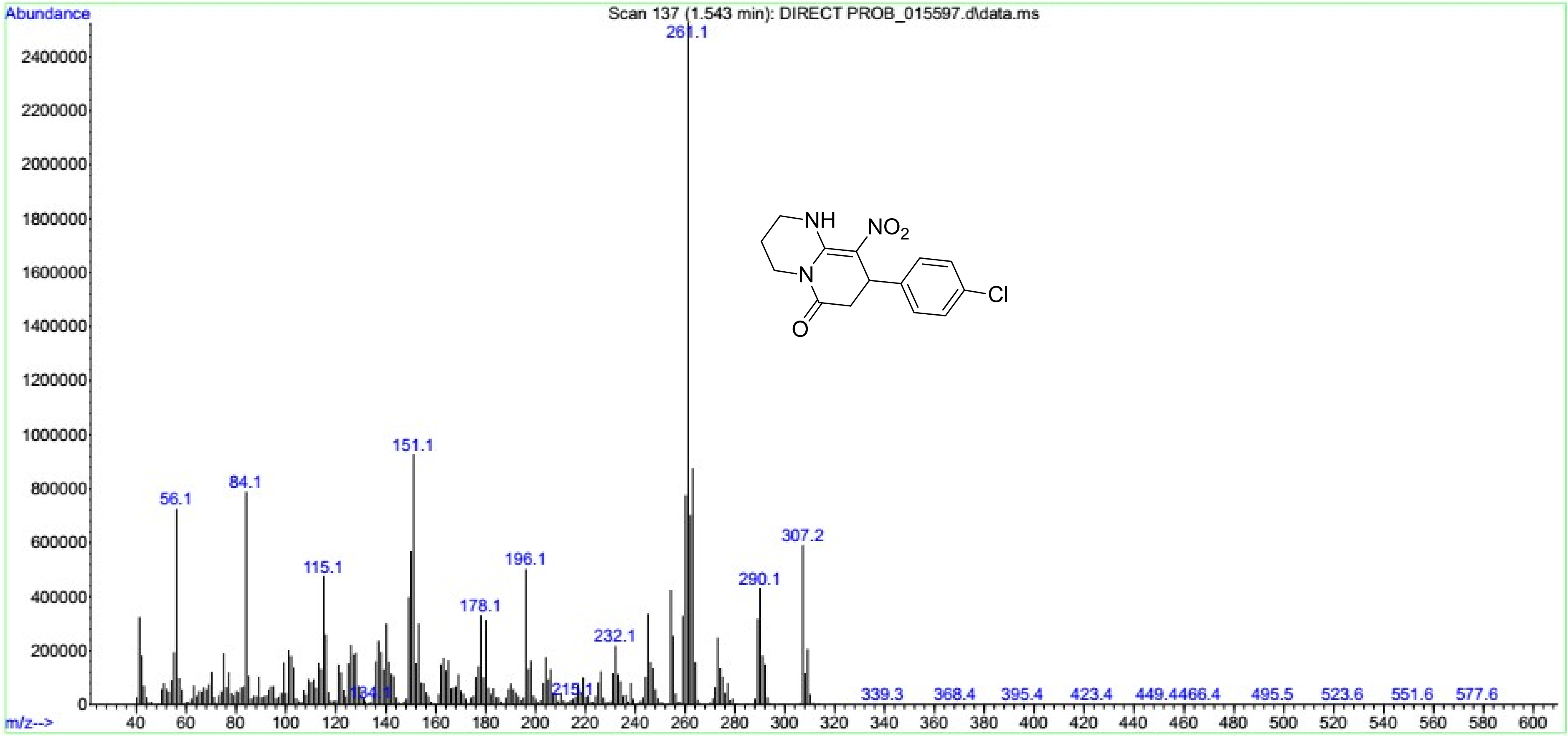
Abundance
Scan 137 (1.543 min): DIRECT PROB_015597.d\data.ms
261.1
151.1
84.1
56.1
307.2
196.1
115.1
290.1
178.1
232.1
339.3
368.4
395.4
423.4
449.4466.4
495.5
523.6
551.6
577.6
m/z-->
NH
NO_2
N
Cl
O

Mass of 5e

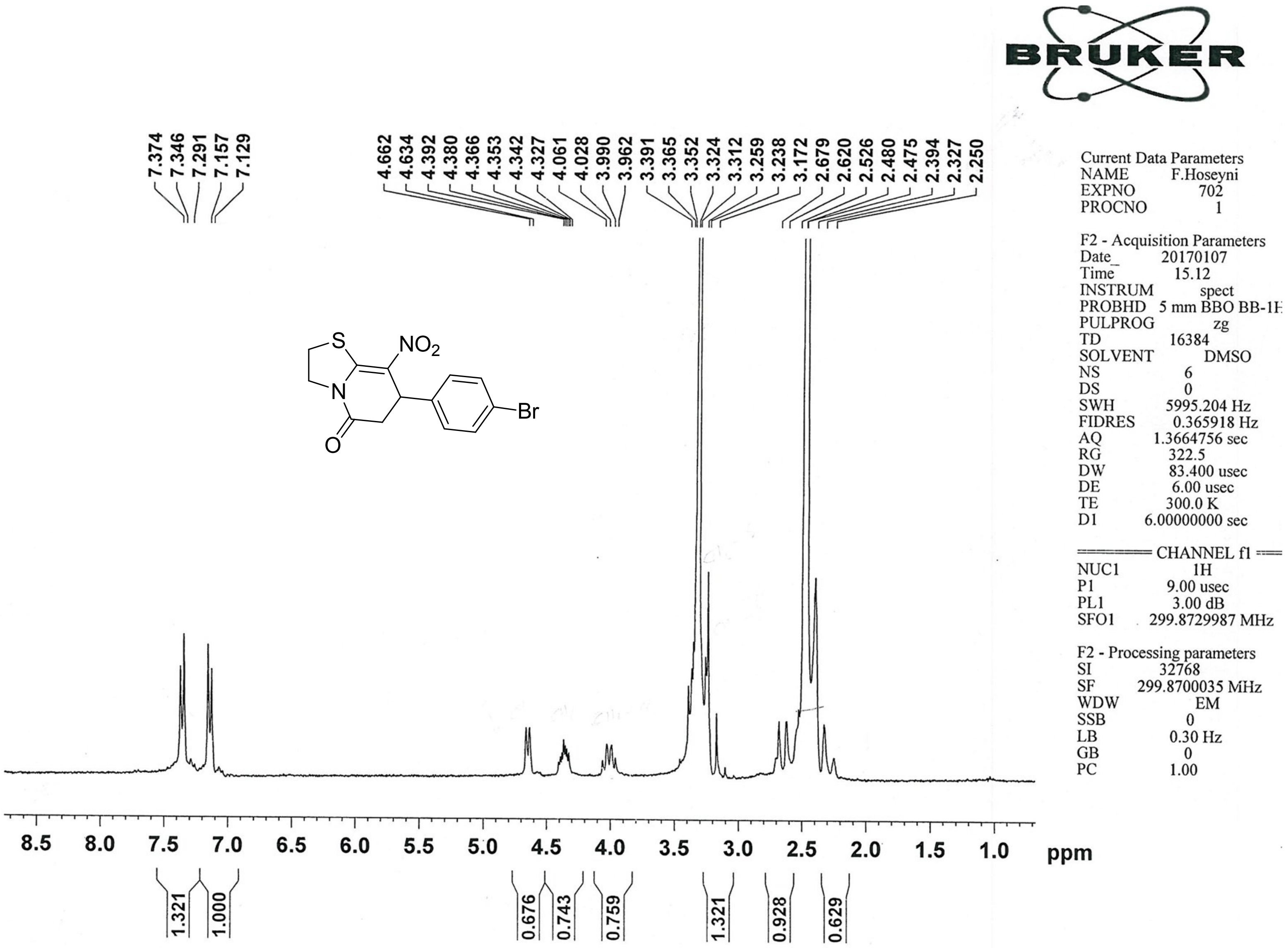
BRUKER
7.374
7.346
7.291
7.157
7.129
4.662
4.634
4.392
4.380
4.366
4.353
4.342
4.327
4.061
4.028
3.990
3.962
3.391
3.365
3.352
3.324
3.312
3.259
3.238
3.172
2.679
2.620
2.526
2.480
2.475
2.394
2.327
2.250
S
NO2
N
Br
O
8.5 8.0 7.5 7.0 6.5 6.0 5.5 5.0 4.5 4.0 3.5 3.0 2.5 2.0 1.5 1.0 ppm
1.321
1.000
0.676
0.743
0.759
1.321
0.928
0.629
Current Data Parameters
NAME F.Hoseyni
EXPNO 702
PROCNO 1
F2 - Acquisition Parameters
Date_ 20170107
Time 15.12
INSTRUM spect
PROBHD 5 mm BBO BB-1H
PULPROG zg
TD 16384
SOLVENT DMSO
NS 6
DS 0
SWH 5995.204 Hz
FIDRES 0.365918 Hz
AQ 1.3664756 sec
RG 322.5
DW 83.400 usec
DE 6.00 usec
TE 300.0 K
D1 6.00000000 sec
CHANNEL f1
NUC1 1H
P1 9.00 usec
PL1 3.00 dB
SFO1 299.8729987 MHz
F2 - Processing parameters
SI 32768
SF 299.8700035 MHz
WDW EM
SSB 0
LB 0.30 Hz
GB 0
PC 1.00

^{1}H NMR of 5f

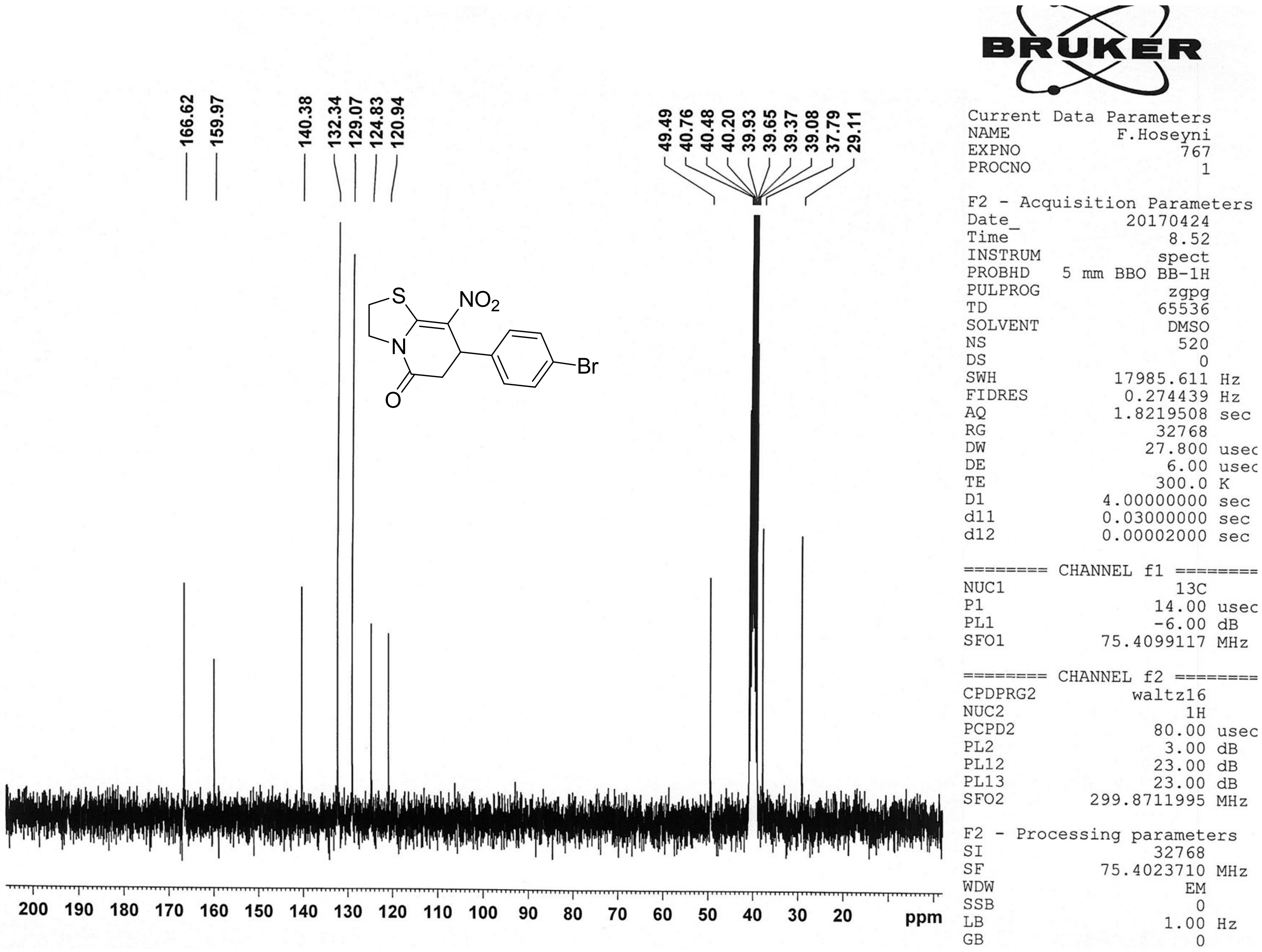

166.62
159.97
140.38
132.34
129.07
124.83
120.94
49.49
40.76
40.48
40.20
39.93
39.65
39.37
39.08
37.79
29.11
S
NO2
N
Br
O
200 190 180 170 160 150 140 130 120 110 100 90 80 70 60 50 40 30 20 ppm
BRUKER
Current Data Parameters
NAME F.Hoseyni
EXPNO 767
PROCNO 1
F2 - Acquisition Parameters
Date_ 20170424
Time 8.52
INSTRUM spect
PROBHD 5 mm BBO BB-1H
PULPROG zgpg
TD 65536
SOLVENT DMSO
NS 520
DS 0
SWH 17985.611 Hz
FIDRES 0.274439 Hz
AQ 1.8219508 sec
RG 32768
DW 27.800 usec
DE 6.00 usec
TE 300.0 K
D1 4.00000000 sec
d11 0.03000000 sec
d12 0.00002000 sec
======== CHANNEL f1 ========
NUC1 13C
P1 14.00 usec
PL1 -6.00 dB
SFO1 75.4099117 MHz
======== CHANNEL f2 ========
CPDPRG2 waltz16
NUC2 1H
PCPD2 80.00 usec
PL2 3.00 dB
PL12 23.00 dB
PL13 23.00 dB
SFO2 299.8711995 MHz
F2 - Processing parameters
SI 32768
SF 75.4023710 MHz
WDW EM
SSB 0
LB 1.00 Hz
GB 0

^{13}C NMR of 5f

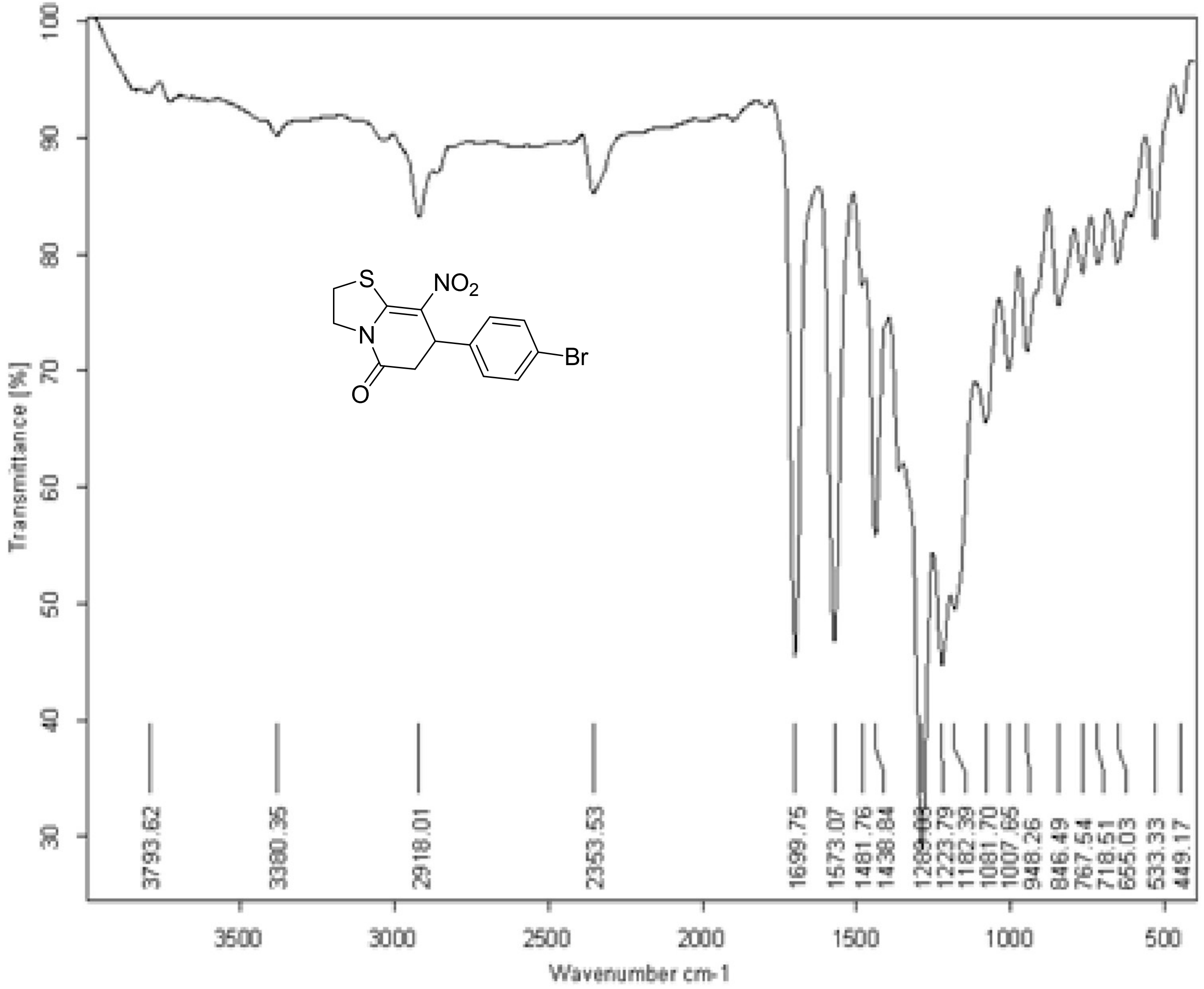

Transmittance [%]
Wavenumber cm-1
NO2
S
N
O
Br
3793.62
3380.35
2918.01
2353.53
1699.75
1573.07
1481.76
1438.84
1223.79
1182.39
1081.70
1007.66
948.26
846.49
767.54
718.51
655.03
533.33
449.17

IR of 5f

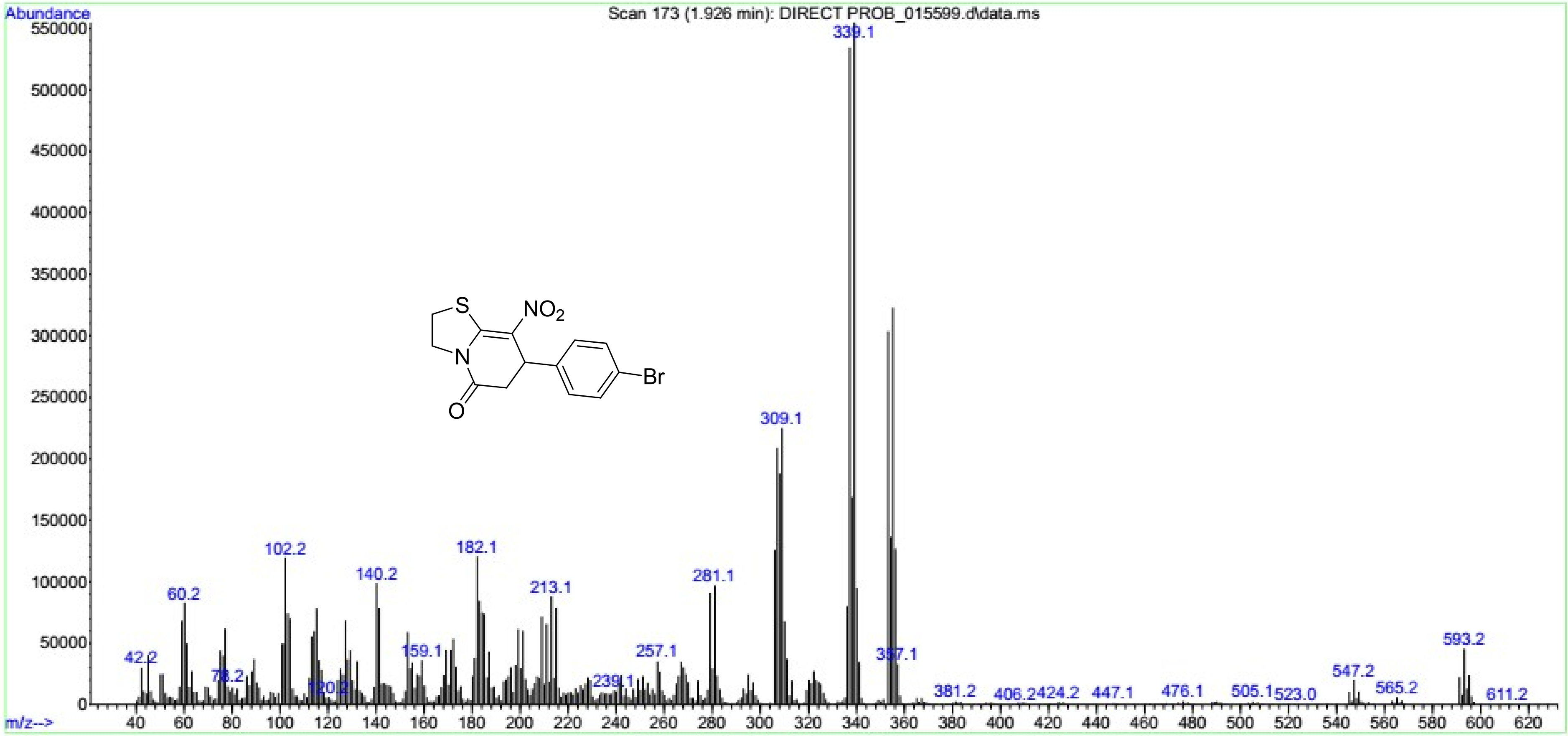
Abundance
Scan 173 (1.926 min): DIRECT PROB_015599.d\data.ms
m/z-->
NO2
S
N
O
Br
339.1
309.1
102.2
182.1
140.2
213.1
281.1
60.2
42.2
159.1
257.1
239.1
381.2
406.2424.2
447.1
476.1
505.1
523.0
547.2
565.2
593.2
611.2

Mass of 5f

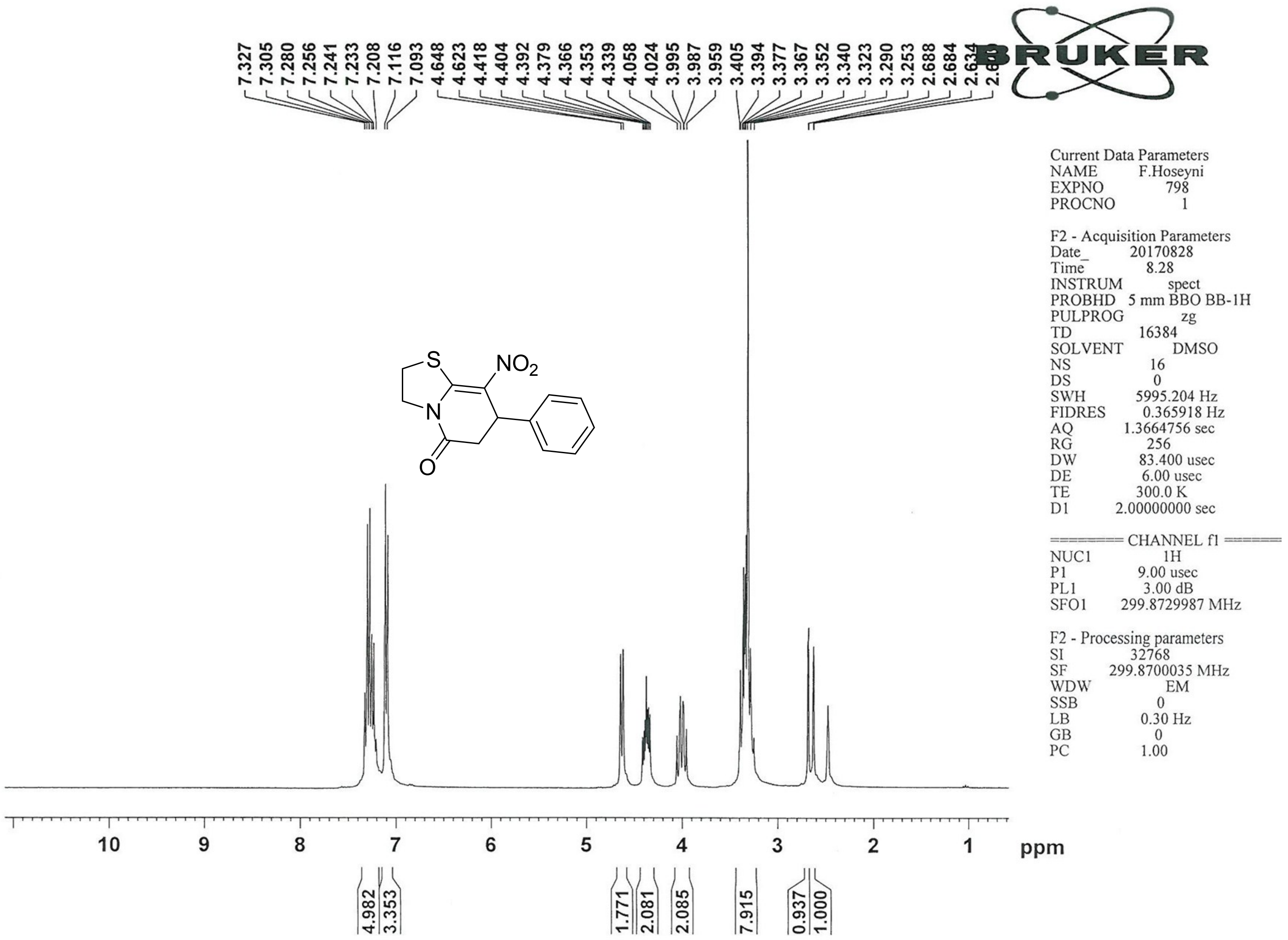
7.327
7.305
7.280
7.256
7.241
7.233
7.208
7.116
7.093
4.648
4.623
4.418
4.404
4.392
4.379
4.366
4.353
4.339
4.058
4.024
3.995
3.987
3.959
3.405
3.394
3.377
3.367
3.352
3.340
3.323
3.290
3.253
2.688
2.684
2.634
BRUKER
S
NO2
N
O
Current Data Parameters
NAME F.Hoseyni
EXPNO 798
PROCNO 1
F2 - Acquisition Parameters
Date_ 20170828
Time 8.28
INSTRUM spect
PROBHD 5 mm BBO BB-1H
PULPROG zg
TD 16384
SOLVENT DMSO
NS 16
DS 0
SWH 5995.204 Hz
FIDRES 0.365918 Hz
AQ 1.3664756 sec
RG 256
DW 83.400 usec
DE 6.00 usec
TE 300.0 K
D1 2.00000000 sec
======== CHANNEL f1 ========
NUC1 1H
P1 9.00 usec
PL1 3.00 dB
SFO1 299.8729987 MHz
F2 - Processing parameters
SI 32768
SF 299.8700035 MHz
WDW EM
SSB 0
LB 0.30 Hz
GB 0
PC 1.00
10
9
8
7
6
5
4
3
2
1
ppm
4.982
3.353
1.771
2.081
2.085
7.915
0.937
1.000

^{1}H NMR of 5g

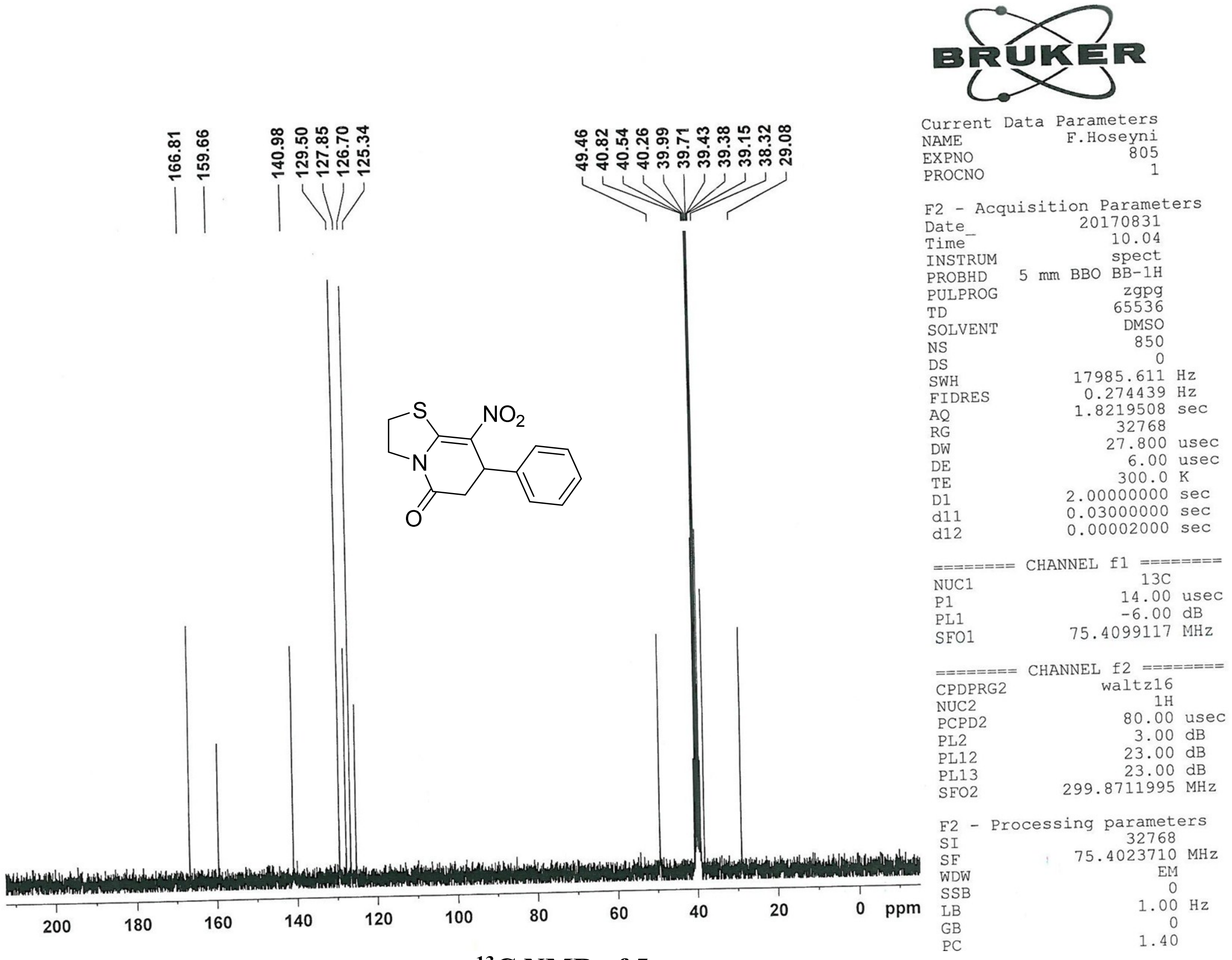
166.81
159.66
140.98
129.50
127.85
126.70
125.34
49.46
40.82
40.54
40.26
39.99
39.71
39.43
39.38
39.15
38.32
29.08
S
NO2
N
O
200 180 160 140 120 100 80 60 40 20 0 ppm
BRUKER
Current Data Parameters
NAME F.Hoseyni
EXPNO 805
PROCNO 1
F2 - Acquisition Parameters
Date_ 20170831
Time 10.04
INSTRUM spect
PROBHD 5 mm BBO BB-1H
PULPROG zgpg
TD 65536
SOLVENT DMSO
NS 850
DS 0
SWH 17985.611 Hz
FIDRES 0.274439 Hz
AQ 1.8219508 sec
RG 32768
DW 27.800 usec
DE 6.00 usec
TE 300.0 K
D1 2.00000000 sec
d11 0.03000000 sec
d12 0.00002000 sec
======== CHANNEL f1 ========
NUC1 13C
P1 14.00 usec
PL1 -6.00 dB
SFO1 75.4099117 MHz
======== CHANNEL f2 ========
CPDPRG2 waltz16
NUC2 1H
PCPD2 80.00 usec
PL2 3.00 dB
PL12 23.00 dB
PL13 23.00 dB
SFO2 299.8711995 MHz
F2 - Processing parameters
SI 32768
SF 75.4023710 MHz
WDW EM
SSB 0
LB 1.00 Hz
GB 0
PC 1.40

^{13}C NMR of 5g

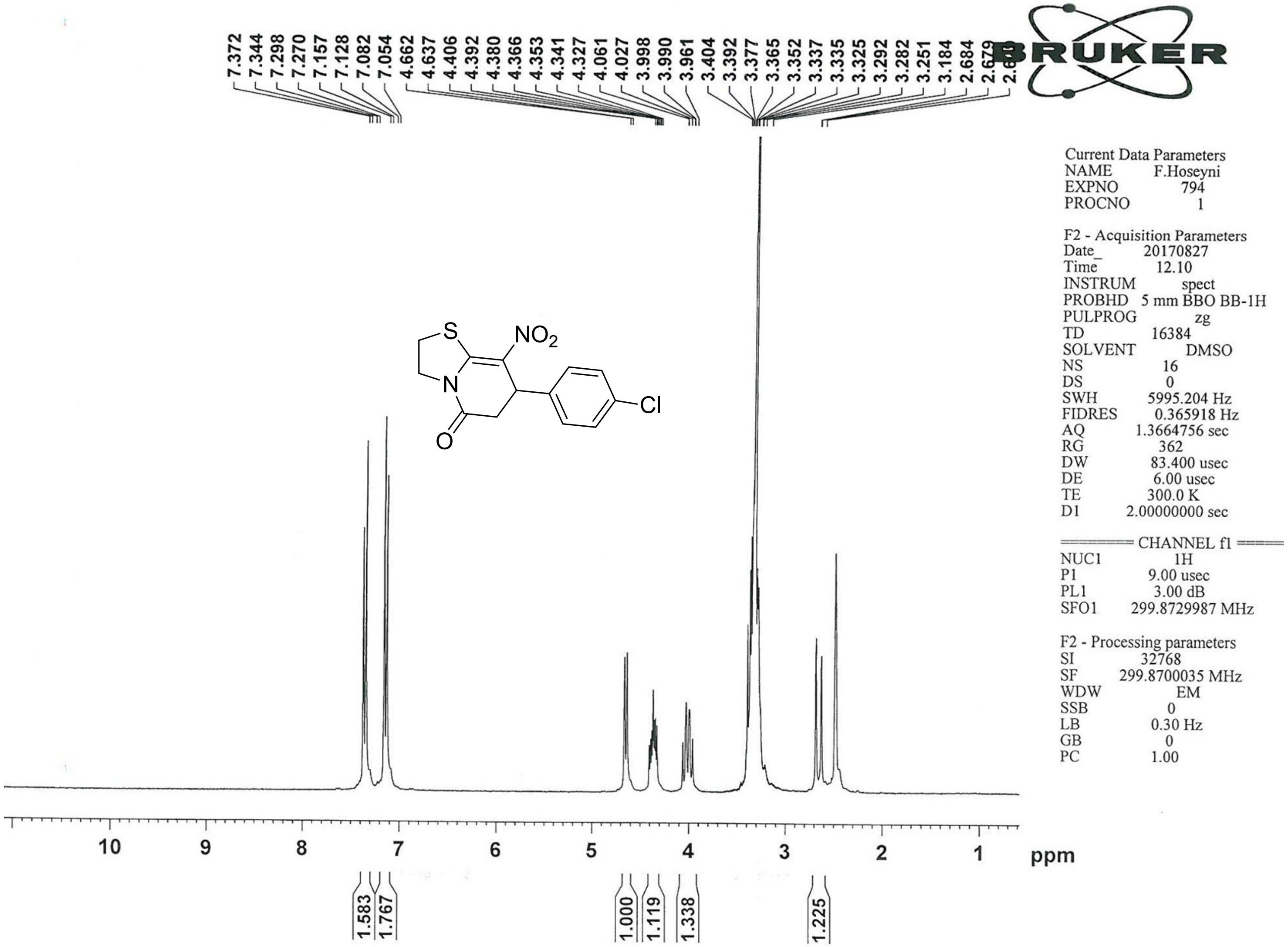
7.372
7.344
7.298
7.270
7.157
7.128
7.082
7.054
4.662
4.637
4.406
4.392
4.380
4.366
4.353
4.341
4.327
4.061
4.027
3.998
3.990
3.961
3.404
3.392
3.377
3.365
3.352
3.337
3.335
3.325
3.292
3.282
3.251
3.184
2.684
2.679
BRUKER
NO2
S
N
O
Cl
Current Data Parameters
NAME F.Hoseyni
EXPNO 794
PROCNO 1
F2 - Acquisition Parameters
Date_ 20170827
Time 12.10
INSTRUM spect
PROBHD 5 mm BBO BB-1H
PULPROG zg
TD 16384
SOLVENT DMSO
NS 16
DS 0
SWH 5995.204 Hz
FIDRES 0.365918 Hz
AQ 1.3664756 sec
RG 362
DW 83.400 usec
DE 6.00 usec
TE 300.0 K
D1 2.00000000 sec
CHANNEL f1
NUC1 1H
P1 9.00 usec
PL1 3.00 dB
SFO1 299.8729987 MHz
F2 - Processing parameters
SI 32768
SF 299.8700035 MHz
WDW EM
SSB 0
LB 0.30 Hz
GB 0
PC 1.00
10
9
8
7
6
5
4
3
2
1
ppm
1.583
1.767
1.000
1.119
1.338
1.225

^{1}H NMR of 5h

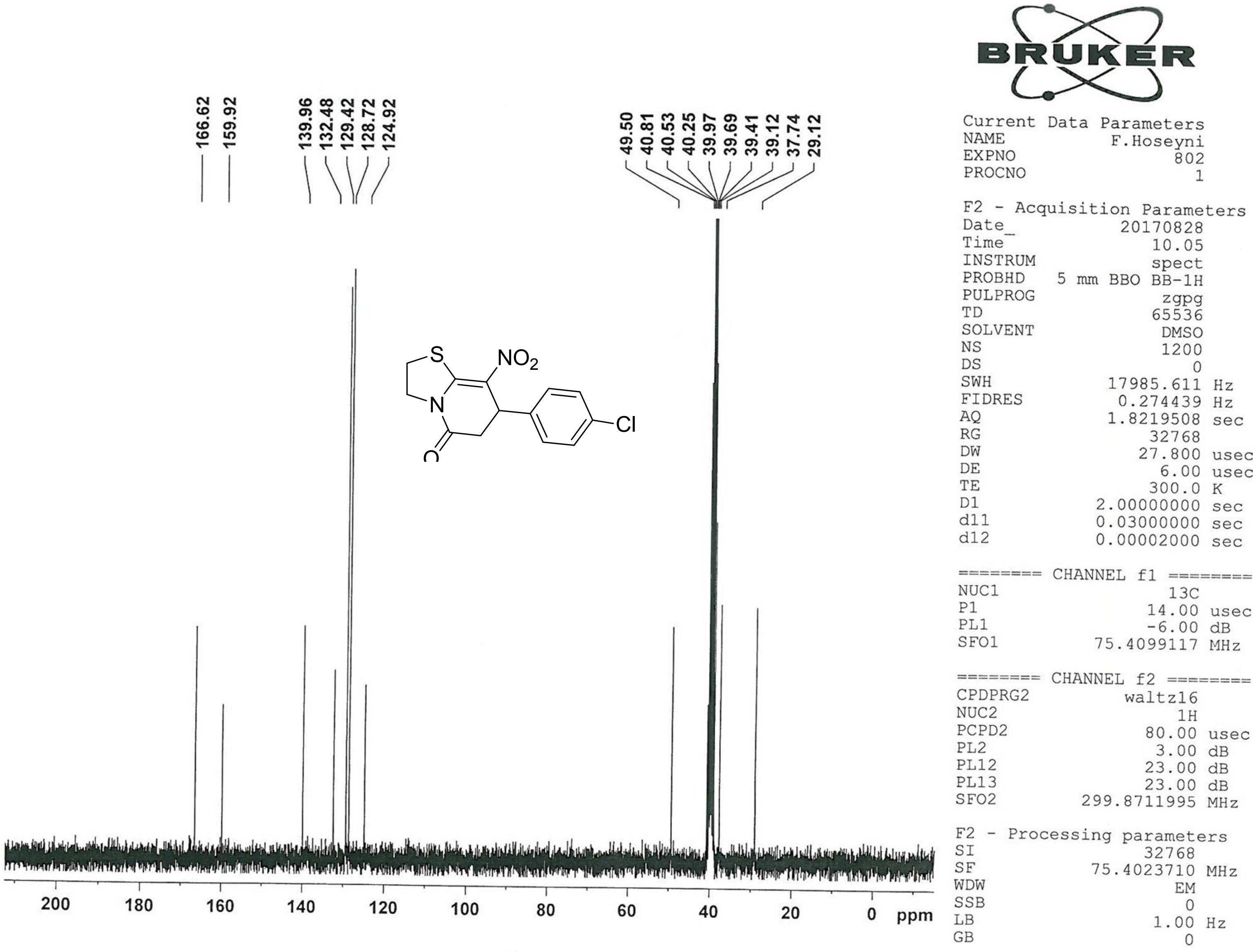
166.62
159.92
139.96
132.48
129.42
128.72
124.92
49.50
40.81
40.53
40.25
39.97
39.69
39.41
39.12
37.74
29.12
S
NO_2
N
Cl
O
200 180 160 140 120 100 80 60 40 20 0 ppm
BRUKER
Current Data Parameters
NAME F.Hoseyni
EXPNO 802
PROCNO 1
F2 - Acquisition Parameters
Date_ 20170828
Time 10.05
INSTRUM spect
PROBHD 5 mm BBO BB-1H
PULPROG zgpg
TD 65536
SOLVENT DMSO
NS 1200
DS 0
SWH 17985.611 Hz
FIDRES 0.274439 Hz
AQ 1.8219508 sec
RG 32768
DW 27.800 usec
DE 6.00 usec
TE 300.0 K
D1 2.00000000 sec
d11 0.03000000 sec
d12 0.00002000 sec
======== CHANNEL f1 ========
NUC1 13C
P1 14.00 usec
PL1 -6.00 dB
SFO1 75.4099117 MHz
======== CHANNEL f2 ========
CPDPRG2 waltz16
NUC2 1H
PCPD2 80.00 usec
PL2 3.00 dB
PL12 23.00 dB
PL13 23.00 dB
SFO2 299.8711995 MHz
F2 - Processing parameters
SI 32768
SF 75.4023710 MHz
WDW EM
SSB 0
LB 1.00 Hz
GB 0

^{13}C NMR of 5h

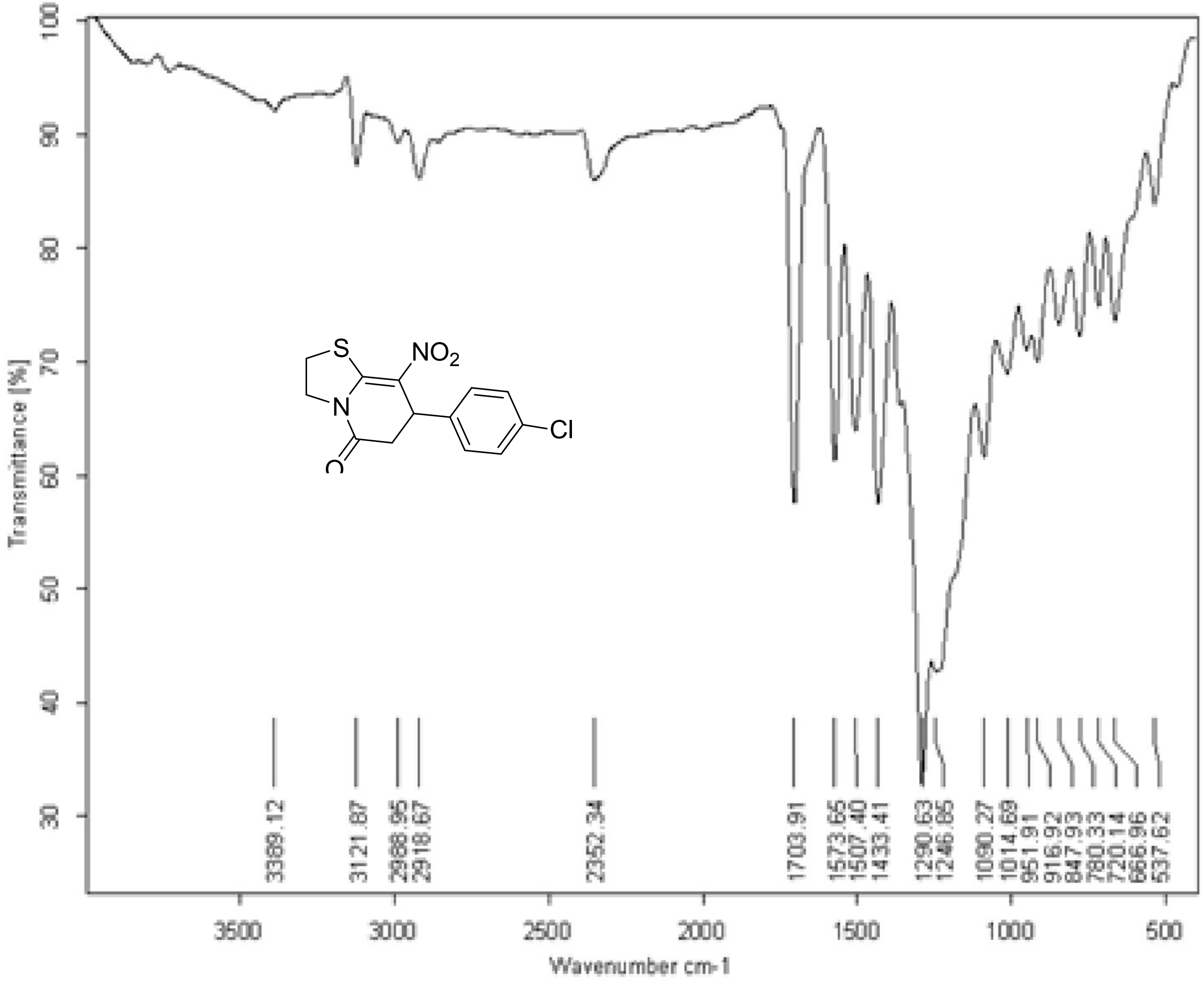

100
90
80
70
60
50
40
30
Transmittance [%]
3389.12
3121.87
2988.95
2918.67
2352.34
1703.91
1573.65
1507.40
1433.41
1290.63
1246.85
1090.27
1014.69
951.91
916.92
847.93
780.33
720.14
666.96
537.62
3500
3000
2500
2000
1500
1000
500
Wavenumber cm-1
S
NO2
N
O
Cl

IR of 5h

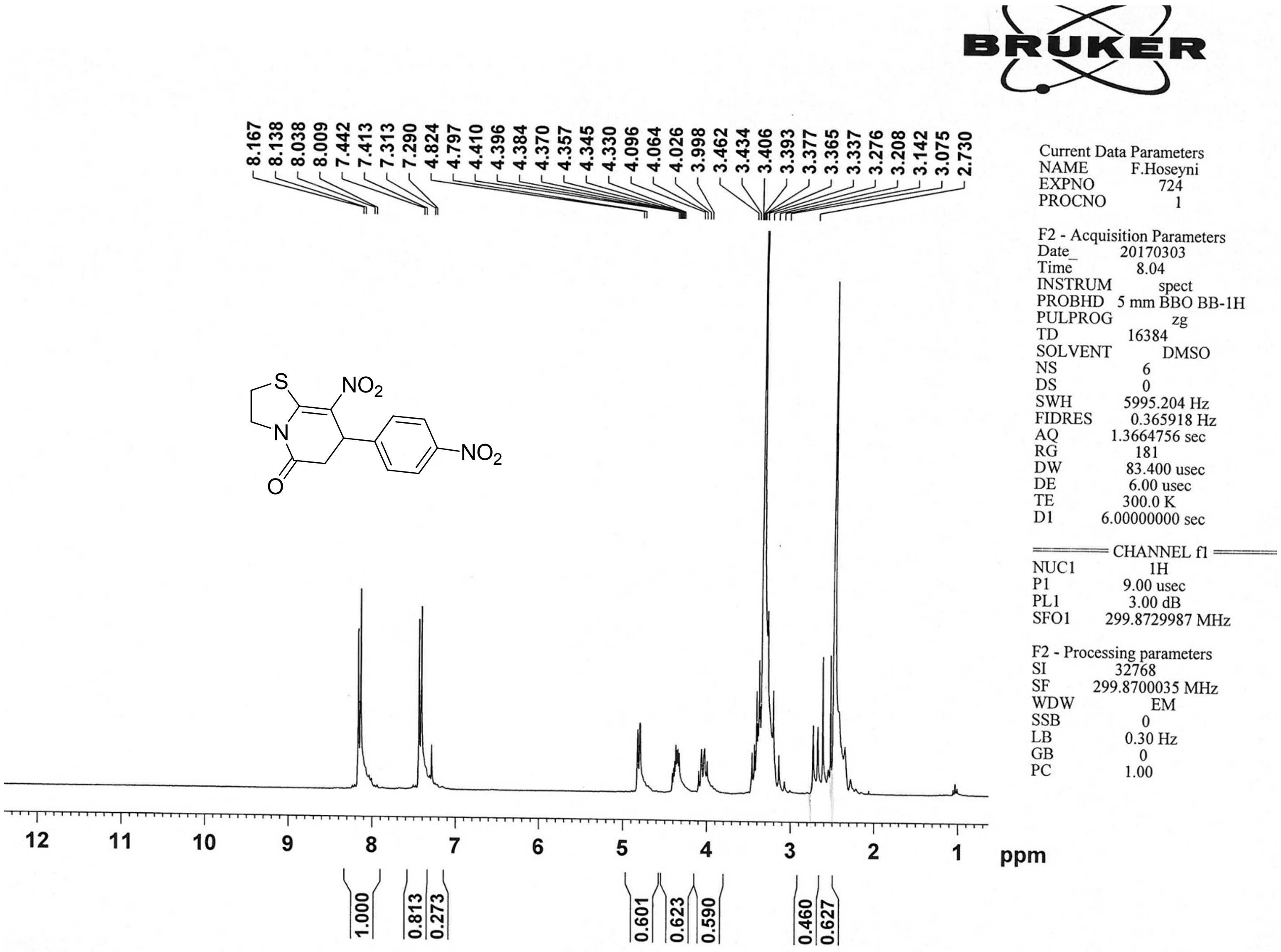
BRUKER
8.167
8.138
8.038
8.009
7.442
7.413
7.313
7.290
4.824
4.797
4.410
4.396
4.384
4.370
4.357
4.345
4.330
4.096
4.064
4.026
3.998
3.462
3.434
3.406
3.393
3.377
3.365
3.337
3.276
3.208
3.142
3.075
2.730
Current Data Parameters
NAME F.Hoseyni
EXPNO 724
PROCNO 1
F2 - Acquisition Parameters
Date_ 20170303
Time 8.04
INSTRUM spect
PROBHD 5 mm BBO BB-1H
PULPROG zg
TD 16384
SOLVENT DMSO
NS 6
DS 0
SWH 5995.204 Hz
FIDRES 0.365918 Hz
AQ 1.3664756 sec
RG 181
DW 83.400 usec
DE 6.00 usec
TE 300.0 K
D1 6.00000000 sec
CHANNEL f1
NUC1 1H
P1 9.00 usec
PL1 3.00 dB
SFO1 299.8729987 MHz
F2 - Processing parameters
SI 32768
SF 299.8700035 MHz
WDW EM
SSB 0
LB 0.30 Hz
GB 0
PC 1.00
S
N
O
NO2
NO2
12 11 10 9 8 7 6 5 4 3 2 1 ppm
1.000
0.813
0.273
0.601
0.623
0.590
0.460
0.627

^{1}H NMR of 5i

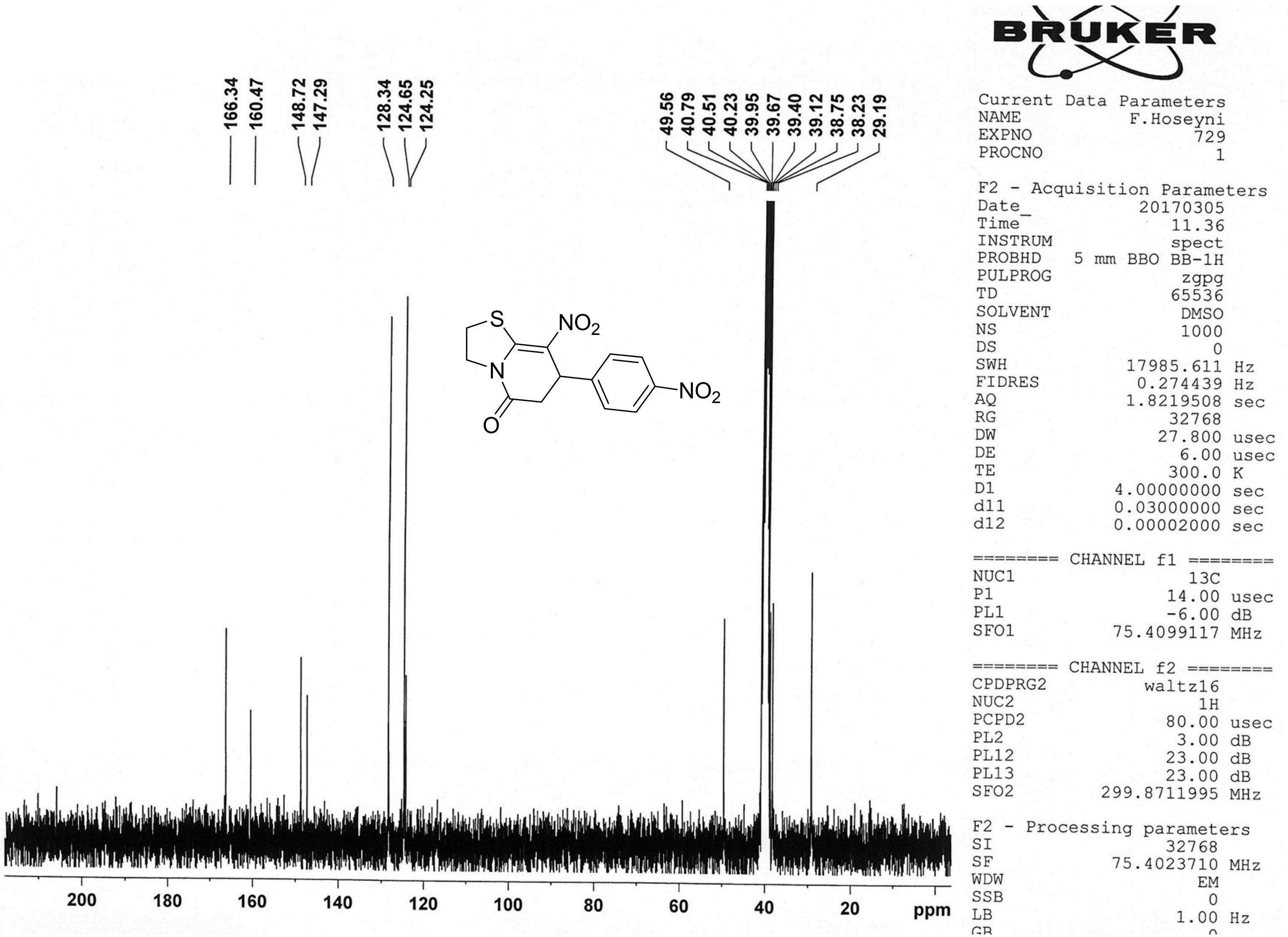
166.34
160.47
148.72
147.29
128.34
124.65
124.25
49.56
40.79
40.51
40.23
39.95
39.67
39.40
39.12
38.75
38.23
29.19
S
NO2
N
NO2
O
200
180
160
140
120
100
80
60
40
20
ppm
BRUKER
Current Data Parameters
NAME F.Hoseyni
EXPNO 729
PROCNO 1
F2 - Acquisition Parameters
Date_ 20170305
Time 11.36
INSTRUM spect
PROBHD 5 mm BBO BB-1H
PULPROG zgpg
TD 65536
SOLVENT DMSO
NS 1000
DS 0
SWH 17985.611 Hz
FIDRES 0.274439 Hz
AQ 1.8219508 sec
RG 32768
DW 27.800 usec
DE 6.00 usec
TE 300.0 K
D1 4.00000000 sec
d11 0.03000000 sec
d12 0.00002000 sec
======== CHANNEL f1 ========
NUC1 13C
P1 14.00 usec
PL1 -6.00 dB
SFO1 75.4099117 MHz
======== CHANNEL f2 ========
CPDPRG2 waltz16
NUC2 1H
PCPD2 80.00 usec
PL2 3.00 dB
PL12 23.00 dB
PL13 23.00 dB
SFO2 299.8711995 MHz
F2 - Processing parameters
SI 32768
SF 75.4023710 MHz
WDW EM
SSB 0
LB 1.00 Hz

^{13}C NMR of 5i

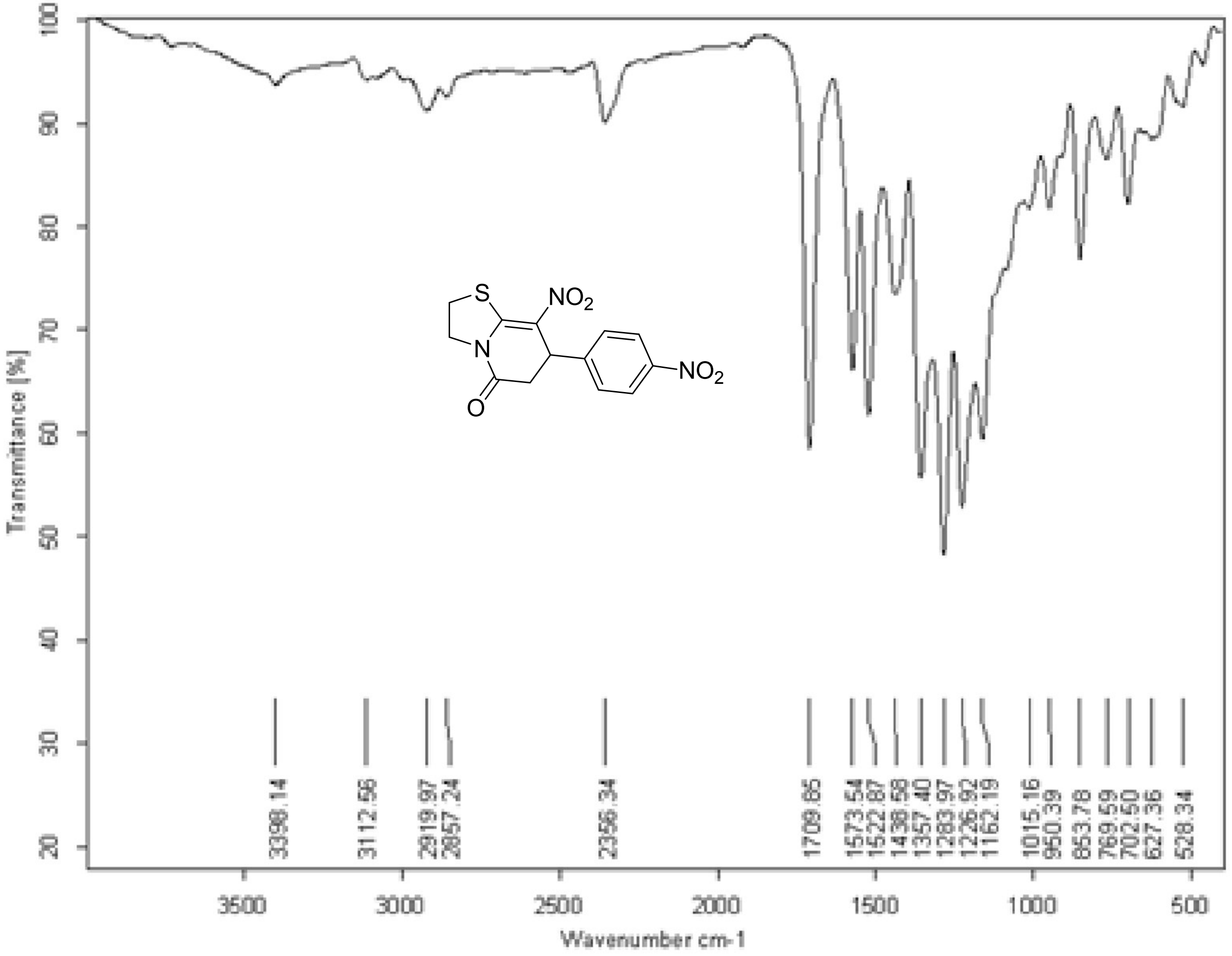

Transmittance [%]
Wavenumber cm-1
NO2
S
N
O
NO2
3398.14
3112.56
2919.97
2857.24
2356.34
1709.85
1573.54
1522.87
1438.58
1357.40
1283.97
1226.92
1162.19
1015.16
950.39
853.78
769.59
702.50
627.36
528.34

IR of 5i

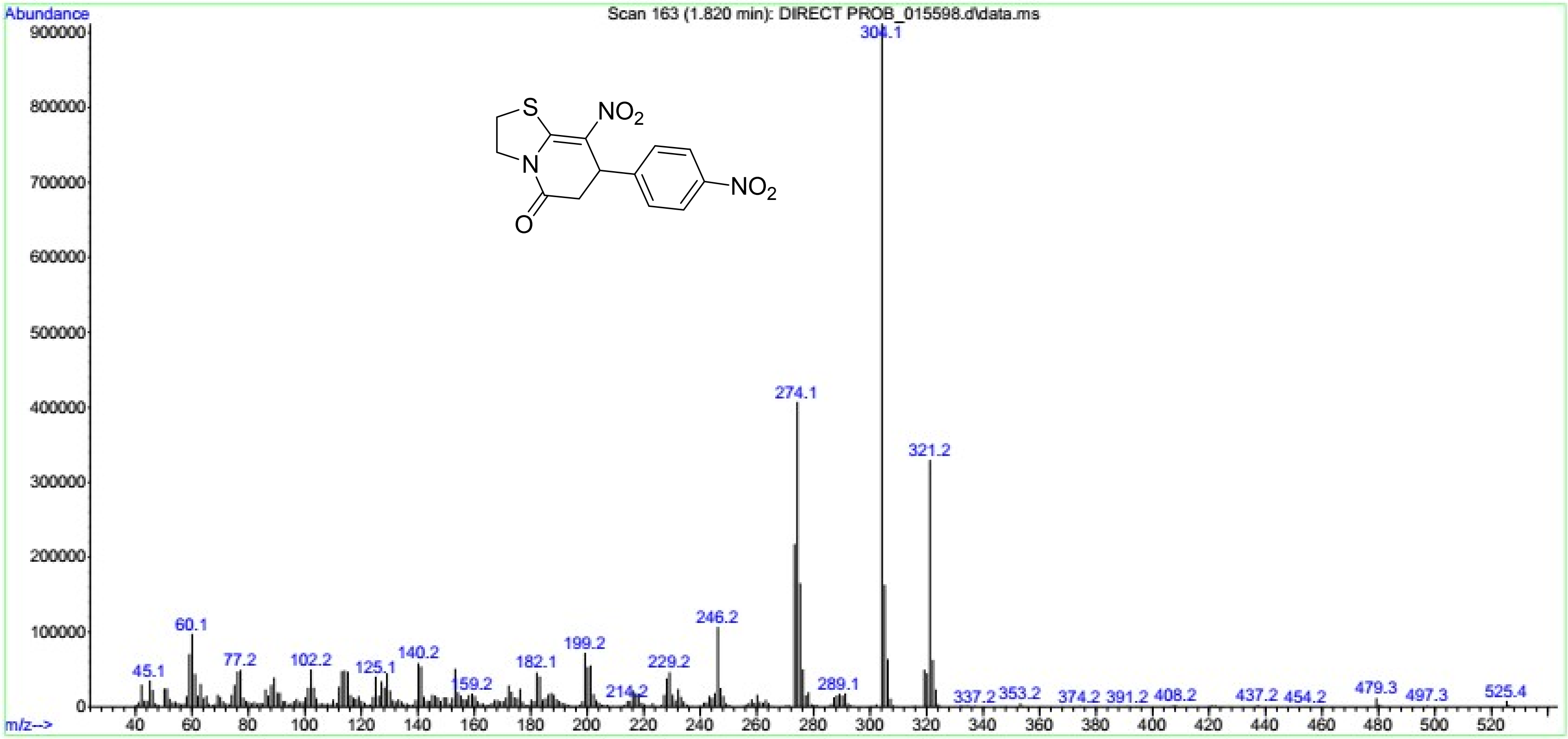
Abundance
Scan 163 (1.820 min): DIRECT PROB_015598.d\data.ms
304.1
274.1
321.2
246.2
60.1
45.1
77.2
102.2
125.1
140.2
159.2
182.1
199.2
214.2
229.2
289.1
337.2
353.2
374.2
391.2
408.2
437.2
454.2
479.3
497.3
525.4
m/z-->
NO2
S
N
O
NO2

Mass of 5i

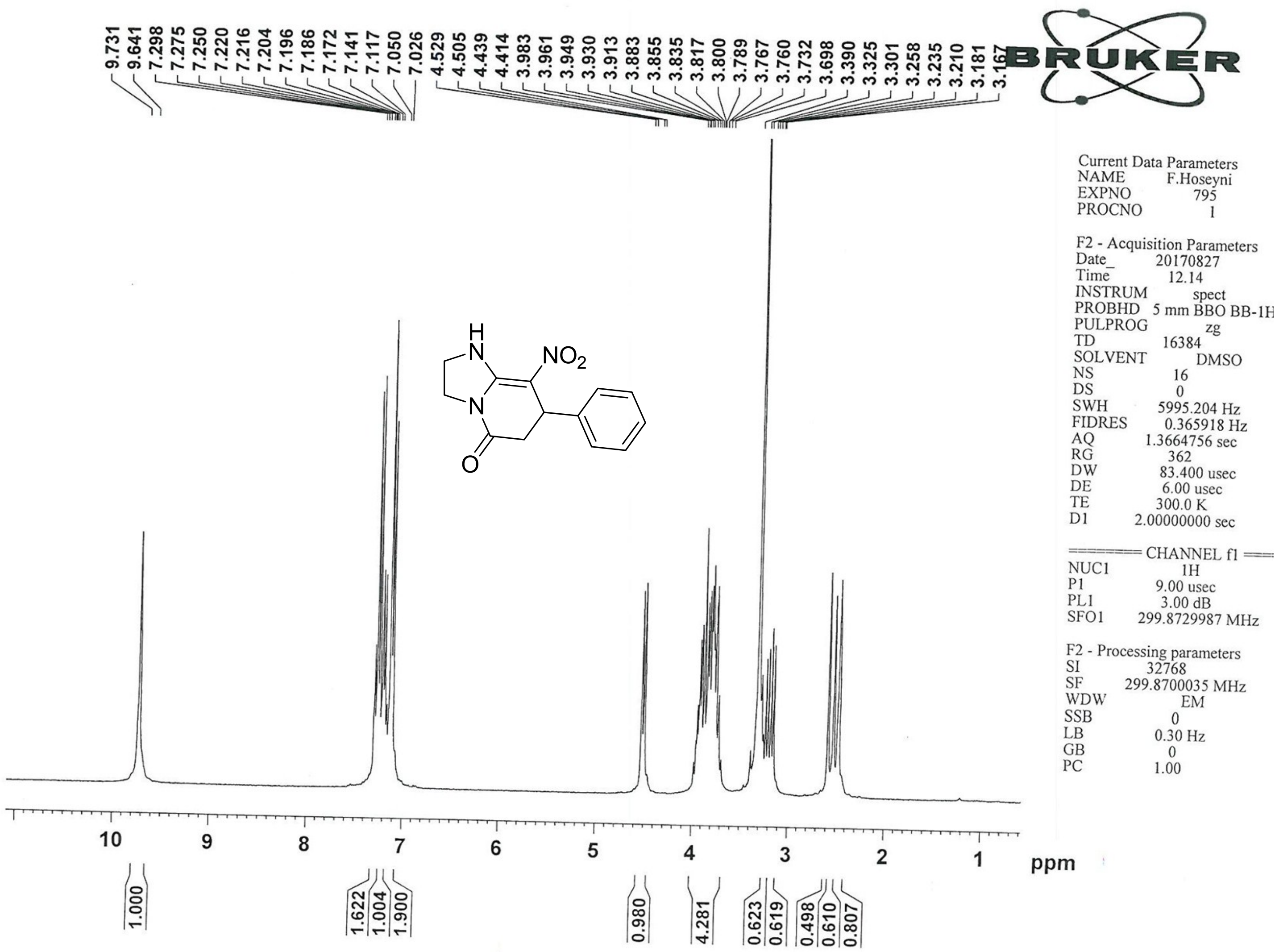

^{1}H NMR of 5j

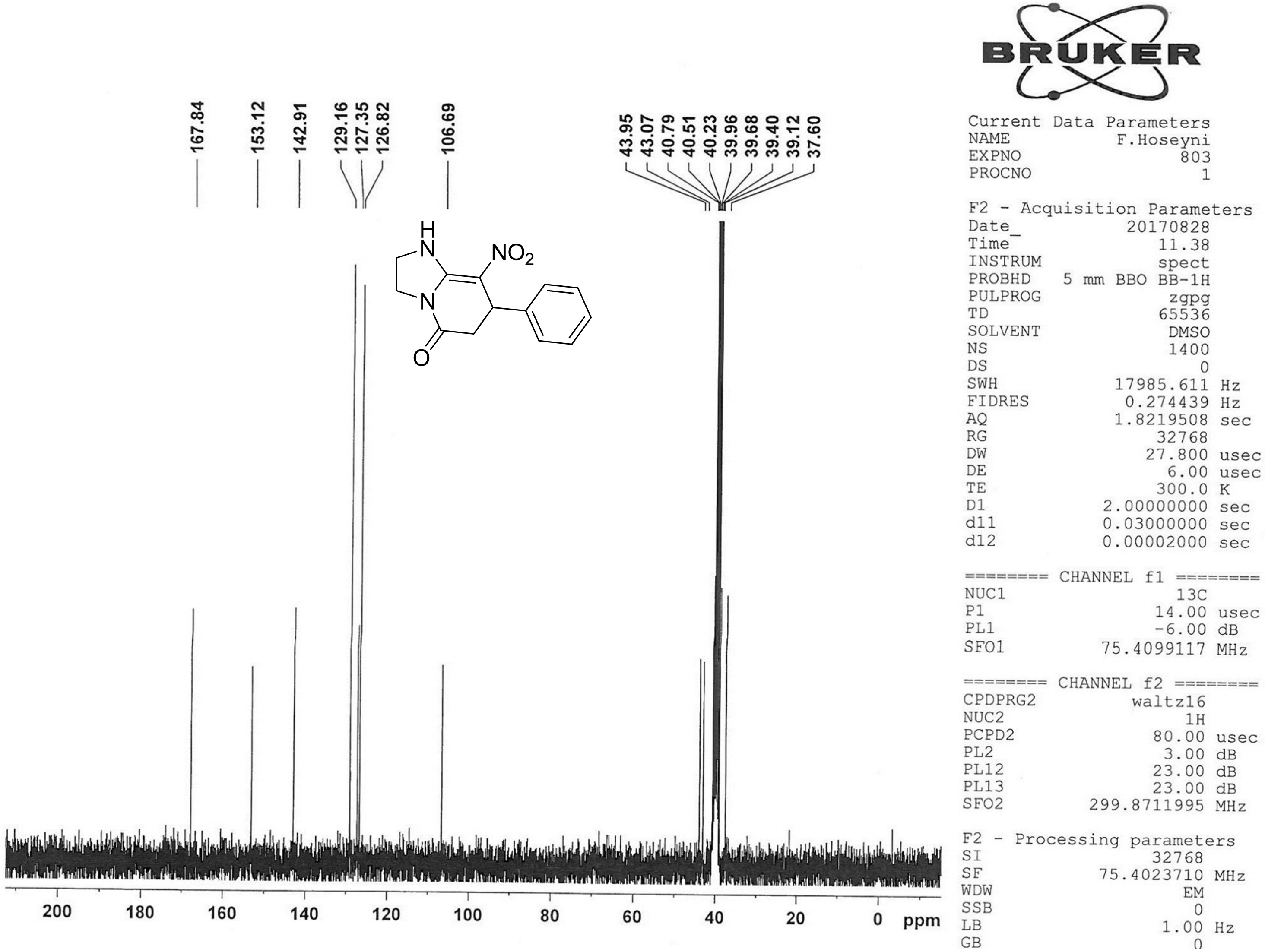

167.84
153.12
142.91
129.16
127.35
126.82
106.69
43.95
43.07
40.79
40.51
40.23
39.96
39.68
39.40
39.12
37.60
H
N
NO2
N
O
200 180 160 140 120 100 80 60 40 20 0 ppm
BRUKER
Current Data Parameters
NAME F.Hoseyni
EXPNO 803
PROCNO 1
F2 - Acquisition Parameters
Date_ 20170828
Time 11.38
INSTRUM spect
PROBHD 5 mm BBO BB-1H
PULPROG zgpg
TD 65536
SOLVENT DMSO
NS 1400
DS 0
SWH 17985.611 Hz
FIDRES 0.274439 Hz
AQ 1.8219508 sec
RG 32768
DW 27.800 usec
DE 6.00 usec
TE 300.0 K
D1 2.00000000 sec
d11 0.03000000 sec
d12 0.00002000 sec
======== CHANNEL f1 ========
NUC1 13C
P1 14.00 usec
PL1 -6.00 dB
SFO1 75.4099117 MHz
======== CHANNEL f2 ========
CPDPRG2 waltz16
NUC2 1H
PCPD2 80.00 usec
PL2 3.00 dB
PL12 23.00 dB
PL13 23.00 dB
SFO2 299.8711995 MHz
F2 - Processing parameters
SI 32768
SF 75.4023710 MHz
WDW EM
SSB 0
LB 1.00 Hz
GB 0

^{13}C NMR of 5j

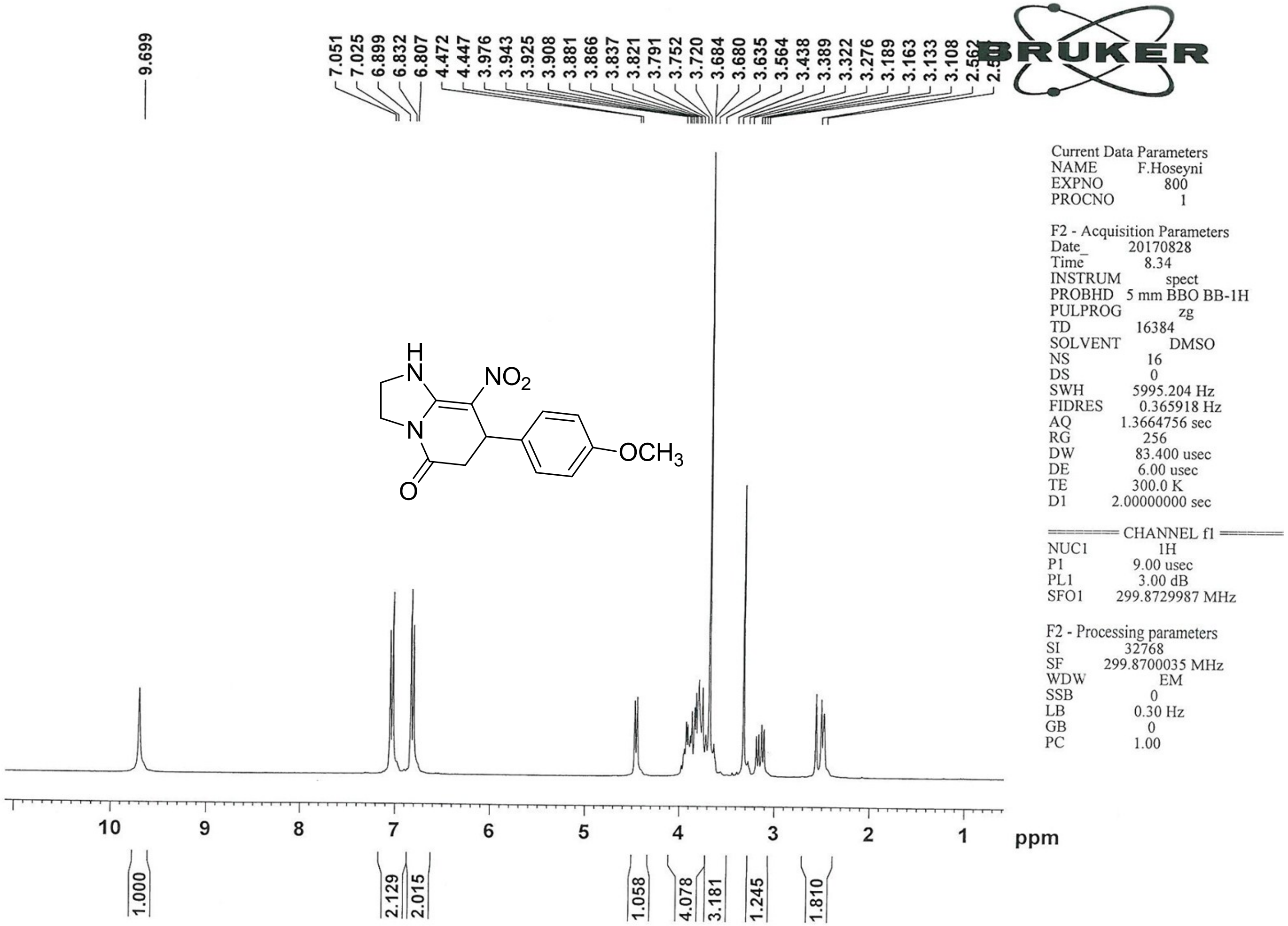
9.699
7.051
7.025
6.899
6.832
6.807
4.472
4.447
3.976
3.943
3.925
3.908
3.881
3.866
3.837
3.821
3.791
3.752
3.720
3.684
3.680
3.635
3.564
3.438
3.389
3.322
3.276
3.189
3.163
3.133
3.108
2.562
BRUKER
H
N
NO2
N
O
OCH3
Current Data Parameters
NAME F.Hoseyni
EXPNO 800
PROCNO 1
F2 - Acquisition Parameters
Date_ 20170828
Time 8.34
INSTRUM spect
PROBHD 5 mm BBO BB-1H
PULPROG zg
TD 16384
SOLVENT DMSO
NS 16
DS 0
SWH 5995.204 Hz
FIDRES 0.365918 Hz
AQ 1.3664756 sec
RG 256
DW 83.400 usec
DE 6.00 usec
TE 300.0 K
D1 2.00000000 sec
CHANNEL f1
NUC1 1H
P1 9.00 usec
PL1 3.00 dB
SFO1 299.8729987 MHz
F2 - Processing parameters
SI 32768
SF 299.8700035 MHz
WDW EM
SSB 0
LB 0.30 Hz
GB 0
PC 1.00
10
9
8
7
6
5
4
3
2
1
ppm
1.000
2.129
2.015
1.058
4.078
3.181
1.245
1.810

^{1}H NMR of 5k

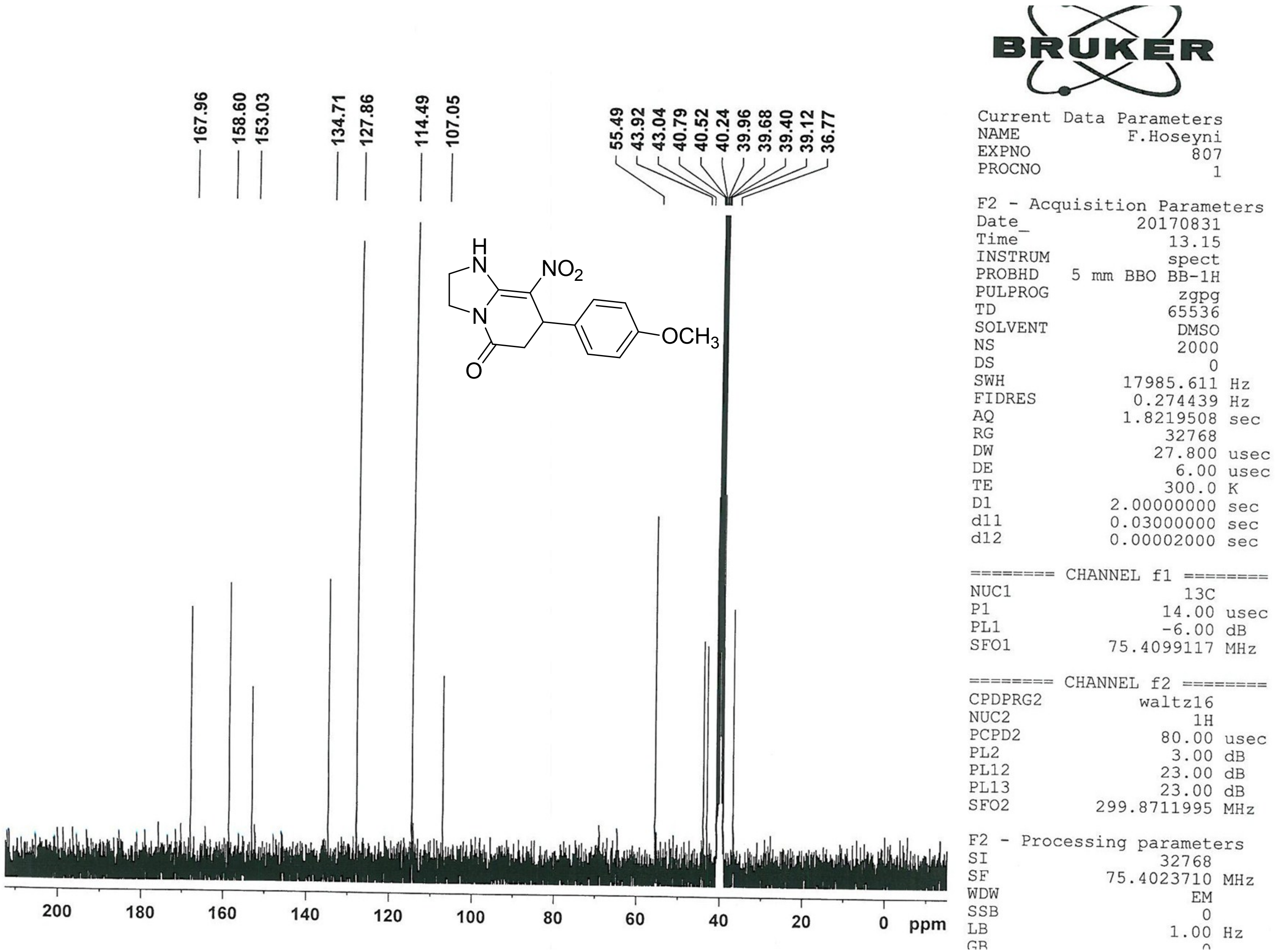
167.96
158.60
153.03
134.71
127.86
114.49
107.05
55.49
43.92
43.04
40.79
40.52
40.24
39.96
39.68
39.40
39.12
36.77
H
N
NO2
N
OCH3
O
200 180 160 140 120 100 80 60 40 20 0 ppm
BRUKER
Current Data Parameters
NAME F.Hoseyni
EXPNO 807
PROCNO 1
F2 - Acquisition Parameters
Date_ 20170831
Time 13.15
INSTRUM spect
PROBHD 5 mm BBO BB-1H
PULPROG zgpg
TD 65536
SOLVENT DMSO
NS 2000
DS 0
SWH 17985.611 Hz
FIDRES 0.274439 Hz
AQ 1.8219508 sec
RG 32768
DW 27.800 usec
DE 6.00 usec
TE 300.0 K
D1 2.00000000 sec
d11 0.03000000 sec
d12 0.00002000 sec
======== CHANNEL f1 ========
NUC1 13C
P1 14.00 usec
PL1 -6.00 dB
SFO1 75.4099117 MHz
======== CHANNEL f2 ========
CPDPRG2 waltz16
NUC2 1H
PCPD2 80.00 usec
PL2 3.00 dB
PL12 23.00 dB
PL13 23.00 dB
SFO2 299.8711995 MHz
F2 - Processing parameters
SI 32768
SF 75.4023710 MHz
WDW EM
SSB 0
LB 1.00 Hz

^{13}C NMR of 5k

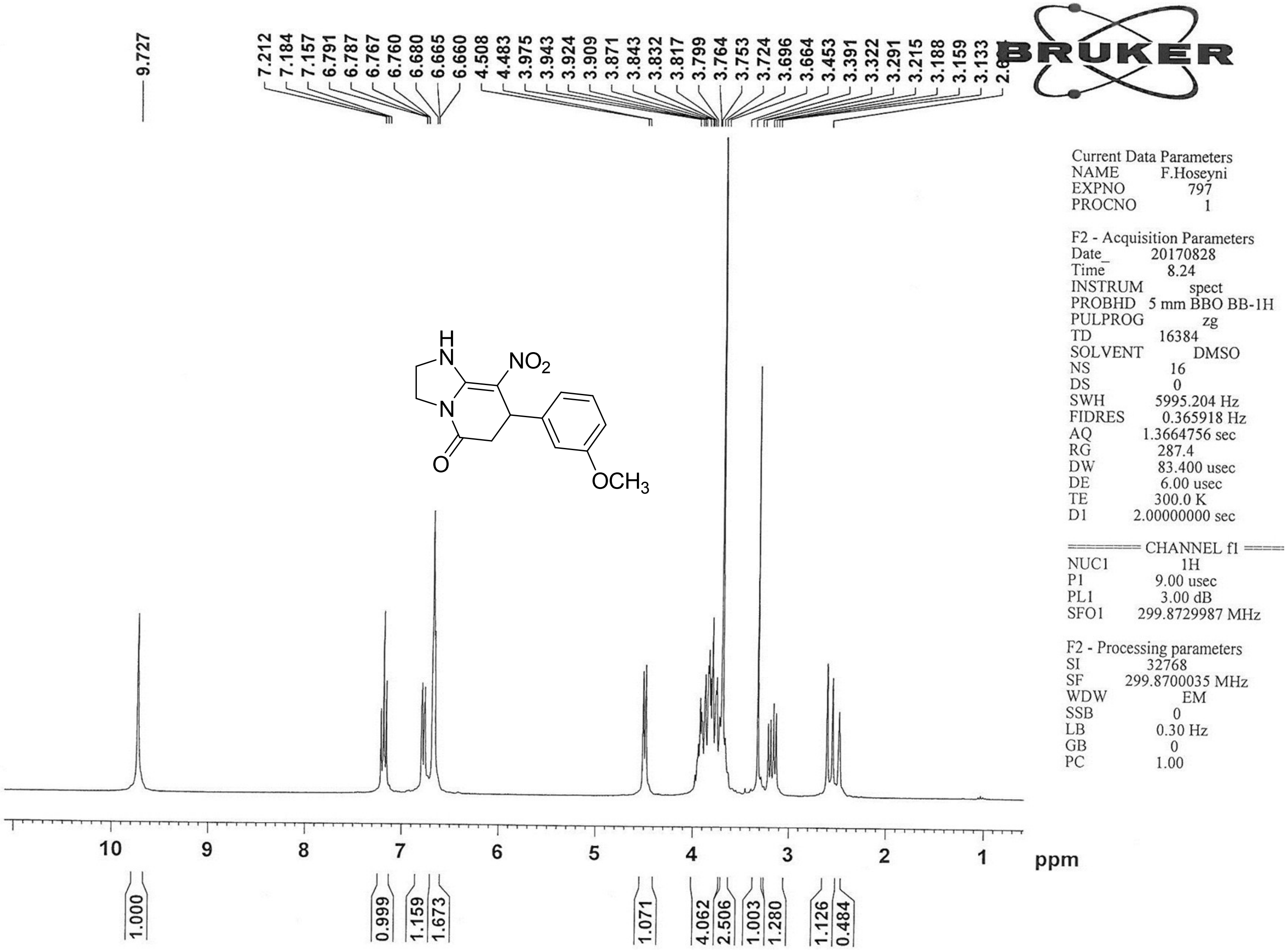
9.727
7.212
7.184
7.157
6.791
6.787
6.767
6.760
6.680
6.665
6.660
4.508
4.483
3.975
3.943
3.924
3.909
3.871
3.843
3.832
3.817
3.799
3.764
3.753
3.724
3.696
3.664
3.453
3.391
3.322
3.291
3.215
3.188
3.159
3.133
BRUKER
H
N
NO2
N
O
OCH3
Current Data Parameters
NAME F.Hoseyni
EXPNO 797
PROCNO 1
F2 - Acquisition Parameters
Date_ 20170828
Time 8.24
INSTRUM spect
PROBHD 5 mm BBO BB-1H
PULPROG zg
TD 16384
SOLVENT DMSO
NS 16
DS 0
SWH 5995.204 Hz
FIDRES 0.365918 Hz
AQ 1.3664756 sec
RG 287.4
DW 83.400 usec
DE 6.00 usec
TE 300.0 K
D1 2.00000000 sec
CHANNEL f1
NUC1 1H
P1 9.00 usec
PL1 3.00 dB
SFO1 299.8729987 MHz
F2 - Processing parameters
SI 32768
SF 299.8700035 MHz
WDW EM
SSB 0
LB 0.30 Hz
GB 0
PC 1.00
10
9
8
7
6
5
4
3
2
1
ppm
1.000
0.999
1.159
1.673
1.071
4.062
2.506
1.003
1.280
1.126
0.484

^{1}H NMR of 5l

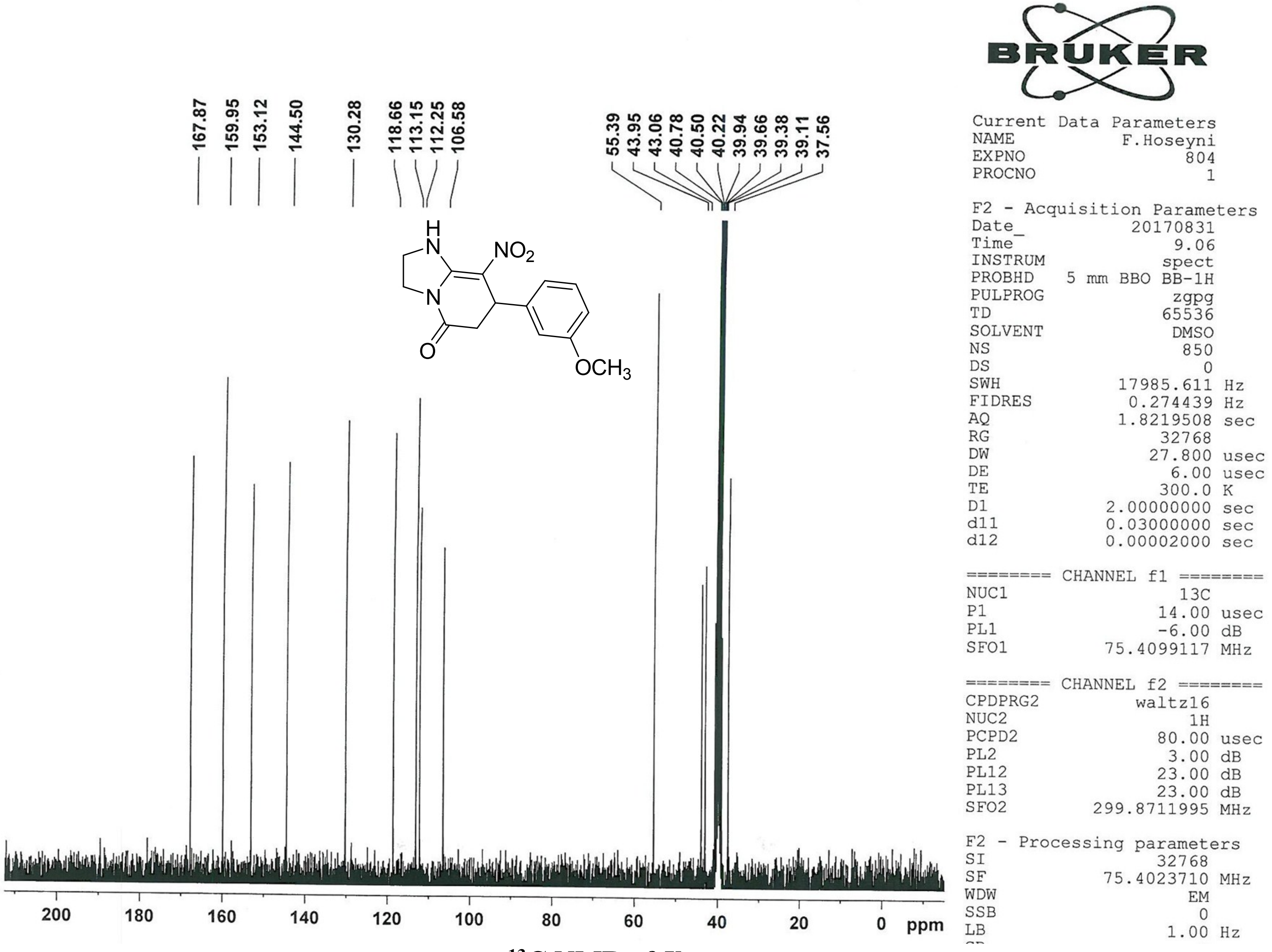
167.87
159.95
153.12
144.50
130.28
118.66
113.15
112.25
106.58
55.39
43.95
43.06
40.78
40.50
40.22
39.94
39.66
39.38
39.11
37.56
H
N
NO2
N
O
OCH3
200
180
160
140
120
100
80
60
40
20
0
ppm
BRUKER
Current Data Parameters
NAME F.Hoseyni
EXPNO 804
PROCNO 1
F2 - Acquisition Parameters
Date_ 20170831
Time 9.06
INSTRUM spect
PROBHD 5 mm BBO BB-1H
PULPROG zgpg
TD 65536
SOLVENT DMSO
NS 850
DS 0
SWH 17985.611 Hz
FIDRES 0.274439 Hz
AQ 1.8219508 sec
RG 32768
DW 27.800 usec
DE 6.00 usec
TE 300.0 K
D1 2.00000000 sec
d11 0.03000000 sec
d12 0.00002000 sec
======== CHANNEL f1 ========
NUC1 13C
P1 14.00 usec
PL1 -6.00 dB
SFO1 75.4099117 MHz
======== CHANNEL f2 ========
CPDPRG2 waltz16
NUC2 1H
PCPD2 80.00 usec
PL2 3.00 dB
PL12 23.00 dB
PL13 23.00 dB
SFO2 299.8711995 MHz
F2 - Processing parameters
SI 32768
SF 75.4023710 MHz
WDW EM
SSB 0
LB 1.00 Hz

^{13}C NMR of 5l

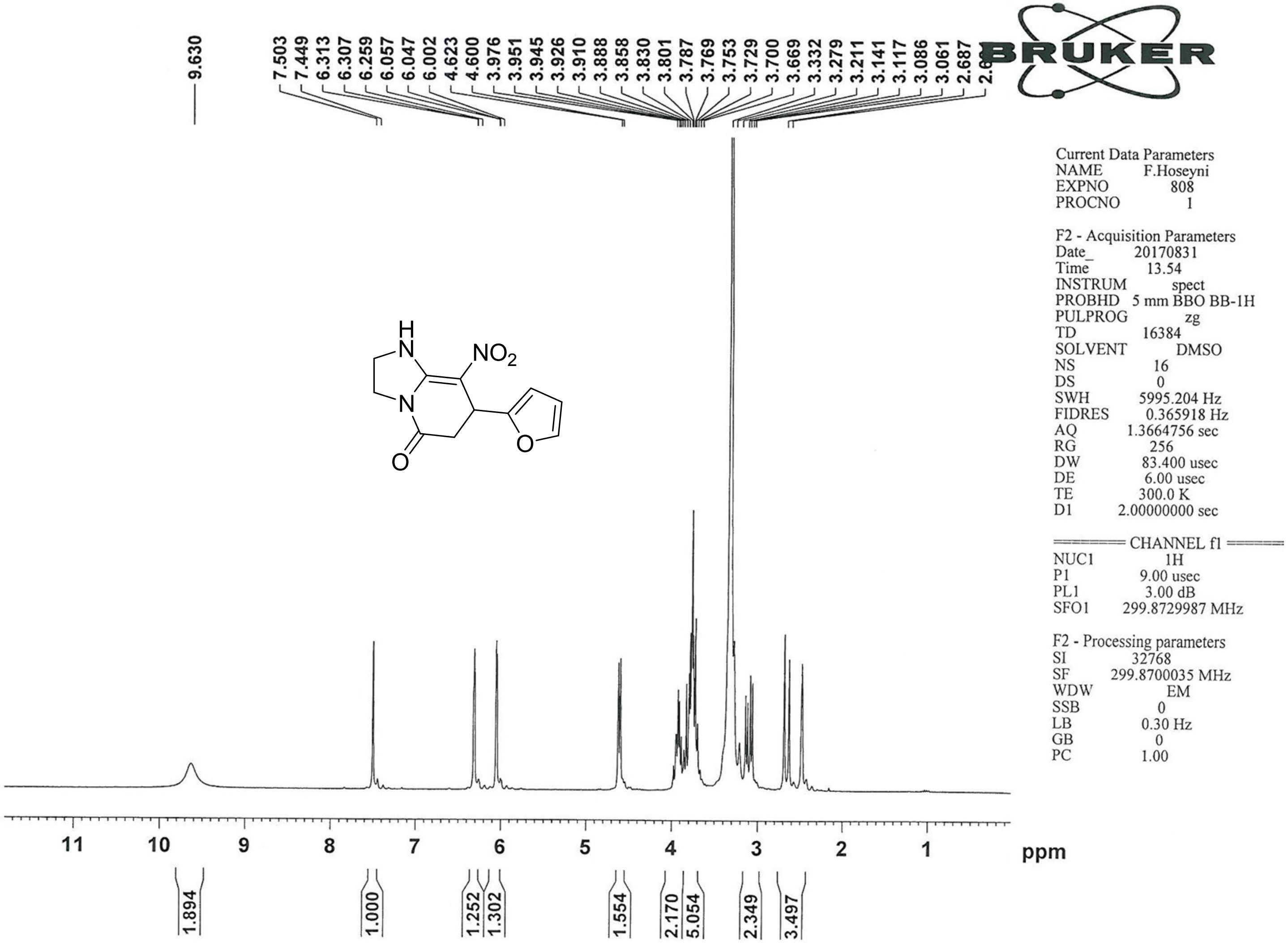
BRUKER
9.630
7.503 7.449 6.313 6.307 6.259 6.057 6.047 6.002 4.623 4.600 3.976 3.951 3.945 3.926 3.910 3.888 3.858 3.830 3.801 3.787 3.769 3.753 3.729 3.700 3.669 3.332 3.279 3.211 3.141 3.117 3.086 3.061 2.687
Current Data Parameters
NAME F.Hoseyni
EXPNO 808
PROCNO 1
F2 - Acquisition Parameters
Date_ 20170831
Time 13.54
INSTRUM spect
PROBHD 5 mm BBO BB-1H
PULPROG zg
TD 16384
SOLVENT DMSO
NS 16
DS 0
SWH 5995.204 Hz
FIDRES 0.365918 Hz
AQ 1.3664756 sec
RG 256
DW 83.400 usec
DE 6.00 usec
TE 300.0 K
D1 2.00000000 sec
CHANNEL f1
NUC1 1H
P1 9.00 usec
PL1 3.00 dB
SFO1 299.8729987 MHz
F2 - Processing parameters
SI 32768
SF 299.8700035 MHz
WDW EM
SSB 0
LB 0.30 Hz
GB 0
PC 1.00
H
N
NO2
N
O
O
11 10 9 8 7 6 5 4 3 2 1 ppm
1.894 1.000 1.252 1.302 1.554 2.170 5.054 2.349 3.497

^{1}H NMR of 5m

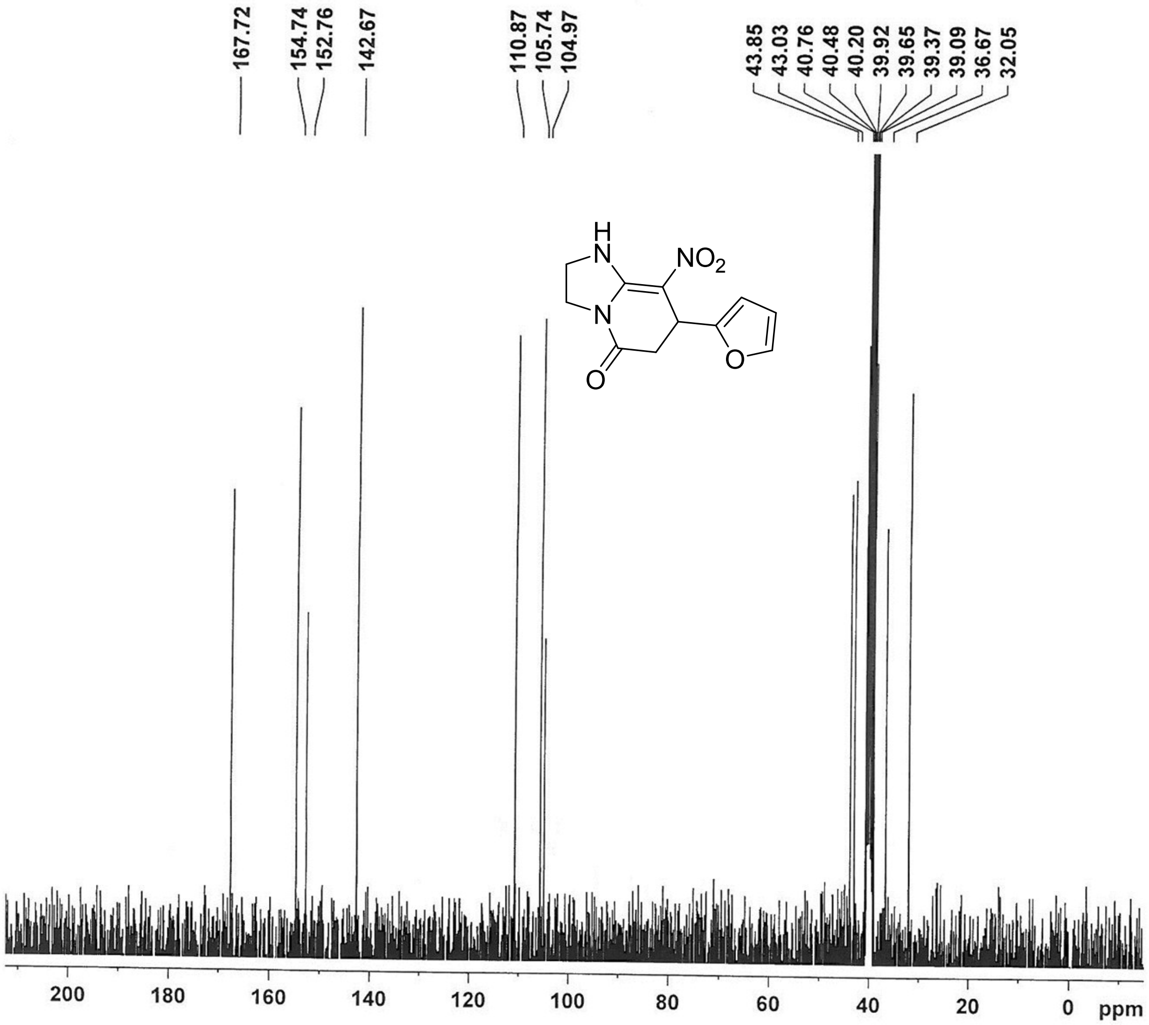

Current Data Parameters
NAME F.Hoseyni
EXPNO 810
PROCNO 1

F2 - Acquisition Parameters
Date_ 20170902
Time 8.58
INSTRUM spect
PROBHD 5 mm BBO BB-1H
PULPROG zgpg
TD 65536
SOLVENT DMSO
NS 751
DS 0
SWH 17985.611 Hz
FIDRES 0.274439 Hz
AQ 1.8219508 sec
RG 32768
DW 27.800 usec
DE 6.00 usec
TE 300.0 K
D1 2.00000000 sec
d11 0.03000000 sec
d12 0.00002000 sec

======== CHANNEL f1 ========
NUC1 13C
P1 14.00 usec
PL1 -6.00 dB
SFO1 75.4099117 MHz

======== CHANNEL f2 ========
CPDPRG2 waltz16
NUC2 1H
PCPD2 80.00 usec
PL2 3.00 dB
PL12 23.00 dB
PL13 23.00 dB
SFO2 299.8711995 MHz

F2 - Processing parameters
SI 32768
SF 75.4023710 MHz
WDW EM
SSB 0
LB 1.00 Hz

^{13}C NMR of 5m

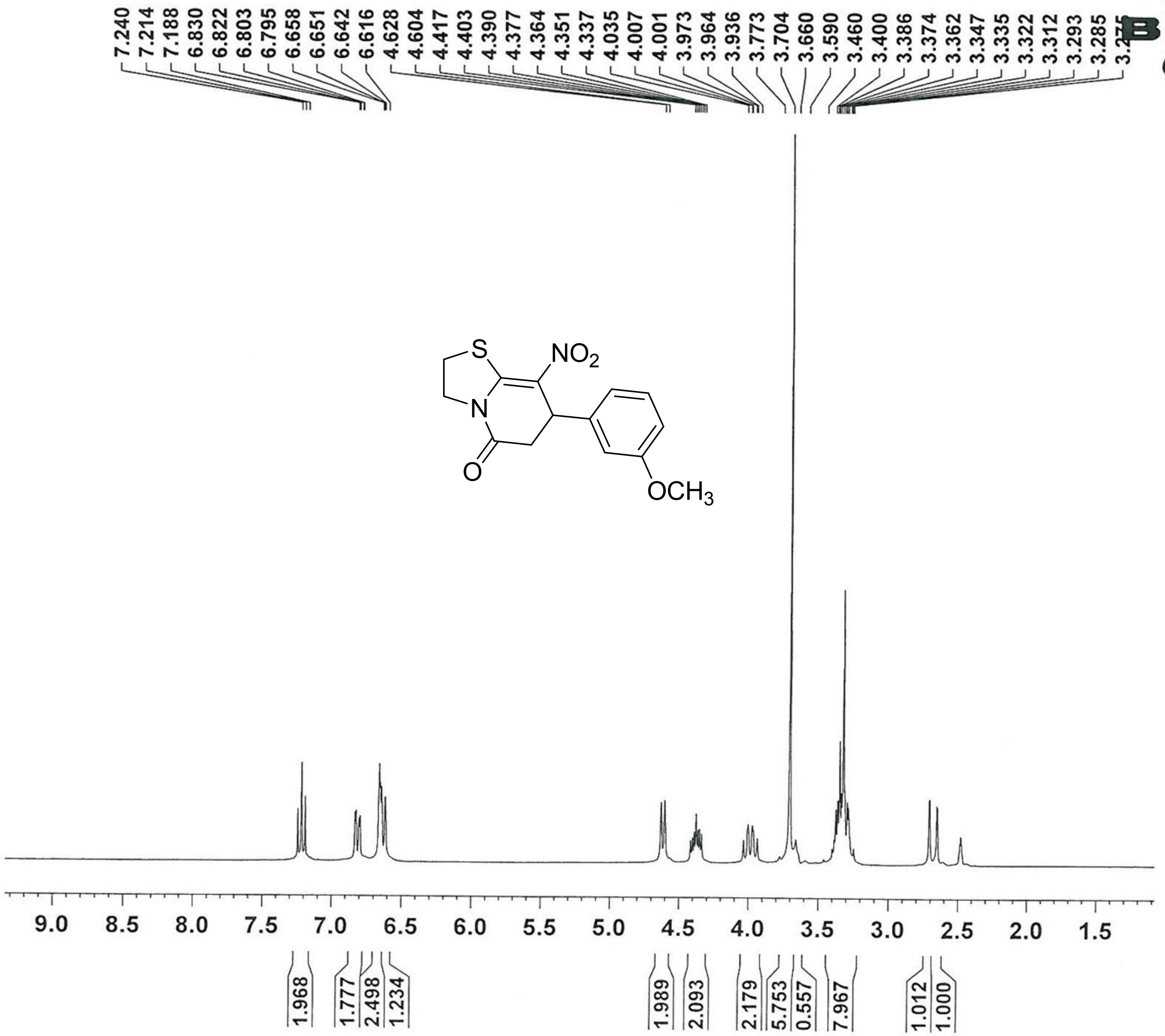

Current Data Parameters
NAME F.Hoseyni
EXPNO 799
PROCNO 1

F2 - Acquisition Parameters
Date_ 20170828
Time 8.42
INSTRUM spect
PROBHD 5 mm BBO BB-1H
PULPROG zg
TD 16384
SOLVENT DMSO
NS 16
DS 0
SWH 5995.204 Hz
FIDRES 0.365918 Hz
AQ 1.3664756 sec
RG 256
DW 83.400 usec
DE 6.00 usec
TE 300.0 K
D1 2.00000000 sec

======== CHANNEL f1 ========
NUC1 1H
P1 9.00 usec
PL1 3.00 dB
SFO1 299.8729987 MHz

F2 - Processing parameters
SI 32768
SF 299.8700035 MHz
WDW EM
SSB 0
LB 0.30 Hz
GB 0
PC 1.00

^{1}H NMR of 5n

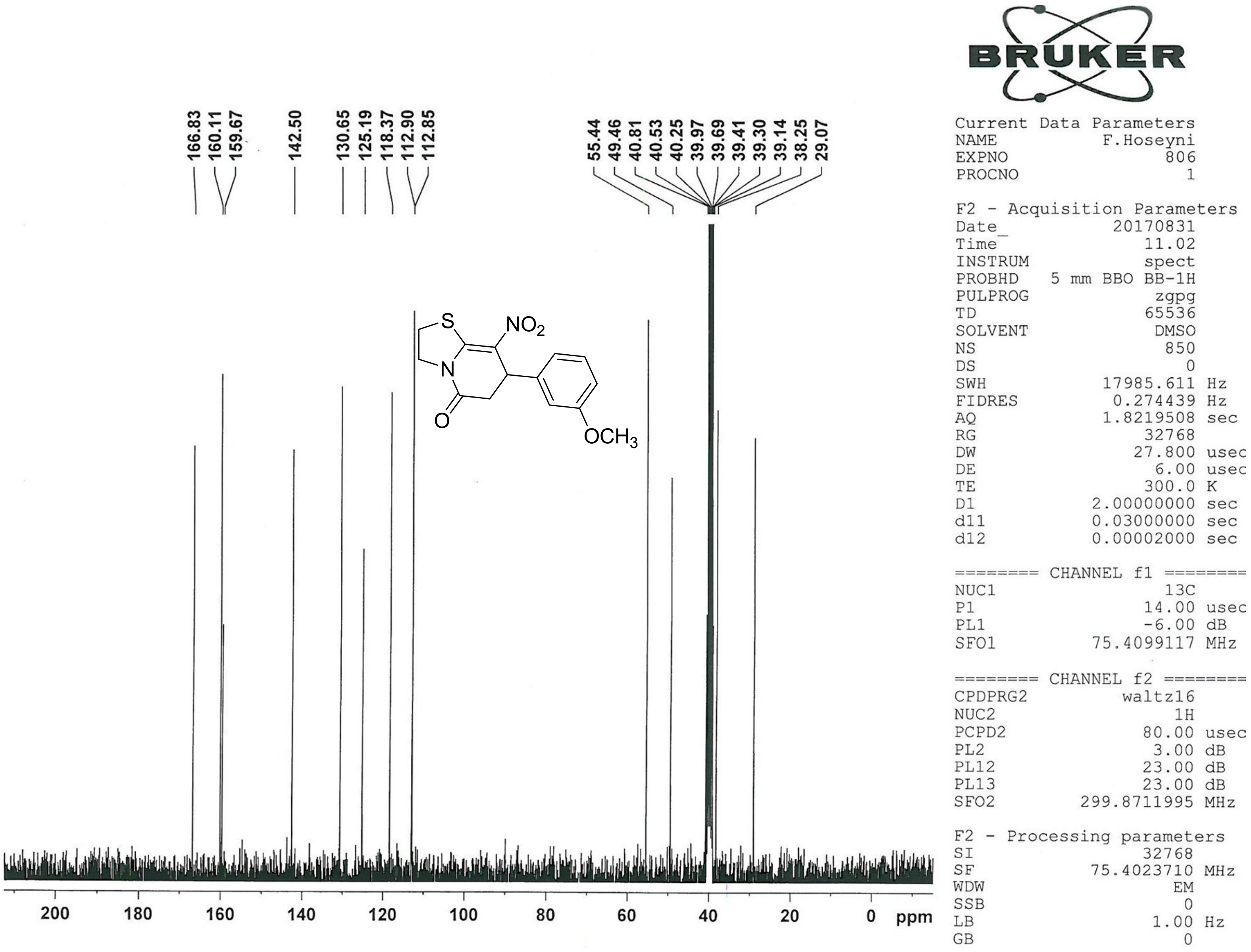
166.83
160.11
159.67
142.50
130.65
125.19
118.37
112.90
112.85
55.44
49.46
40.81
40.53
40.25
39.97
39.69
39.41
39.30
39.14
38.25
29.07
S
NO2
N
O
OCH3
200 180 160 140 120 100 80 60 40 20 0 ppm
BRUKER
Current Data Parameters
NAME F.Hoseyni
EXPNO 806
PROCNO 1
F2 - Acquisition Parameters
Date_ 20170831
Time 11.02
INSTRUM spect
PROBHD 5 mm BBO BB-1H
PULPROG zgpg
TD 65536
SOLVENT DMSO
NS 850
DS 0
SWH 17985.611 Hz
FIDRES 0.274439 Hz
AQ 1.8219508 sec
RG 32768
DW 27.800 usec
DE 6.00 usec
TE 300.0 K
D1 2.00000000 sec
d11 0.03000000 sec
d12 0.00002000 sec
======== CHANNEL f1 ========
NUC1 13C
P1 14.00 usec
PL1 -6.00 dB
SFO1 75.4099117 MHz
======== CHANNEL f2 ========
CPDPRG2 waltz16
NUC2 1H
PCPD2 80.00 usec
PL2 3.00 dB
PL12 23.00 dB
PL13 23.00 dB
SFO2 299.8711995 MHz
F2 - Processing parameters
SI 32768
SF 75.4023710 MHz
WDW EM
SSB 0
LB 1.00 Hz
GB 0

^{13}C NMR of 5n

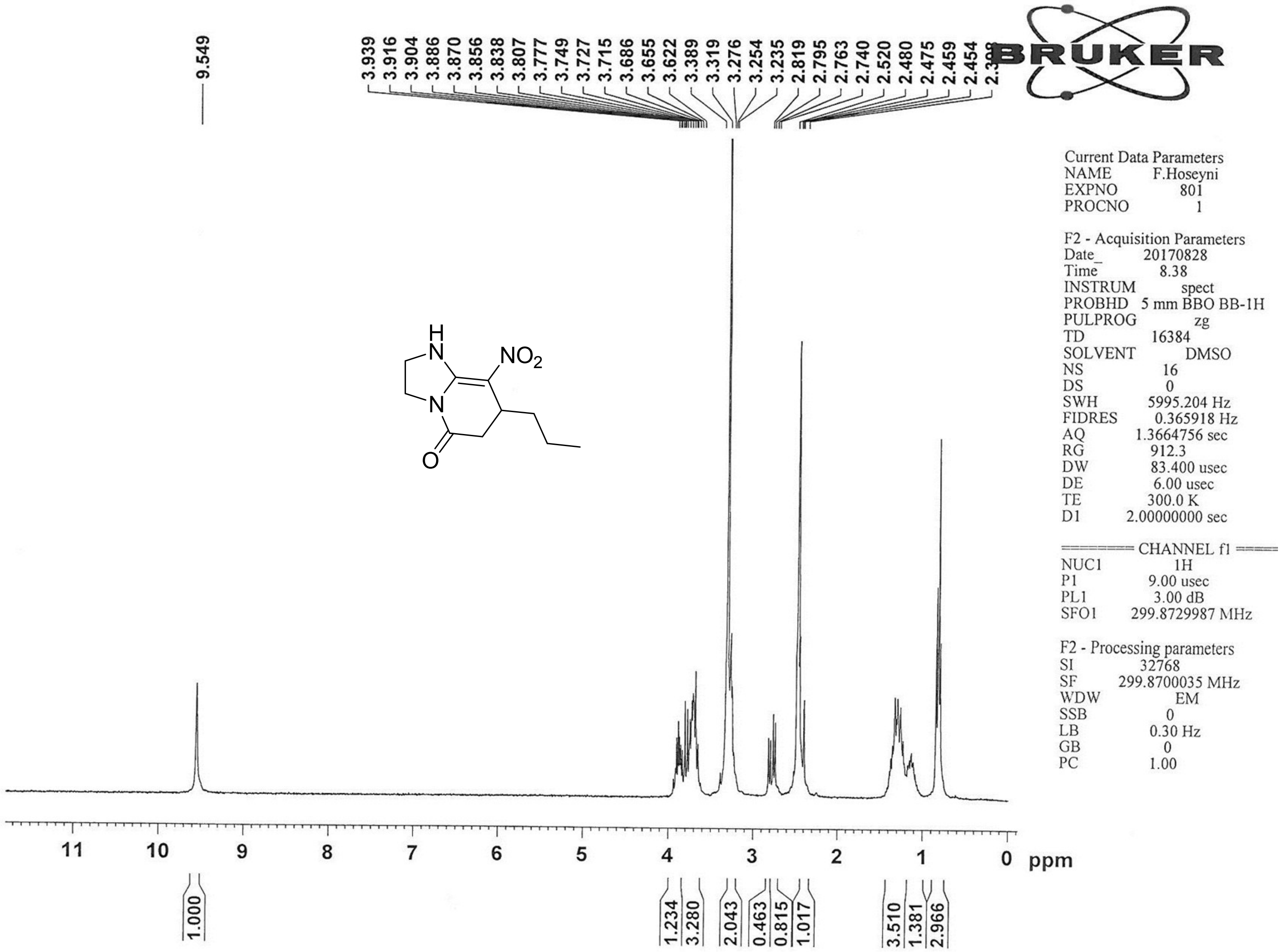

9.549
3.939
3.916
3.904
3.886
3.870
3.856
3.838
3.807
3.777
3.749
3.727
3.715
3.686
3.655
3.622
3.389
3.319
3.276
3.254
3.235
2.819
2.795
2.763
2.740
2.520
2.480
2.475
2.459
2.454
BRUKER
Current Data Parameters
NAME F.Hoseyni
EXPNO 801
PROCNO 1
F2 - Acquisition Parameters
Date_ 20170828
Time 8.38
INSTRUM spect
PROBHD 5 mm BBO BB-1H
PULPROG zg
TD 16384
SOLVENT DMSO
NS 16
DS 0
SWH 5995.204 Hz
FIDRES 0.365918 Hz
AQ 1.3664756 sec
RG 912.3
DW 83.400 usec
DE 6.00 usec
TE 300.0 K
D1 2.00000000 sec
CHANNEL f1
NUC1 1H
P1 9.00 usec
PL1 3.00 dB
SFO1 299.8729987 MHz
F2 - Processing parameters
SI 32768
SF 299.8700035 MHz
WDW EM
SSB 0
LB 0.30 Hz
GB 0
PC 1.00
H
N
NO2
N
O
11 10 9 8 7 6 5 4 3 2 1 0 ppm
1.000
1.234
3.280
2.043
0.463
0.815
1.017
3.510
1.381
2.966

^{1}H NMR of 5o
